# White matter microstructural alterations across four major psychiatric disorders: mega-analysis study in 2937 individuals

**DOI:** 10.1038/s41380-019-0553-7

**Published:** 2019-11-29

**Authors:** Daisuke Koshiyama, Masaki Fukunaga, Naohiro Okada, Kentaro Morita, Kiyotaka Nemoto, Kaori Usui, Hidenaga Yamamori, Yuka Yasuda, Michiko Fujimoto, Noriko Kudo, Hirotsugu Azechi, Yoshiyuki Watanabe, Naoki Hashimoto, Hisashi Narita, Ichiro Kusumi, Kazutaka Ohi, Takamitsu Shimada, Yuzuru Kataoka, Maeri Yamamoto, Norio Ozaki, Go Okada, Yasumasa Okamoto, Kenichiro Harada, Koji Matsuo, Hidenori Yamasue, Osamu Abe, Ryuichiro Hashimoto, Tsutomu Takahashi, Tomoki Hori, Masahito Nakataki, Toshiaki Onitsuka, Laurena Holleran, Neda Jahanshad, Theo G. M. van Erp, Jessica Turner, Gary Donohoe, Paul M. Thompson, Kiyoto Kasai, Ryota Hashimoto, COCORO

**Affiliations:** 1 Department of Neuropsychiatry, Graduate School of Medicine, The University of Tokyo, Tokyo, Japan; 2 Division of Cerebral Integration, National Institute for Physiological Sciences, Aichi, Japan; 3 International Research Center for Neurointelligence (WPI-IRCN), UTIAS, The University of Tokyo, Tokyo, Japan; 4 Department of Psychiatry, Division of Clinical Medicine, Faculty of Medicine, University of Tsukuba, Tsukuba, Japan; 5 Department of Psychiatry, Osaka University Graduate School of Medicine, Suita, Japan; 6 Life Grow Brilliant Mental Clinic, Medical Corporation Foster, Osaka, Japan; 7 Department of Pathology of Mental Diseases, National Institute of Mental Health, National Center of Neurology and Psychiatry, Tokyo, Japan; 8 Molecular Research Center for Children’s Mental Development, United Graduate School of Child Development, Osaka University, Osaka, Japan; 9 Diagnostic and Interventional Radiology, Osaka University Graduate School of Medicine, Osaka, Japan; 10 Department of Psychiatry, Hokkaido University Graduate School of Medicine, Hokkaido, Japan; 11 Department of Neuropsychiatry, Kanazawa Medical University, Ishikawa, Japan; 12 Medical Research Institute, Kanazawa Medical University, Ishikawa, Japan; 13 Department of Psychiatry, Nagoya University, Graduate School of Medicine, Aichi, Japan; 14 Department of Psychiatry and Neurosciences, Graduate School of Biomedical and Health Sciences, Hiroshima University, Hiroshima, Japan; 15 Division of Neuropsychiatry, Department of Neuroscience, Yamaguchi University Graduate School of Medicine, Yamaguchi, Japan; 16 Department of Psychiatry, Faculty of Medicine, Saitama Medical University, Saitama, Japan; 17 Department of Psychiatry, Hamamatsu University School of Medicine, Shizuoka, Japan; 18 Department of Radiology, Graduate School of Medicine, The University of Tokyo, Tokyo, Japan; 19 Medical Institute of Developmental Disabilities Research, Showa University, Tokyo, Japan; 20 Department of Neuropsychiatry, University of Toyama Graduate School of Medicine and Pharmaceutical Sciences, Toyama, Japan; 21 Department of Psychiatry, Graduate School of Medicine, Kyoto University, Kyoto, Japan; 22 Department of Psychiatry, Tokushima University Hospital, Tokushima, Japan; 23 Department of Neuropsychiatry, Graduate School of Medical Sciences, Kyushu University, Fukuoka, Japan; 24 Center for Neuroimaging and Cognitive Genomics (NICOG), School of Psychology, National University of Ireland Galway, Galway, Ireland; 25 Imaging Genetics Center, Mark and Mary Stevens Neuroimaging and Informatics Institute, Keck School of Medicine, University of Southern California, Marina del Rey, CA USA; 26 Clinical Translational Neuroscience Laboratory, Department of Psychiatry and Human Behavior, University of California Irvine, Irvine, CA USA; 27 Psychology and Neuroscience, Georgia State University, Atlanta, GA USA

**Keywords:** Neuroscience, Psychiatric disorders, Diagnostic markers

## Abstract

Identifying both the commonalities and differences in brain structures among psychiatric disorders is important for understanding the pathophysiology. Recently, the ENIGMA-Schizophrenia DTI Working Group performed a large-scale meta-analysis and reported widespread white matter microstructural alterations in schizophrenia; however, no similar cross-disorder study has been carried out to date. Here, we conducted mega-analyses comparing white matter microstructural differences between healthy comparison subjects (HCS; *N* = 1506) and patients with schizophrenia (*N* = 696), bipolar disorder (*N* = 211), autism spectrum disorder (*N* = 126), or major depressive disorder (*N* = 398; total *N* = 2937 from 12 sites). In comparison with HCS, we found that schizophrenia, bipolar disorder, and autism spectrum disorder share similar white matter microstructural differences in the body of the corpus callosum; schizophrenia and bipolar disorder featured comparable changes in the limbic system, such as the fornix and cingulum. By comparison, alterations in tracts connecting neocortical areas, such as the uncinate fasciculus, were observed only in schizophrenia. No significant difference was found in major depressive disorder. In a direct comparison between schizophrenia and bipolar disorder, there were no significant differences. Significant differences between schizophrenia/bipolar disorder and major depressive disorder were found in the limbic system, which were similar to the differences in schizophrenia and bipolar disorder relative to HCS. While schizophrenia and bipolar disorder may have similar pathological characteristics, the biological characteristics of major depressive disorder may be close to those of HCS. Our findings provide insights into nosology and encourage further investigations of shared and unique pathophysiology of psychiatric disorders.

## Introduction

Classifying psychiatric disorders into distinct diagnoses remains challenging, although Kraepelin dissociated schizophrenia and bipolar disorder more than a century ago. While psychiatric disorders, such as schizophrenia, bipolar disorder, autism spectrum disorder, and major depressive disorder, display specific symptoms, many of their symptoms are shared by multiple disorders. Although studies show common genetic abnormalities across psychiatric disorders, the pathophysiological characteristics, such as regional brain abnormalities in gray matter or white matter across psychiatric disorders, are not fully understood [1–6]. Identifying commonalities or differences in pathophysiological characteristics across psychiatric disorders is important for the development of more precise and objective diagnoses and the effective development of new treatments.

Previous diffusion tensor imaging (DTI) studies have predominantly reported lower white matter fractional anisotropy (FA) in psychiatric disorders [7–32]. Lower FA in the prefrontal and temporal lobes and the fiber tracts connecting these regions have commonly been identified in individuals with schizophrenia [9–12]. Abnormalities in the cingulum and corpus callosum have been widely reported in individuals with bipolar disorder and autism spectrum disorder [13–32]. Some prior studies have also reported lower FA in the corpus callosum in individuals with major depressive disorder [7, 8]. These white matter alterations have the potential to differentiate pathophysiological characteristics between psychiatric disorders. However, the considerable heterogeneity both in the effect sizes and in the regional distribution of white matter alterations reported across studies has limited the conclusions that have been drawn to date. The heterogeneity between studies may be due to differences in analytic methodology, scanners, and study sample sizes [33].

To address variation in analysis methods and boost statistical power, the Enhancing Neuroimaging Genetics through Meta-Analysis (ENIGMA) Schizophrenia Working Group performed the first worldwide meta-analysis pooling schizophrenia-control effect sizes based on results from multiple sites generated using the same DTI, quality assurance, and statistical analysis methods. Thus, Kelly et al. performed a large-scale meta-analysis using the established ENIGMA-DTI protocol, which harmonized the processing of diffusion data from multiple sites (http://enigma.usc.edu/ongoing/dti-working-group/), and identified white matter microstructural alterations along with atlas-defined regions of interest (ROIs) in patients with schizophrenia [34–37]. This study identified widespread microstructural alterations, including the anterior corona radiata, corpus callosum, cingulum, and fornix, in individuals with schizophrenia. However, to the best of the authors’ knowledge, no similar large-scale meta- or mega-analysis study on white matter alterations across psychiatric disorders has been carried out.

The present study, a research project by a Japanese COCORO (Cognitive Genetics Collaborative Research Organization) consortium, conducts a large, multisite, cross-sectional investigation of white matter microstructural differences among healthy comparison subjects (HCS) and individuals with schizophrenia, bipolar disorder, autism spectrum disorder, and major depressive disorder by using mega-analytic methods similar to those of the study by Kelly et al., which has already shown differences in white matter microstructural alterations in the patients with schizophrenia compared with the HCS [34]. As a new line of work, we explored the common and distinct white matter microstructural alterations across four major psychiatric disorders. The first goal of this study is to replicate the findings by Kelly et al [34]. The second goal is to examine similarities and differences in white matter microstructure among HCS and individuals with schizophrenia, bipolar disorder, autism spectrum disorder, and major depressive disorder.

## Materials and methods

### Participants

A total of 2937 individuals assessed at 12 sites participated in the current cross-sectional COCORO cohort project. The cohort included data from 1506 HCS, 696 individuals with schizophrenia, 211 with bipolar disorder, 126 with autism spectrum disorder, and 398 with major depressive disorder (Table 1). Each participating site conducted magnetic resonance imaging (MRI) scanning and obtained DTI with one or more scanners and imaging protocols. A combination of one scanner and one imaging protocol was defined as one “protocol,” and 18 protocols were included in the current study. The data for HCS, schizophrenia, bipolar disorder, autism spectrum disorder, and major depressive disorder were obtained from 18, 15, 7, 4, and 7 protocols, respectively. HCS for the patients with four psychiatric diseases overlapped, and the HCS were not selected for each group of psychiatric diseases. Each sample had already contributed to prior neuroimaging studies [38–43]. Subject inclusion and exclusion criteria by site are described in Supplementary Method 1. Written informed consent, approved by each local institutional review board, was obtained from each subject prior to participation.

**Table 1:** Basic characteristics of the included protocols

Protocol name	Healthy comparison subjects					Individuals with schizophrenia				
				Age					Age	
	*N*	Male	Female	Mean	s.d.	*N*	Male	Female	Mean	s.d.
01. Osaka B	347	194	153	30.6	13.2	87	40	47	34.0	12.9
02. Osaka A	238	131	107	31.3	13.2	69	35	34	34.6	12.4
03. Kanazawa	113	72	41	33.9	9.9	111	44	67	39.9	12.5
04. Nagoya	124	77	47	36.5	9.8	53	29	24	43.0	9.9
05. Kyoto	75	46	29	28.6	9.4	52	24	28	36.4	9.0
06. Tokyo A	76	45	31	28.2	5.5	44	26	18	29.7	8.4
07. Toyama A	48	29	19	25.6	3.4	61	31	30	28.6	8.5
08. Hokkaido A	14	8	6	44.4	13.4	92	36	56	36.0	13.6
09. Hokkaido B	39	22	17	33.7	7.5	42	14	28	38.7	10.0
10. Tokyo D	51	17	34	39.2	7.9	16	10	6	29.4	9.1
11. Tokyo B	50	30	20	28.6	6.8	16	7	9	30.6	12.4
12. Tokushima	21	11	10	42.7	10.8	19	10	9	42.7	10.3
13. Tokyo C	27	10	17	38.0	9.5	8	5	3	37.9	4.2
14. Toyama B	11	4	7	26.8	3.6	15	10	5	28.6	9.1
15. Kyushu	6	4	2	35.8	11.8	11	9	2	54.6	9.7
Total	1240	700	540	32.0	11.6	696	330	366	36.0	12.2

	Healthy comparison subjects					Individuals with bipolar disorder				
01. Osaka B	347	194	153	30.6	13.2	5	4	1	41.8	17.2
02. Kanazawa	113	72	41	33.9	9.9	35	18	17	46.0	15.0
03. Nagoya	124	77	47	36.5	9.8	22	9	13	50.3	13.8
04. Yamaguchi	116	47	69	45.3	19.1	18	10	8	41.7	12.5
05. Hiroshima	79	41	38	52.4	14.4	39	16	23	52.2	13.6
06. Hokkaido A	14	8	6	44.4	13.4	77	41	36	44.8	14.6
07. Tokyo D	51	17	34	39.2	7.9	15	7	8	32.5	7.7
Total	844	456	388	36.7	15.0	211	105	106	45.7	14.6

	Healthy comparison subjects					Individuals with autism spectrum disorder				
01. Osaka B	347	194	153	30.6	13.2	26	16	10	27.2	8.9
02. Osaka A	238	131	107	31.3	13.2	12	8	4	24.8	10.1
03. Nagoya	124	77	47	36.5	9.8	13	13	0	31.3	9.4
04. Showa	71	58	13	27.5	6.2	75	65	10	30.1	6.6
Total	780	460	320	31.4	12.4	126	102	24	29.1	7.9

	Healthy comparison subjects					Individuals with major depressive disorder				
01. Osaka B	347	194	153	30.6	13.2	16	5	11	49.1	14.9
02. Osaka A	238	131	107	31.3	13.2	5	3	2	48.6	20.1
03. Hokkaido A	14	8	6	44.4	13.4	163	81	82	47.9	17.5
04. Yamaguchi	116	47	69	45.3	19.1	57	24	33	51.7	12.5
05. Kanazawa	113	72	41	33.9	9.9	43	27	16	43.2	13.8
06. Hiroshima	79	41	38	52.4	14.4	84	31	53	50.0	13.8
07. Tokyo D	51	17	34	39.2	7.9	30	12	18	37.1	11.0
Total	958	510	448	35.4	15.3	398	183	215	47.7	15.6

### Image processing

Multisite raw DTI data were pooled at the University of Tokyo where mega-analyses were performed. Detailed imaging parameters for each protocol are shown in Supplementary Method 2. Quality control included exclusion of duplicate participants across sites; visual inspection of the original T1-weighted images by two independent MRI researchers and exclusion of images with any abnormal findings (for example, large cerebellar cysts and cavum septum pellucidum); checking of the scan parameters for each DTI scan, and the exclusion of DTI data obtained with incorrect parameters; exclusion of DTI data that failed processing with FSL 5.0 (https://fsl.fmrib.ox.ac.uk/fsl) tract-based spatial statistics (TBSS). Furthermore, we excluded protocols with fewer than five participants in either diagnostic group in the mega-analysis for investigating group differences, and we excluded protocols with fewer than 15 participants in each diagnostic group in the mega-analysis for investigating correlations of the DTI indices with duration of illness or medication to minimize very small sample effects.

DTI image processing steps included head motion and eddy current correction using eddy_correct (FSL 5.0). Estimation of the following DTI indices were performed using dti_fit (FSL 5.0): FA, mean diffusivity (MD), axial diffusivity (AD), and radial diffusivity (RD). TBSS, using the ENIGMA-DTI template and JHU ROIs, was applied to extract local values of the DTI indices based on ENIGMA-DTI protocols (http://enigma.ini.usc.edu/protocols/dti-protocols/). FA is thought to indicate the underlying characteristics of white matter microstructure, such as the directionality of axonal fibers, diameter, density, and myelin sheath thickness. FA is derived from the degree of anisotropy of the following eigenvalues of the diffusion tensor: λ_1_, λ_2_, and λ_3_. The largest eigenvalue (λ_1_), i.e., the AD, is a possible marker for axonal injury. The average of the two smaller eigenvalues λ_2_ and λ_3_, i.e., the RD, is considered an indicator of myelin damage. The MD is the average of all three eigenvalues [44]. Finally, we obtained the DTI indices of 25 ROIs: corpus callosum, genu of the corpus callosum, body of the corpus callosum, splenium of the corpus callosum, cingulum (cingulate gyrus), cingulum (hippocampus), corona radiata, anterior corona radiata, posterior corona radiata, superior corona radiata, corticospinal tract, external capsule, fornix, fornix (crus)/stria terminalis, internal capsule, anterior limb of the internal capsule, posterior limb of the internal capsule, inferior fronto-occipital fasciculus, retrolenticular part of the internal capsule, posterior thalamic radiation, superior fronto-occipital fasciculus, superior longitudinal fasciculus, sagittal stratum, uncinate fasciculus, and average of the full skeleton’s FA (MD, AD, and RD) [34].

### Statistical analysis

All linear regression analyses were conducted using SPSS version 23.0.0.0 (IBM Corp., Armonk, NY). Wolfers et al. noted that group-level differences disguised biological heterogeneity and interindividual differences among patients [45]. To address this issue, we compared the variability of the DTI indices between the HCS group and the schizophrenia, bipolar disorder, autism spectrum disorder, or major depressive disorder groups, similar to Brugger et al. [46] (Supplementary Method 3).

For comparison of the DTI indices, we calculated Cohen’s *d* effect sizes in each of the 25 ROIs for FA, MD, AD, and RD (four DTI indices) differences in HCS versus individuals with schizophrenia, bipolar disorder, autism spectrum disorder, and major depressive disorder after including age, sex, age × sex, age^2^ and age^2^ × sex as covariates for each cohort same as previous studies [34, 47]. Next, we performed mega-analyses across cohorts with Cohen’s *d* effect sizes using Metasoft software (http://genetics.cs.ucla.edu/meta) similar to Kelly et al. [34]. In addition, we conducted direct comparisons among the patient groups in the same way to identify the common and unique white matter microstructure alterations across the diseases (Supplementary Table 1).

We evaluated the effect of duration of illness on the four DTI indices. We calculated the *β* coefficients for associations of DTI metrics with duration of illness, considering sex as a covariate in regression analyses in patients with schizophrenia, bipolar disorder, autism spectrum disorder, and major depressive disorder for each cohort. For the individuals with autism spectrum disorder, we employed age instead of the duration of illness. Then, we performed mega-analyses across cohorts with the *β* coefficients using the metacor package in R (https://cran.r-project.org/web/packages/metacor/), which is suitable for meta-analyses with the *β* coefficients.

Furthermore, we evaluated medication effects on all four DTI indices. We calculated *β* coefficients of the chlorpromazine equivalent dose, considering age, sex, age × sex, age^2^, and age^2^ × sex as covariates in individuals with schizophrenia for each sample. We calculated *β* coefficients of lithium dose, considering age, sex, age × sex, age^2^, and age^2^ × sex as covariates in patients with bipolar disorder for each sample. We calculated *β* coefficients of the imipramine equivalent dose, considering age, sex, age × sex, age^2^, and age^2^ × sex as covariates in individuals with major depressive disorder for each sample. Next, we performed mega-analyses across samples with the *β* coefficients using the metacor package in R (https://cran.r-project.org/web/packages/metacor/). The significance level was set at a Bonferroni corrected *p* value of 0.002 (0.05/25). We conducted a power analysis to estimate the sample sizes required to detect an effect size with a previous study at a power of 0.80 and a one-tailed significance level of 0.002 (0.05/25), with G*Power version 3.1.9.2 [34, 48].

## Results

### Variability ratio of the DTI indices

The number of DTI indices that the variability ratio was significantly higher compared with HCS was larger in the order of schizophrenia, major depressive disorder, bipolar disorder, and autism spectrum disorder (Supplementary Tables 2–17). However, *I*^*2*^ was in the upper 90% for almost all DTI indices in all groups, and the heterogeneity in each protocol was large; it was difficult to interpret the results.

### DTI differences between healthy comparison subjects and patients with schizophrenia

**Fig. 1:**
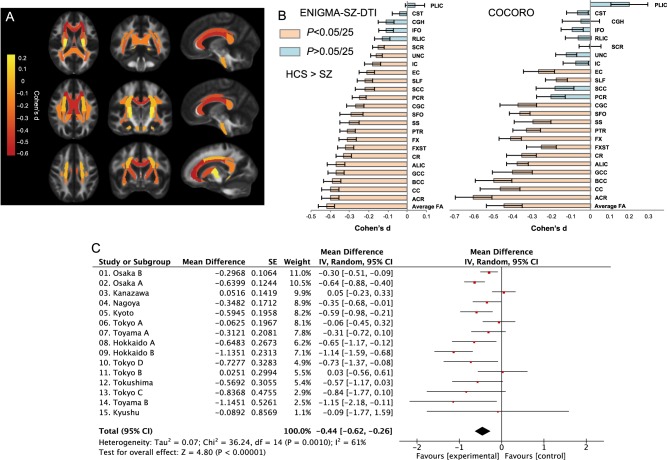
Differences in fractional anisotropy between the patients with schizophrenia (SZ) and healthy comparison subjects (HCS). **a** Fractional anisotropy (FA) differences between the patients with schizophrenia and healthy comparison subjects (HCS) for 25 white matter regions representing major fasciculi. The gradient bar indicates Cohen’s *d* effect sizes after mega-analysis. **b** The figure on the right, representing our results from COCORO, shows Cohen’s *d* effect sizes after mega-analysis across 15 cohorts for FA differences in the patients with schizophrenia (*N* = 696) versus HCS (*N* = 1240), after including age, sex, age × sex, age^2^, and age^2^ × sex as covariates. Error bars represent 95% confidence intervals. Regions with significant differences after adjusting for multiple regions tested [*p* < 0.002 (0.05/25)] are highlighted in orange. The figure on the left represents the results from the ENIGMA-Schizophrenia DTI consortium [29 cohorts for FA differences in patients with schizophrenia (*N* = 1963) versus HCS (*N* = 2359)]. The average FA represents the average FA of the full skeleton. We generally replicated the results of ENIGMA-Schizophrenia DTI. **c** Forest plot of effect sizes for 15 cohorts for average FA differences in the patients with schizophrenia versus HCS. COCORO Cognitive Genetics Collaborative Research Organization, ENIGMA-SZ-DTI Enhancing Neuroimaging Genetics through Meta-Analysis consortium-Schizophrenia diffusion tensor imaging, CC corpus callosum, GCC genu of the corpus callosum, BCC body of the corpus callosum, SCC splenium of the corpus callosum, CGC cingulum (cingulate gyrus), CGH cingulum (hippocampus), CR corona radiata, ACR anterior corona radiata, PCR posterior corona radiata, SCR superior corona radiata, CST corticospinal tract, EC external capsule, FX fornix, FX/ST fornix (crus)/stria terminalis, IC internal capsule, ALIC anterior limb of the internal capsule, PLIC posterior limb of the internal capsule, IFO inferior fronto-occipital fasciculus, RLIC retrolenticular part of the internal capsule, PTR posterior thalamic radiation, SFO superior fronto-occipital fasciculus, SLF superior longitudinal fasciculus, SS sagittal stratum, UNC uncinate fasciculus

The results comparing DTI indices between HCS and individuals with schizophrenia are shown in Table 2, Fig. 1, Supplementary Tables 18–21, and Supplementary Figs. 1–5. Of the 25 regions, 15 regions showed significantly lower FA in patients with schizophrenia than in HCS. The largest effect size was observed for lower FA in individuals with schizophrenia in the anterior corona radiata, followed by the body of the corpus callosum, corpus callosum, whole-brain white matter skeleton (i.e., average FA), fornix, and cingulate gyrus. Significant FA reductions were also found in nine additional ROIs in patients with schizophrenia. We found significantly higher MD in the fornix, body of the corpus callosum, whole-brain white matter skeleton (i.e., average MD), corpus callosum, and uncinate fasciculus in individuals with schizophrenia than in HCS. Significantly higher MD was also found in seven additional ROIs in patients with schizophrenia than in HCS. We found significantly higher AD in the fornix, uncinate fasciculus, superior corona radiata, and posterior corona radiata in individuals with schizophrenia than in HCS. We found significantly higher RD in the fornix, anterior corona radiata, whole-brain white matter skeleton (i.e., average RD), corpus callosum, body of the corpus callosum, cingulate gyrus, and uncinate fasciculus in patients with schizophrenia than in HCS. Significantly higher RD was also found in 11 additional ROIs in individuals with schizophrenia than in HCS.

**Table 2:** Brain region specificity of white matter microstructural alterations in psychiatric disorders

Limbic system–limbic system	Limbic system/spine/brain stem–cortex	Right cortex–left cortex	Cortex–cortex (ipsilateral side)
Schizophrenia			
Cingulum (cingulate gyrus; FA, RD)	Anterior corona radiata (FA, MD, RD)	Body of the corpus callosum (FA, MD, RD)	Superior fronto-occipital fasciculus (FA, MD, RD)
External capsule (FA, RD)	Anterior limb of the internal capsule (FA, RD)	Corpus callosum (FA, MD, RD)	Superior longitudinal fasciculus (FA, MD, RD)
Fornix (column and body of the fornix; FA, MD, AD, RD)	Corona radiata (FA, MD, RD)	Genu of the corpus callosum (FA, MD, RD)	Uncinate fasciculus (MD, AD, RD)
Fornix (crus)/Stria terminalis (FA, RD)	Posterior corona radiata (MD, AD, RD)		
	Posterior thalamic radiation (FA, RD)		
	Superior corona radiata (MD, AD, RD)		
	Sagittal stratum (FA, RD)		
Bipolar disorder			
Cingulum (cingulate gyrus; FA)	Posterior limb of the internal capsule (AD)	Body of the corpus callosum (MD, RD)	
Fornix (column and body of the fornix; MD, AD, RD)			
Autism spectrum disorder			
		Body of the corpus callosum (FA)	
Major depressive disorder			

ROIs, which showed significant differences between patients with psychiatric disorders and healthy comparison subjects after adjusting age, sex, age × sex, age^2^, and age^2^ × sex as covariates, were listed. The ROIs were classified according to the brain regions that they connect

*FA* fractional anisotropy, *MD* mean diffusivity, *AD* axial diffusivity, *RD* radial diffusivity, *ROI* region of interest

### DTI differences between healthy comparison subjects and patients with bipolar disorder, autism spectrum disorder, and major depressive disorder

**Fig. 2:**
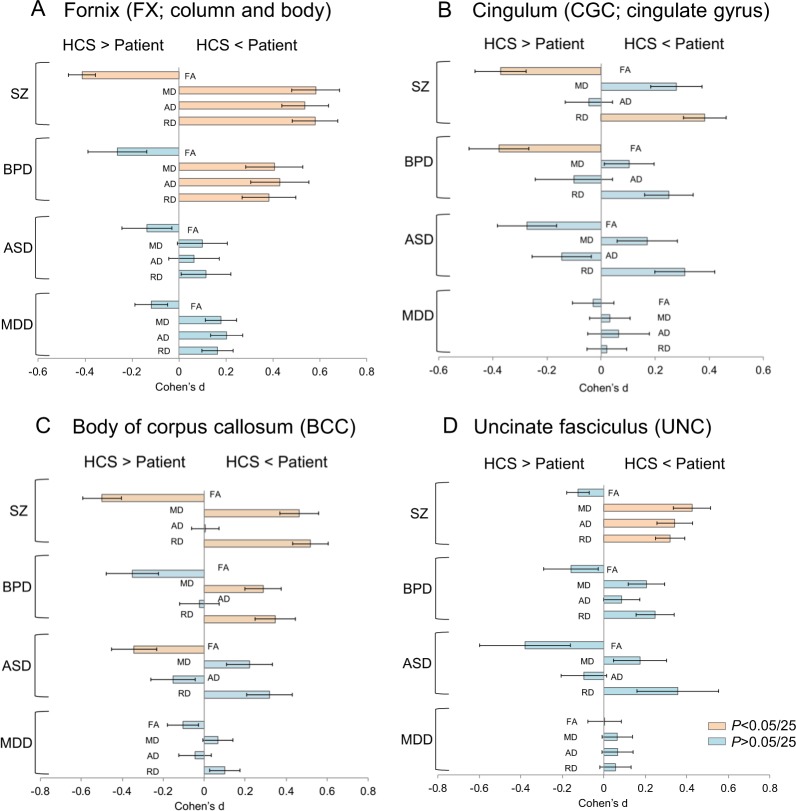
Effect sizes of the differences in DTI indices between patients with psychiatric disorders and healthy comparison subjects in each white matter region for the main findings. Significant differences in regions after adjusting for multiple regions tested [*p* < 0.002 (0.05/25)] are highlighted in orange. DTI diffusion tensor imaging, HCS healthy comparison subjects, SZ schizophrenia, BPD bipolar disorder, ASD autism spectrum disorder, MDD major depressive disorder, FA fractional anisotropy, MD mean diffusivity, AD axial diffusivity, RD radial diffusivity

**Fig. 3:**
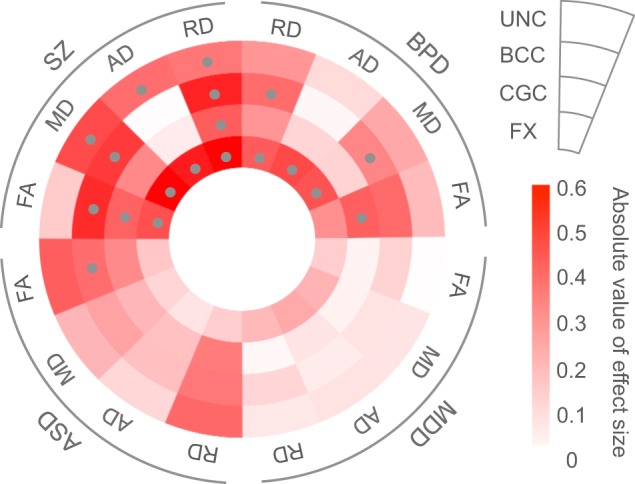
White matter microstructural alterations across psychiatric disorders. Color scales indicate the absolute value of Cohen’s *d* effect size of the DTI indices (FA, MD, AD, and RD) between each patient population (schizophrenia, bipolar disorder, autism spectrum disorder, and major depressive disorder) and healthy comparison subjects in each region of interest (UNC, BCC, CGC, and FX). Gray dots indicate statistical significance. The patients with schizophrenia, bipolar disorder, and autism spectrum disorder have common white matter alterations in the body of the corpus callosum; the patients with schizophrenia and bipolar disorder have common white matter alterations in the limbic system, such as the fornix and the cingulum; microstructural alterations in white matter regions that connect neocortical areas, such as uncinate fasciculus, were specific to patients with schizophrenia. DTI diffusion tensor imaging, SZ schizophrenia, BPD bipolar disorder, MDD major depressive disorder, ASD autism spectrum disorder, FA fractional anisotropy, MD mean diffusivity, AD axial diffusivity, RD radial diffusivity, UNC uncinate fasciculus, BCC body of the corpus callosum, CGC cingulum (cingulate gyrus), FX fornix

The results comparing DTI indices between HCS and individuals with bipolar disorder, autism spectrum disorder, or major depressive disorder are shown in Table 2, Figs. 2 and 3, Supplementary Tables 22–33, and Supplementary Figs. 4–17. We found significantly lower FA in the cingulate gyrus, higher MD in the fornix and body of the corpus callosum, higher AD in the fornix and posterior limb of the internal capsule, and lower RD in the fornix, body of the corpus callosum, and the whole-brain white matter skeleton (i.e., average RD) in patients with bipolar disorder than in HCS. Significantly lower FA was found in the body of the corpus callosum in individuals with autism spectrum disorder than in HCS. There were no significant differences in DTI indices in patients with major depressive disorder compared with HCS.

### DTI differences among the patient groups

The results comparing DTI indices among the patient groups are shown in Supplementary Table 34–57. We found significantly lower FA in the fornix, higher MD in the fornix, higher AD in the fornix and superior corona radiata, and higher RD in the fornix in individuals with schizophrenia than in those with autism spectrum disorder. We found significantly lower FA in the anterior limb of internal capsule, corpus callosum, and superior fronto-occipital fasciculus, higher MD in the body of corpus callosum, corpus callosum, cingulum (cingulate gyrus), and splenium of corpus callosum, and higher RD in body of corpus callosum, corpus callosum, cingulum (cingulate gyrus), and splenium of corpus callosum in individuals with schizophrenia than in those with major depressive disorder. Significantly higher MD and RD were found in the fornix in the individuals with bipolar disorder than in those with major depressive disorder. There were no significant differences in DTI indices between patients with schizophrenia and those with bipolar disorder, between individuals with bipolar disorder and those with autism spectrum disorder, and between individuals with autism spectrum disorder and those with major depressive disorder.

### Relationships with duration of illness

The relationships between duration of illness and DTI indices are shown in Supplementary Tables 58–73. The duration of illness showed significant negative correlations with FA in the anterior corona radiata, posterior thalamic radiation, corona radiata, fornix, and body of the corpus callosum, as well as in ten additional regions, in individuals with schizophrenia. The duration of illness showed significant negative correlations with FA in the anterior corona radiata, genu of the corpus callosum, whole-brain white matter skeleton (i.e., average FA), and fornix in patients with bipolar disorder. The duration of illness (age) showed significant negative correlations with FA in the posterior thalamic radiation, genu of the corpus callosum, retrolenticular part of the internal capsule, corona radiata, posterior corona radiata, and internal capsule in individuals with autism spectrum disorder. The duration of illness showed significant negative correlations with FA in the fornix, fornix (crus)/stria terminalis, superior fronto-occipital fasciculus, and whole-brain white matter skeleton (i.e., average FA) in the individuals with major depressive disorder. The relationships between the duration of illness and MD, AD, and RD were mostly consistent with those of FA in each of the patient groups.

### Relationships with medications

The relationships between medications and DTI indices are shown in Supplementary Tables 74–85. There were no significant relationships between DTI indices and chlorpromazine equivalent dose, lithium dose, or imipramine equivalent dose in individuals with schizophrenia, bipolar disorder, or major depressive disorder.

## Discussion

Our study largely replicates the findings of the ENIGMA-Schizophrenia Working Group (Fig. 1) [34]. For example, the case-control effect size for average FA was *d* = –0.44 in our study compared with *d* = –0.42 in the ENGIMA meta-analysis [34]. The markedly lower FA in the anterior corona radiata, corpus callosum, fornix, and cingulate gyrus regions were well replicated in individuals with schizophrenia. We replicated the significantly higher MD in the anterior corona radiata, corpus callosum, and fornix regions, and furthermore, we are the first to report significantly higher MD in the uncinate fasciculus. Although Kelly et al. reported significantly higher AD only in the fornix, we have shown significantly higher AD in three regions, such as the uncinate fasciculus, in addition to fornix [34]. In this study, significantly higher RD were found in the anterior corona radiata, corpus callosum, fornix, cingulate gyrus, and uncinate fasciculus regions, as in the study by Kelly et al., and we are the first to report significantly higher RD in the fornix and cingulate gyrus [34]. Prior studies have reported lower FA in the corpus callosum, fornix, and cingulum in individuals with bipolar disorder, higher AD and RD in the fornix in patients with bipolar disorder, and lower FA, coupled with MD, AD, and RD in the corpus callosum in individuals with autism spectrum disorders; these results are consistent with our findings [13–22, 24, 26, 28–30]. Furthermore, Chang et al. found lower FA in the corpus callosum, fornix, and cingulum both in 135 individuals with schizophrenia and 86 with bipolar disorder than in 156 healthy controls, but not in 108 with MDD; these results are consistent with our findings [49]. The corpus callosum is an extensive bilateral hemisphere relay center and connects extensive networks subserving memory, attention, language, intelligence, and emotional states; the fornix is located at the center of the limbic system and contains pathways that connect brain regions important for memory performance, emotion regulation, and reward processing, such as the hippocampus, thalamus, and nucleus accumbens [50–52]. The cingulum bundles, which are the primary intrahemispheric association pathways for the medial cingulate cortex and temporal lobe structures, are located above the corpus callosum. The cingulum bundles connect the anterior cingulate to the posterior cingulate cortex curve along with the splenium of the corpus callosum, and they connect to the hippocampus [24]. Our ROI for the cingulum was divided into two regions with the cingulum around the cingulate gyrus and the cingulum around the hippocampus. The ROI that we found a difference between the patients and HCS in this study was the cingulum around the cingulate gyrus. We did not find microstructural alterations in the uncinate fasciculus in psychiatric disorders other than schizophrenia; however, a prior meta-analysis reported lower FA in the uncinate fasciculus in 117 individuals with autism spectrum disorder compared with 125 HCS in six previously reported studies [53]. This inconsistent result may be due to the difference in mean age of participants, sample size or analysis method. Two hundred forty-two individuals (mean age ranging from 5 to 28.5 years among the six studies) were included in the study by Aoki et al., and 906 individuals [780 HCS (mean age was 31.4) and 126 individuals with autism spectrum disorder (mean age was 29.1)] were included in the current study. Aoki et al. conducted a meta-analysis based on six published studies, while we conducted a mega-analysis based on jointly analyzed data [53].

In comparison with HCS, we found that patients with schizophrenia, bipolar disorder, or autism spectrum disorder have common white matter alterations in the body of the corpus callosum (Fig. 3). Previous genetic studies have shown phenotypic and genetic overlap across psychiatric disorders, for example, between schizophrenia and bipolar disorder, between autism spectrum disorders and schizophrenia, and between autism spectrum disorders and bipolar disorder [1–6]. Our findings of common white matter alterations among individuals with schizophrenia, bipolar disorder, and autism spectrum disorders may in part reflect genetic overlap. Furthermore, patients with schizophrenia and bipolar disorder have common alterations in white matter microstructure in the limbic system, i.e., the fornix and cingulum; microstructural alterations in white matter regions that connect neocortical areas, such as the uncinate fasciculus, were specific to individuals with schizophrenia. Lower volumes in neocortical regions, such as the frontal and temporal gyri, which are repeatedly reported in patients with schizophrenia, may be associated with the specificity of the microstructural alterations in white matter regions connecting these neocortical regions in individuals with schizophrenia [54–56]. It is known that not only individuals with schizophrenia but also those with bipolar disorder show cognitive impairment, albeit less severe in the latter case [57–59]. We speculate that the common cognitive dysfunctions across psychotic disorders are potentially associated with the common alterations in the limbic system and corpus callosum across psychiatric disorders, which have important roles in memory performance. However, specific alterations in white matter regions connecting neocortical areas may be related to more severe cognitive decline in patients with schizophrenia. Our hypothesis is supported by previous findings that white matter alterations in the anterior corona radiata and corpus callosum are associated with social dysfunction in individuals with schizophrenia and by current our results, which have shown significant correlations between the duration of illness and alterations in white matter tracts connecting neocortical regions, including the anterior corona radiata and uncinate fasciculus, in patients with schizophrenia [40]. These findings may suggest that the gradual progressive impairment of white matter microstructure may be associated with the gradual functional decline in individuals with schizophrenia [60]. The lack of white matter alterations in the patients with major depressive disorder may reflect different pathological characteristics between the patients with major depressive disorder and those with other psychiatric disorders. In addition, only one region of white matter alteration was found in individuals with autism spectrum disorders, and they also have the potential to have different pathological characteristics with other psychiatric disorders. Individuals with major depressive disorder and autism spectrum disorders may be a heterogeneous population, and some of them may have biological characteristics similar to those of HCS.

In a direct comparison between individuals with schizophrenia and those with bipolar disorder, there were no significant white matter microstructural differences. Significant differences in the limbic system were found between schizophrenia and major depressive disorder, between schizophrenia and autism spectrum disorders, and between bipolar disorder and major depressive disorder. Furthermore, those regions were similar to the regions where the differences were observed between individuals with schizophrenia and HCS and between bipolar disorder and HCS. Because the biological characteristics of major depressive disorder and autism spectrum disorder are close to those of HCS, we may have found similar patterns between them. The Diagnostic and Statistical Manual of Mental Disorders, fifth edition (DSM-5), which was published in 2013, originally aimed to use biological indicators for the diagnosis of psychiatric disorders; however, this goal had not been reached, and therefore, the DSM-5 still relies on symptomatic diagnosis [61]. Our results may support the diagnosis of bipolar disorders being separated from those of depressive disorders in DSM-5. Our findings of common or unique white matter alteration across psychiatric disorders may contribute to the further development of new diagnostic methods when considered with other intermediate phenotypes in psychiatric disorders.

Relationships between duration of illness and white matter indices were widespread in patients with schizophrenia and present in those with bipolar disorder, autism spectrum disorder, and major depressive disorder. Our findings of those significant relationships in white matter tracts, such as the anterior corona radiata and corpus callosum in individuals with schizophrenia, were similar to those reported by the ENIGMA-Schizophrenia meta-analysis [34]. Because multiple DTI indices in the regions, such as anterior corona radiata, body of the corpus callosum, fornix, and uncinate fasciculus, were correlated with duration of illness in individuals with schizophrenia and bipolar disorder, progressive pathological change may cause more severe axonal injury and myelin damage in those regions in those patients. In contrast, there were no significant associations between medication doses and the DTI indices in the patients with schizophrenia, bipolar disorder, or major depressive disorder.

Some limitations of our study must be noted. First, the effects of MRI scanner differences on our findings cannot be completely ruled out; however, we minimized this effect using the mega-analysis method. It may be desirable for classification analyses to be performed using the DTI indices among the patient groups; however, there are methodological limitations due to the large effect of MRI scanner differences. Second, although the medication effect on the DTI indices cannot be completely ruled out, we found no significant medication dose-related effects on the DTI indices using regression analysis. Third, associations between symptom severity and effect sizes of DTI indices were not investigated in this study. Investigating relationships of DTI indices with cognitive function, depressive state, manic state, and psychotic syndrome will be fruitful for understanding common or unique pathologies across psychotic disorders in the future. Fourth, genetic or environmental effects on the DTI indices were not investigated in the current study. It is known that lower birth weight among individuals who later develop schizophrenia than among HCS, and a previous study has shown that birth weight influences the white matter volume in patients with schizophrenia [62–64]. Future research investigating the association between birth weight and DTI indices may be useful to show the effects of gene and environment interaction on white matter alteration in patients with psychotic disorders.

In conclusion, we have shown white matter microstructural alterations across psychiatric disorders. The widespread white matter microstructural alterations in limbic and cortical regions in schizophrenia reported by Kelly et al. were successfully replicated in the present study [34]. Furthermore, in comparison with HCS, we revealed that patients with schizophrenia, bipolar disorder and autism spectrum disorder have shared common white matter alterations in the body of the corpus callosum; individuals with schizophrenia and bipolar disorder have common white matter alterations in the limbic system, including in the fornix and cingulum; and microstructural alterations in white matter regions that connect neocortical areas, such as uncinate fasciculus, were specific to schizophrenia; there was no significant difference in major depressive disorder. In a direct comparison between schizophrenia and bipolar disorder, there were no significant differences; significant differences between schizophrenia/bipolar disorder and major depressive disorder were found in the limbic system, which were similar to the difference between schizophrenia/bipolar disorder and HCS. Our findings suggest that pathological characteristics may be similar between schizophrenia and bipolar disorder; however, the biological characteristics of major depressive disorder may be close to those of HCS. Our findings of common or unique white matter alteration across psychiatric disorders provide insights into nosology and encourage the investigation of pathophysiology for psychiatric disorders.

## Supplementary information

Supplementary text

## References

[1] Cross-Disorder Group of the Psychiatric Genomics Consortium. (2013). Identification of risk loci with shared effects on five major psychiatric disorders: a genome-wide analysis. Lancet.

[2] Purcell SM, Wray NR, Stone JL, Visscher PM, O’Donovan MC, Sullivan PF (2009). Common polygenic variation contributes to risk of schizophrenia and bipolar disorder. Nature.

[3] Rapoport J, Chavez A, Greenstein D, Addington A, Gogtay N (2009). Autism spectrum disorders and childhood-onset schizophrenia: clinical and biological contributions to a relation revisited. J Am Acad Child Adolesc Psychiatry.

[4] Sullivan PF, Magnusson C, Reichenberg A, Boman M, Dalman C, Davidson M (2012). Family history of schizophrenia and bipolar disorder as risk factors for autism. Arch Gen Psychiatry.

[5] Autism Spectrum Disorders Working Group of The Psychiatric Genomics Consortium. (2017). Meta-analysis of GWAS of over 16,000 individuals with autism spectrum disorder highlights a novel locus at 10q24.32 and a significant overlap with schizophrenia. Mol Autism.

[6] Crespi B, Stead P, Elliot M (2010). Evolution in health and medicine Sackler colloquium: comparative genomics of autism and schizophrenia. Proc Natl Acad Sci USA.

[7] Chen G, Guo Y, Zhu H, Kuang W, Bi F, Ai H (2017). Intrinsic disruption of white matter microarchitecture in first-episode, drug-naive major depressive disorder: a voxel-based meta-analysis of diffusion tensor imaging. Prog Neuropsychopharmacol Biol Psychiatry.

[8] Jiang J, Zhao YJ, Hu XY, Du MY, Chen ZQ, Wu M (2017). Microstructural brain abnormalities in medication-free patients with major depressive disorder: a systematic review and meta-analysis of diffusion tensor imaging. J Psychiatry Neurosci.

[9] Ellison-Wright I, Bullmore E (2009). Meta-analysis of diffusion tensor imaging studies in schizophrenia. Schizophr Res.

[10] Pettersson-Yeo W, Allen P, Benetti S, McGuire P, Mechelli A (2011). Dysconnectivity in schizophrenia: where are we now?. Neurosci Biobehav Rev.

[11] Kubicki M, McCarley R, Westin CF, Park HJ, Maier S, Kikinis R (2007). A review of diffusion tensor imaging studies in schizophrenia. J Psychiatr Res.

[12] Zalesky A, Fornito A, Seal ML, Cocchi L, Westin CF, Bullmore ET (2011). Disrupted axonal fiber connectivity in schizophrenia. Biol Psychiatry.

[13] Kurumaji A, Itasaka M, Uezato A, Takiguchi K, Jitoku D, Hobo M (2017). A distinctive abnormality of diffusion tensor imaging parameters in the fornix of patients with bipolar II disorder. Psychiatry Res Neuroimaging.

[14] Knochel C, Schmied C, Linden DE, Stablein M, Prvulovic D, de Ad CL (2016). White matter abnormalities in the fornix are linked to cognitive performance in SZ but not in BD disorder: an exploratory analysis with DTI deterministic tractography. J Affect Disord.

[15] Barysheva M, Jahanshad N, Foland-Ross L, Altshuler LL, Thompson PM (2013). White matter microstructural abnormalities in bipolar disorder: a whole brain diffusion tensor imaging study. Neuroimage Clin.

[16] Barnea-Goraly N, Chang KD, Karchemskiy A, Howe ME, Reiss AL (2009). Limbic and corpus callosum aberrations in adolescents with bipolar disorder: a tract-based spatial statistics analysis. Biol Psychiatry.

[17] Oertel-Knochel V, Reinke B, Alves G, Jurcoane A, Wenzler S, Prvulovic D (2014). Frontal white matter alterations are associated with executive cognitive function in euthymic bipolar patients. J Affect Disord.

[18] Vederine FE, Wessa M, Leboyer M, Houenou J (2011). A meta-analysis of whole-brain diffusion tensor imaging studies in bipolar disorder. Prog Neuropsychopharmacol Biol Psychiatry.

[19] Nortje G, Stein DJ, Radua J, Mataix-Cols D, Horn N (2013). Systematic review and voxel-based meta-analysis of diffusion tensor imaging studies in bipolar disorder. J Affect Disord.

[20] Di X, Azeez A, Li X, Haque E, Biswal BB (2018). Disrupted focal white matter integrity in autism spectrum disorder: a voxel-based meta-analysis of diffusion tensor imaging studies. Prog Neuropsychopharmacol Biol Psychiatry.

[21] Aoki Y, Yoncheva YN, Chen B, Nath T, Sharp D, Lazar M (2017). Association of white matter structure with autism spectrum disorder and attention-deficit/hyperactivity disorder. JAMA Psychiatry.

[22] Nickel K, Tebartz van Elst L, Perlov E, Endres D, Muller GT, Riedel A (2017). Altered white matter integrity in adults with autism spectrum disorder and an IQ >100: a diffusion tensor imaging study. Acta Psychiatr Scand.

[23] Ameis SH, Fan J, Rockel C, Soorya L, Wang AT, Anagnostou E (2013). Altered cingulum bundle microstructure in autism spectrum disorder. Acta Neuropsychiatr.

[24] Travers BG, Adluru N, Ennis C, Tromp do PM, Destiche D, Doran S (2012). Diffusion tensor imaging in autism spectrum disorder: a review. Autism Res.

[25] Barnea-Goraly N, Kwon H, Menon V, Eliez S, Lotspeich L, Reiss AL (2004). White matter structure in autism: preliminary evidence from diffusion tensor imaging. Biol Psychiatry.

[26] Jou RJ, Jackowski AP, Papademetris X, Rajeevan N, Staib LH, Volkmar FR (2011). Diffusion tensor imaging in autism spectrum disorders: preliminary evidence of abnormal neural connectivity. Aust N Z J Psychiatry.

[27] Jou RJ, Mateljevic N, Kaiser MD, Sugrue DR, Volkmar FR, Pelphrey KA (2011). Structural neural phenotype of autism: preliminary evidence from a diffusion tensor imaging study using tract-based spatial statistics. AJNR Am J Neuroradiol.

[28] Kumar A, Sundaram SK, Sivaswamy L, Behen ME, Makki MI, Ager J (2010). Alterations in frontal lobe tracts and corpus callosum in young children with autism spectrum disorder. Cereb Cortex.

[29] Lee JE, Chung MK, Lazar M, DuBray MB, Kim J, Bigler ED (2009). A study of diffusion tensor imaging by tissue-specific, smoothing-compensated voxel-based analysis. Neuroimage.

[30] Noriuchi M, Kikuchi Y, Yoshiura T, Kira R, Shigeto H, Hara T (2010). Altered white matter fractional anisotropy and social impairment in children with autism spectrum disorder. Brain Res.

[31] Pardini M, Garaci FG, Bonzano L, Roccatagliata L, Palmieri MG, Pompili E (2009). White matter reduced streamline coherence in young men with autism and mental retardation. Eur J Neurol.

[32] Thakkar KN, Polli FE, Joseph RM, Tuch DS, Hadjikhani N, Barton JJ (2008). Response monitoring, repetitive behaviour and anterior cingulate abnormalities in autism spectrum disorders (ASD). Brain.

[33] Melicher T, Horacek J, Hlinka J, Spaniel F, Tintera J, Ibrahim I (2015). White matter changes in first episode psychosis and their relation to the size of sample studied: a DTI study. Schizophr Res.

[34] Kelly S, Jahanshad N, Zalesky A, Kochunov P, Agartz I, Alloza C, et al. Widespread white matter microstructural differences in schizophrenia across 4322 individuals: results from the ENIGMA Schizophrenia DTI Working Group. Mol Psychiatry. 2018;23:1261–9. 10.1038/mp.2017.170 PMC5984078 29038599

[35] Jahanshad N, Kochunov PV, Sprooten E, Mandl RC, Nichols TE, Almasy L (2013). Multi-site genetic analysis of diffusion images and voxelwise heritability analysis: a pilot project of the ENIGMA-DTI working group. Neuroimage.

[36] Kochunov P, Jahanshad N, Sprooten E, Nichols TE, Mandl RC, Almasy L (2014). Multi-site study of additive genetic effects on fractional anisotropy of cerebral white matter: comparing meta and megaanalytical approaches for data pooling. Neuroimage.

[37] Kochunov P, Jahanshad N, Marcus D, Winkler A, Sprooten E, Nichols TE (2015). Heritability of fractional anisotropy in human white matter: a comparison of Human Connectome Project and ENIGMA-DTI data. Neuroimage.

[38] Morita K, Miura K, Fujimoto M, Shishido E, Shiino T, Takahashi J, et al. Abnormalities of eye movement are associated with work hours in schizophrenia. Schizophr Res. 2018;202:420–2. 10.1016/j.schres.2018.06.064 30017461

[39] Koshiyama D, Fukunaga M, Okada N, Yamashita F, Yamamori H, Yasuda Y (2018). Role of subcortical structures on cognitive and social function in schizophrenia. Sci Rep.

[40] Koshiyama D, Fukunaga M, Okada N, Morita K, Nemoto K, Yamashita F, et al. Role of frontal white matter and corpus callosum on social function in schizophrenia. Schizophr Res. 2018;202:180–7. 10.1016/j.schres.2018.07.009 30005932

[41] Koshiyama D, Fukunaga M, Okada N, Yamashita F, Yamamori H, Yasuda Y (2018). Subcortical association with memory performance in schizophrenia: a structural magnetic resonance imaging study. Transl Psychiatry.

[42] Okada N, Fukunaga M, Yamashita F, Koshiyama D, Yamamori H, Ohi K (2016). Abnormal asymmetries in subcortical brain volume in schizophrenia. Mol Psychiatry.

[43] Morita K, Miura K, Fujimoto M, Yamamori H, Yasuda Y, Iwase M (2017). Eye movement as a biomarker of schizophrenia: Using an integrated eye movement score. Psychiatry Clin Neurosci.

[44] Fan S, van den Heuvel OA, Cath DC, van der Werf YD, de Wit SJ, de Vries FE (2015). Mild white matter changes in un-medicated obsessive-compulsive disorder patients and their unaffected siblings. Front Neurosci.

[45] Wolfers T, Doan NT, Kaufmann T, Alnaes D, Moberget T, Agartz I (2018). Mapping the heterogeneous phenotype of schizophrenia and bipolar disorder using normative models. JAMA Psychiatry.

[46] Brugger SP, Howes OD (2017). Heterogeneity and homogeneity of regional brain structure in schizophrenia: a meta-analysis. JAMA Psychiatry.

[47] Kochunov P, Williamson DE, Lancaster J, Fox P, Cornell J, Blangero J (2012). Fractional anisotropy of water diffusion in cerebral white matter across the lifespan. Neurobiol Aging.

[48] Faul F, Erdfelder E, Lang AG, Buchner A (2007). G*Power 3: a flexible statistical power analysis program for the social, behavioral, and biomedical sciences. Behav Res Methods.

[49] Chang M, Womer FY, Edmiston EK, Bai C, Zhou Q, Jiang X (2018). Neurobiological commonalities and distinctions among three major psychiatric diagnostic categories: a structural MRI study. Schizophr Bull.

[50] Lovblad KO, Schaller K, Vargas MI (2014). The fornix and limbic system. Semin Ultrasound CT MR.

[51] Paul LK, Lautzenhiser A, Brown WS, Hart A, Neumann D, Spezio M (2006). Emotional arousal in agenesis of the corpus callosum. Int J Psychophysiol.

[52] Gazzaniga MS (2000). Cerebral specialization and interhemispheric communication: does the corpus callosum enable the human condition?. Brain.

[53] Aoki Y, Abe O, Nippashi Y, Yamasue H (2013). Comparison of white matter integrity between autism spectrum disorder subjects and typically developing individuals: a meta-analysis of diffusion tensor imaging tractography studies. Mol Autism.

[54] Bora E, Fornito A, Radua J, Walterfang M, Seal M, Wood SJ (2011). Neuroanatomical abnormalities in schizophrenia: a multimodal voxelwise meta-analysis and meta-regression analysis. Schizophr Res.

[55] van Erp TGM, Walton E, Hibar DP, Schmaal L, Jiang W, Glahn DC (2018). Cortical brain abnormalities in 4474 individuals with schizophrenia and 5098 control subjects via the Enhancing Neuro Imaging Genetics through Meta Analysis (ENIGMA) consortium. Biol Psychiatry.

[56] Haijma SV, Van Haren N, Cahn W, Koolschijn PC, Hulshoff Pol HE, Kahn RS (2013). Brain volumes in schizophrenia: a meta-analysis in over 18000 subjects. Schizophr Bull.

[57] Arts B, Jabben N, Krabbendam L, van Os J (2008). Meta-analyses of cognitive functioning in euthymic bipolar patients and their first-degree relatives. Psychol Med.

[58] Bora E, Yucel M, Pantelis C (2010). Neurocognitive markers of psychosis in bipolar disorder: a meta-analytic study. J Affect Disord.

[59] Lee J, Altshuler L, Glahn DC, Miklowitz DJ, Ochsner K, Green MF (2013). Social and nonsocial cognition in bipolar disorder and schizophrenia: relative levels of impairment. Am J Psychiatry.

[60] Fujino H, Sumiyoshi C, Yasuda Y, Yamamori H, Fujimoto M, Fukunaga M (2017). Estimated cognitive decline in patients with schizophrenia: a multicenter study. Psychiatry Clin Neurosci.

[61] American psychiatric association. (2013). Diagnostic and statistical manual of mental disorders: DSM-5..

[62] Picchioni MM, Rijsdijk F, Toulopoulou T, Chaddock C, Cole JH, Ettinger U (2017). Familial and environmental influences on brain volumes in twins with schizophrenia. J Psychiatry Neurosci.

[63] Clarke MC, Harley M, Cannon M (2006). The role of obstetric events in schizophrenia. Schizophr Bull.

[64] Cannon M, Jones PB, Murray RM (2002). Obstetric complications and schizophrenia: historical and meta-analytic review. Am J Psychiatry.

