# Supplementary material for: White matter microstructural alterations across four major psychiatric disorders: mega-analysis study in 2937 individuals
Source: Mol Psychiatry. 2019 Nov 29;25(4):883–95. doi: 10.1038/s41380-019-0553-7 (PMC7156346; doi:10.1038/s41380-019-0553-7)
Supplement: Supplementary file 1 — Supplementary text [file 41380_2019_553_MOESM1_ESM.docx]

**Supplementary Information**

**White matter microstructural alterations across four major psychiatric disorders:**

**mega-analysis study in 2937 individuals**

Daisuke Koshiyama, M.D., Ph.D.^1^; Masaki Fukunaga, Ph.D.^2^; Naohiro Okada, M.D., Ph.D.^1,3^; Kentaro Morita, M.D., Ph.D.^1^; Kiyotaka Nemoto, M.D., Ph.D.^4^; Kaori Usui, M.A.^1^; Hidenaga Yamamori, M.D., Ph.D.^5^; Yuka Yasuda, M.D., Ph.D.^6, 7^; Michiko Fujimoto, M.D., Ph.D.^5^; Noriko Kudo, Ph.D.^7^; Hirotsugu Azechi, Ph.D.^8^; Yoshiyuki Watanabe, M.D., Ph.D.^9^; Naoki Hashimoto, M.D., Ph.D.^10^; Hisashi Narita, M.D., Ph.D.^10^; Ichiro Kusumi, M.D., Ph.D.^10^; Kazutaka Ohi, M.D., Ph.D.^11, 12^; Takamitsu Shimada, M.D., Ph.D.^11^; Yuzuru Kataoka, M.D.^11^; Maeri Yamamoto, M.D., Ph.D.^13^; Norio Ozaki, M.D., Ph.D.^13^; Go Okada, M.D., Ph.D.^14^; Yasumasa Okamoto, M.D., Ph.D.^14^; Kenichiro Harada, M.D., Ph.D.^15^; Koji Matsuo, M.D., Ph.D.^16^; Hidenori Yamasue, M.D., Ph.D.^17^; Osamu Abe, M.D., Ph.D.^18^; Ryuichiro Hashimoto, Ph.D.^19^; Tsutomu Takahashi, M.D., Ph.D.^20^; Tomoki Hori, M.D.^21^; Masahito Nakataki, M.D., Ph.D.^22^; Toshiaki Onitsuka, M.D., Ph.D.^23^; Laurena Holleran, Ph.D.^24^; Neda Jahanshad, Ph.D.^25^; Theo G.M. van Erp, Ph.D.^26^; Jessica Turner, Ph.D.^27^; Gary Donohoe, Ph.D.^24^; Paul M. Thompson, Ph.D.^25^; Kiyoto Kasai, M.D., Ph.D.^1,3^; Ryota Hashimoto, M.D., Ph.D.^5, 7, 8^; COCORO

1. Department of Neuropsychiatry, Graduate School of Medicine, The University of Tokyo, Tokyo, Japan
2. Division of Cerebral Integration, National Institute for Physiological Sciences, Aichi, Japan
3. International Research Center for Neurointelligence (WPI-IRCN), UTIAS, The University of Tokyo, Tokyo, Japan
4. Department of Psychiatry, Division of Clinical Medicine, Faculty of Medicine, University of Tsukuba, Tsukuba, Japan
5. Department of Psychiatry, Osaka University Graduate School of Medicine
6. Life Grow Brilliant Mental Clinic, Medical Corporation Foster, Osaka, Japan
7. Department of Pathology of Mental Diseases, National Institute of Mental Health, National Center of Neurology and Psychiatry, Tokyo, Japan
8. Molecular Research Center for Children's Mental Development, United Graduate School of Child Development, Osaka University, Osaka, Japan
9. Diagnostic and Interventional Radiology, Osaka University Graduate School of Medicine, Osaka, Japan
10. Department of Psychiatry, Hokkaido University Graduate School of Medicine, Hokkaido, Japan
11. Department of Neuropsychiatry, Kanazawa Medical University, Ishikawa, Japan
12. Medical Research Institute, Kanazawa Medical University, Ishikawa, Japan
13. Department of Psychiatry, Nagoya University, Graduate School of Medicine, Aichi, Japan
14. Department of Psychiatry and Neurosciences, Graduate School of Biomedical and Health Sciences, Hiroshima University, Hiroshima, Japan
15. Division of Neuropsychiatry, Department of Neuroscience, Yamaguchi University Graduate School of Medicine, Yamaguchi, Japan
16. Department of Psychiatry, Faculty of Medicine, Saitama Medical University, Saitama, Japan
17. Department of Psychiatry, Hamamatsu University School of Medicine, Shizuoka, Japan
18. Department of Radiology, Graduate School of Medicine, The University of Tokyo, Tokyo, Japan
19. Medical Institute of Developmental Disabilities Research, Showa University, Tokyo, Japan
20. Department of Neuropsychiatry, University of Toyama Graduate School of Medicine and Pharmaceutical Sciences, Toyama, Japan
21. Department of Psychiatry, Graduate School of Medicine, Kyoto University, Kyoto, Japan
22. Department of Psychiatry, Tokushima University Hospital, Tokushima, Japan
23. Department of Neuropsychiatry, Graduate School of Medical Sciences, Kyushu University, Fukuoka, Japan
24. Center for Neuroimaging and Cognitive Genomics (NICOG), School of Psychology, National University of Ireland Galway, Galway, Ireland
25. Imaging Genetics Center, Mark and Mary Stevens Neuroimaging and Informatics Institute, Keck School of Medicine, University of Southern California, Marina del Rey, CA, USA
26. Clinical Translational Neuroscience Laboratory, Department of Psychiatry and Human Behavior, University of California Irvine, Irvine, CA, USA
27. Psychology and Neuroscience, Georgia State University, Atlanta, GA, USA

**TABLE OF CONTENTS**

**SUPPLEMENTARY METHODS**

**Supplementary Method 1** Subject inclusion and exclusion criteria by site……….………………….......12

**References of Supplementary Method 1**……….…………...….……………………..………………… 20

**Supplementary Method 2** Detailed imaging parameters for each protocol…….……………..…………..23

**Supplementary Method 3** Variability ratio of the DTI indices…….……..………..……………………..29

**SUPPLEMENTARY TABLES**

**Supplementary Table 1** Basic characteristics of the included protocols in comparison among the patient groups.……….……………….……………………..………………..………………..……………………30

**Supplementary Table 2** Mega-analysis results of variability ratios (VR) of fractional anisotropy (FA) between patients with schizophrenia and healthy comparison subjects.………………..…………….……31

**Supplementary Table 3** Mega-analysis results of variability ratios (VR) of mean diffusivity (MD) between patients with schizophrenia and healthy comparison subjects.………………..……………….…32

**Supplementary Table 4** Mega-analysis results of variability ratios (VR) in axial diffusivity (AD) between patients with schizophrenia and healthy comparison subjects.………………..……………………………33

**Supplementary Table 5** Mega-analysis results of variability ratios (VR) in radial diffusivity (RD) between patients with schizophrenia and healthy comparison subjects.………………..……….…………34

**Supplementary Table 6** Mega-analysis results of variability ratios (VR) in fractional anisotropy (FA) between patients with bipolar disorder and healthy comparison subjects.………………..………………..35

**Supplementary Table 7** Mega-analysis results of variability ratios (VR) in mean diffusivity (MD) between patients with bipolar disorder and healthy comparison subjects.…………………………………36

**Supplementary Table 8** Mega-analysis results of variability ratios (VR) in axial diffusivity (AD) between patients with bipolar disorder and healthy comparison subjects.………………………..…………………37

**Supplementary Table 9** Mega-analysis results of variability ratios (VR) in radial diffusivity (RD) between patients with bipolar disorder and healthy comparison subjects.…………………………………38

**Supplementary Table 10** Mega-analysis results of variability ratios (VR) in fractional anisotropy (FA) between individuals with autism spectrum disorder and healthy comparison subjects.……………………39

**Supplementary Table 11** Mega-analysis results of variability ratios (VR) in mean diffusivity (MD) between individuals with autism spectrum disorder and healthy comparison subjects.……………………40

**Supplementary Table 12** Mega-analysis results of variability ratios (VR) in axial diffusivity (AD) between individuals with autism spectrum disorder and healthy comparison subjects.……………………41

**Supplementary Table 13** Mega-analysis results of variability ratios (VR) in radial diffusivity (RD) between individuals with autism spectrum disorder and healthy comparison subjects.……………………42

**Supplementary Table 14** Mega-analysis results of variability ratios (VR) in fractional anisotropy (FA) between patients with major depressive disorder and healthy comparison subjects.………………………43

**Supplementary Table 15** Mega-analysis results of variability ratios (VR) in mean diffusivity (MD) between patients with major depressive disorder and healthy comparison subjects.………………………44

**Supplementary Table 16** Mega-analysis results of variability ratios (VR) in axial diffusivity (AD) between patients with major depressive disorder and healthy comparison subjects.………………………45

**Supplementary Table 17** Mega-analysis results of variability ratios (VR) in radial diffusivity (RD) between patients with major depressive disorder and healthy comparison subjects.………………………46

**Legends of Supplementary Table 2–17** …….……..……..…….……………………..………………….47

**Supplementary Table 18** Mega-analysis results of differences in fractional anisotropy (FA) between patients with schizophrenia and healthy comparison subjects…….……………………..…………………48

**Supplementary Table 19** Mega-analysis results of differences in mean diffusivity (MD) between patients with schizophrenia and healthy comparison subjects……….…….……………………..…………………49

**Supplementary Table 20** Mega-analysis results of differences in axial diffusivity (AD) between patients with schizophrenia and healthy comparison subjects. ……….…….……………………..………………..50

**Supplementary Table 21** Mega-analysis results of differences in radial diffusivity (RD) between patients with schizophrenia and healthy comparison subjects……….…….……………………..…………………51

**Supplementary Table 22** Mega-analysis results of differences in fractional anisotropy (FA) between patients with bipolar disorder and healthy comparison subjects……….…….……………..………………52

**Supplementary Table 23** Mega-analysis results of differences in mean diffusivity (MD) between patients with bipolar disorder and healthy comparison subjects….……….…….……………………..……………53

**Supplementary Table 24** Mega-analysis results of differences in axial diffusivity (AD) between patients with bipolar disorder and healthy comparison subjects……….…….……………………..……….………54

**Supplementary Table 25** Mega-analysis results of differences in radial diffusivity (RD) between patients with bipolar disorder and healthy comparison subjects……….…….……………………..…….…………55

**Supplementary Table 26** Mega-analysis results of differences in fractional anisotropy (FA) between individuals with autism spectrum disorder and healthy comparison subjects……….…….…...………..…56

**Supplementary Table 27** Mega-analysis results of differences in mean diffusivity (MD) between individuals with autism spectrum disorder and healthy comparison subjects..……….……………..…..…57

**Supplementary Table 28** Mega-analysis results of differences in axial diffusivity (AD) between individuals with autism spectrum disorder and healthy comparison subjects……….………………..……58

**Supplementary Table 29** Mega-analysis results of differences in radial diffusivity (RD) between individuals with autism spectrum disorder and healthy comparison subjects……….…….……...…..……59

**Supplementary Table 30** Mega-analysis results of differences in fractional anisotropy (FA) between patients with major depressive disorder and healthy comparison subjects……….……..…….……………60

**Supplementary Table 31** Mega-analysis results of differences in mean diffusivity (MD) between patients with major depressive disorder and healthy comparison subjects……….…….……………………..…….61

**Supplementary Table 32** Mega-analysis results of differences in axial diffusivity (AD) between patients with major depressive disorder and healthy comparison subjects..……….…….……………...………..…62

**Supplementary Table 33** Mega-analysis results of differences in radial diffusivity (RD) between patients with major depressive disorder and healthy comparison subjects..……….…….……………….…………63

**Legends of Supplementary Table 18–33**…….……..……..…….……………………..…………………64

**Supplementary Table 34** Mega-analysis results of differences in fractional anisotropy (FA) between patients with schizophrenia and patients with bipolar disorder.……………..……………..………………65

**Supplementary Table 35** Mega-analysis results of difference of mean diffusivity (MD) between patients with schizophrenia and patients with bipolar disorder.…………..…………………..………………….…66

**Supplementary Table 36** Mega-analysis results of differences in axial diffusivity (AD) between patients with schizophrenia and patients with bipolar disorder.………………..………………..….………………67

**Supplementary Table 37** Mega-analysis results of differences in radial diffusivity (RD) between patients with schizophrenia and patients with bipolar disorder.……………..………………..……………..……...68

**Supplementary Table 38** Mega-analysis results of differences in fractional anisotropy (FA) between patients with schizophrenia and individuals with autism spectrum disorder.………………………………69

**Supplementary Table 39** Mega-analysis results of differences in mean diffusivity (MD) between patients with schizophrenia and individuals with autism spectrum disorder.………………………………………70

**Supplementary Table 40** Mega-analysis results of differences in axial diffusivity (AD) between patients with schizophrenia and individuals with autism spectrum disorder.………………………………………71

**Supplementary Table 41** Mega-analysis results of differences in radial diffusivity (RD) between patients with schizophrenia and individuals with autism spectrum disorder.………………………………………72

**Supplementary Table 42** Mega-analysis results of differences in fractional anisotropy (FA) between patients with schizophrenia and patients with major depressive disorder.…………………………………73

**Supplementary Table 43** Mega-analysis results of differences in mean diffusivity (MD) between patients with schizophrenia and patients with major depressive disorder.…………………………………………74

**Supplementary Table 44** Mega-analysis results of differences in axial diffusivity (AD) between patients with schizophrenia and patients with major depressive disorder.…………………………………………75

**Supplementary Table 45** Mega-analysis results of differences in radial diffusivity (RD) between patients with schizophrenia and patients with major depressive disorder.…………………………………………76

**Supplementary Table 46** Mega-analysis results of differences in fractional anisotropy (FA) between patients with bipolar disorder and individuals with autism spectrum disorder.……………………………77

**Supplementary Table 47** Mega-analysis results of differences in mean diffusivity (MD) between patients with bipolar disorder and individuals with autism spectrum disorder.……………………………………78

**Supplementary Table 48** Mega-analysis results of differences in axial diffusivity (AD) between patients with bipolar disorder and individuals with autism spectrum disorder.……………………………………79

**Supplementary Table 49** Mega-analysis results of differences in radial diffusivity (RD) between patients with bipolar disorder and individuals with autism spectrum disorder.……………………………………80

**Supplementary Table 50** Mega-analysis results of differences in fractional anisotropy (FA) between patients with bipolar disorder and patients with major depressive disorder.………………………………81

**Supplementary Table 51** Mega-analysis results of differences in mean diffusivity (MD) between patients with bipolar disorder and patients with major depressive disorder.…………..……………………………82

**Supplementary Table 52** Mega-analysis results of differences in axial diffusivity (AD) between patients with bipolar disorder and patients with major depressive disorder.………………………………………83

**Supplementary Table 53** Mega-analysis results of differences in radial diffusivity (RD) between patients with bipolar disorder and patients with major depressive disorder.………………………………………84

**Supplementary Table 54** Mega-analysis results of differences in fractional anisotropy (FA) between individuals with autism spectrum disorder and patients with major depressive disorder.…………………85

**Supplementary Table 55** Mega-analysis results of differences in mean diffusivity (MD) between individuals with autism spectrum disorder and patients with major depressive disorder.…………………86

**Supplementary Table 56** Mega-analysis results of differences in axial diffusivity (AD) between individuals with autism spectrum disorder and patients with major depressive disorder.…………………87

**Supplementary Table 57** Mega-analysis results of differences in radial diffusivity (RD) between individuals with autism spectrum disorder and patients with major depressive disorder.…………………88

**Legends of Supplementary Table 34–57**……………..……….………………..………………………...89

**Supplementary Table 58** Mega-analysis results of correlation between fractional anisotropy (FA) and duration of illness in patients with schizophrenia…………...…….……………………..…………………90

**Supplementary Table 59** Mega-analysis results of correlation between mean diffusivity (MD) and duration of illness in patients with schizophrenia………..….…….……………………..…………………91

**Supplementary Table 60** Mega-analysis results of correlation between axial diffusivity (AD) and duration of illness in patients with schizophrenia…………………….…….……………………..…………………92

**Supplementary Table 61** Mega-analysis results of correlation between radial diffusivity (RD) and duration of illness in patients with schizophrenia…………………….…….………………………………93

**Supplementary Table 62** Mega-analysis results of correlation between fractional anisotropy (FA) and duration of illness in patients with bipolar disorder…………………….…….……………………………94

**Supplementary Table 63** Mega-analysis results of correlation between mean diffusivity (MD) and duration of illness in patients with bipolar disorder…………………….…….……………………………95

**Supplementary Table 64** Mega-analysis results of correlation between axial diffusivity (AD) and duration of illness in patients with bipolar disorder…………………….…….……………………..………………96

**Supplementary Table 65** Mega-analysis results of correlation between radial diffusivity (RD) and duration of illness in patients with bipolar disorder…………………….…….……………………………97

**Supplementary Table 66** Mega-analysis results of correlation between fractional anisotropy (FA) and duration of illness (age) in individuals with autism spectrum disorder……………..….…….…….………98

**Supplementary Table 67** Mega-analysis results of correlation between mean diffusivity (MD) and duration of illness (age) in individuals with autism spectrum disorder……………………….……………99

**Supplementary Table 68** Mega-analysis results of correlation between axial diffusivity (AD) and duration of illness (age) in individuals with autism spectrum disorder…………………….…….………..……..…100

**Supplementary Table 69** Mega-analysis results of correlation between radial diffusivity (RD) and duration of illness (age) in individuals with autism spectrum disorder…………………….…………..…101

**Supplementary Table 70** Mega-analysis results of correlation between fractional anisotropy (FA) and duration of illness in patients with major depressive disorder…………………….…….……………...…102

**Supplementary Table 71** Mega-analysis results of correlation between mean diffusivity (MD) and duration of illness in patients with major depressive disorder…………………….…….……………...…103

**Supplementary Table 72** Mega-analysis results of correlation between axial diffusivity (AD) and duration of illness in patients with major depressive disorder…………………….…….………………...…..……104

**Supplementary Table 73** Mega-analysis results of correlation between radial diffusivity (RD) and duration of illness in patients with major depressive disorder…………………….………………..…..…105

**Legends of Supplementary Table 58–73**……………..……….………………..……………………….106

**Supplementary Table 74** Mega-analysis results of correlation between fractional anisotropy (FA) and chlorpromazine equivalent dose in patients with schizophrenia…………………….………………….....107

**Supplementary Table 75** Mega-analysis results of correlation between mean diffusivity (MD) and chlorpromazine equivalent dose in patients with schizophrenia…………………….……………..…...…108

**Supplementary Table 76** Mega-analysis results of correlation between axial diffusivity (AD) and chlorpromazine equivalent dose in patients with schizophrenia…………………….………………….....109

**Supplementary Table 77** Mega-analysis results of correlation between radial diffusivity (RD) and chlorpromazine equivalent dose in patients with schizophrenia…………………….………………….…110

**Supplementary Table 78** Mega-analysis results of correlation between fractional anisotropy (FA) and lithium dose in patients with bipolar disorder……………………………………….…………………….111

**Supplementary Table 79** Mega-analysis results of correlation between mean diffusivity (MD) and lithium dose in patients with bipolar disorder…………………….…….…………………………..……………..112

**Supplementary Table 80** Mega-analysis results of correlation between axial diffusivity (AD) and lithium dose in patients with bipolar disorder…………………….…….…………………………..……………..113

**Supplementary Table 81** Mega-analysis results of correlation between radial diffusivity (RD) and lithium dose in patients with bipolar disorder………………………….…….……………………..……………..114

**Supplementary Table 82** Mega-analysis results of correlation between fractional anisotropy (FA) and imipramine equivalent dose in patients with major depressive disorder…………………….…….……...115

**Supplementary Table 83** Mega-analysis results of correlation between mean diffusivity (MD) and imipramine equivalent dose in patients with major depressive disorder…………………...…………..…116

**Supplementary Table 84** Mega-analysis results of correlation between axial diffusivity (AD) and imipramine equivalent dose in patients with major depressive disorder…………………….……..……..117

**Supplementary Table 85** Mega-analysis results of correlation between radial diffusivity (RD) and imipramine equivalent dose in patients with major depressive disorder…………….……….…….…..…118

**Abbreviations of Supplementary Table 74–85**…………………………………………..…………..…119

**SUPPLEMENTARY FIGURES**

**Supplementary Figure 1** Differences in mean diffusivity (MD) between patients with schizophrenia (SZ) and healthy comparison subjects (HCS) …………………….…….……………………..……………….120

**Supplementary Figure 2** Differences in axial diffusivity (AD) between patients with schizophrenia (SZ) and healthy comparison subjects (HCS) …………………….…….……………………..……………….120

**Supplementary Figure 3** Differences in radial diffusivity (RD) between patients with schizophrenia (SZ) and healthy comparison subjects (HCS) …………………….…….……………………..……………….121

**Abbreviations of Supplementary Figure 1–3** ...…………………….……...……..…………….………121

**Supplementary Figure 4** Effect size of the DTI indices between patients with psychiatric disorders and healthy comparison subjects in each white matter regions other than main findings.…….………………122

**Supplementary Figure 5** Forest plot of effect sizes for each cohort for differences of DTI indices in the patients with psychiatric disorders versus healthy comparison subjects..…….……………….………......128

**Supplementary Figure 6** Differences in fractional anisotropy (FA) between patients with bipolar disorder (BPD) and healthy comparison subjects (HCS).…………………….…….…………………....…………232

**Supplementary Figure 7** Differences in mean diffusivity (MD) between patients with bipolar disorder (BPD) and healthy comparison subjects (HCS) .…………………….…….…………………...…………232

**Supplementary Figure 8** Differences in axial diffusivity (AD) between patients with bipolar disorder (BPD) and healthy comparison subjects (HCS) .…………………….…….…………………...…………233

**Supplementary Figure 9** Differences in radial diffusivity (RD) between patients with bipolar disorder (BPD) and healthy comparison subjects (HCS) .…………………….…….…………………...…………233

**Supplementary Figure 10** Differences in fractional anisotropy (FA) between individuals with autism spectrum disorder (ASD) and healthy comparison subjects (HCS)..…………………….…….………….234

**Supplementary Figure 11** Differences in mean diffusivity (MD) between individuals with autism spectrum disorder (ASD) and healthy comparison subjects (HCS)...………….…….……………………234

**Supplementary Figure 12** Differences in axial diffusivity (AD) between individuals with autism spectrum disorder (ASD) and healthy comparison subjects (HCS)...…………………….…….……………………235

**Supplementary Figure 13** Differences in radial diffusivity (RD) between individuals with autism spectrum disorder (ASD) and healthy comparison subjects (HCS)...…………………….…….…………235

**Supplementary Figure 14** Differences in fractional anisotropy (FA) between patients with major depressive disorder (MDD) and healthy comparison subjects (HCS)...…………………….…….………236

**Supplementary Figure 15** Differences in mean diffusivity (MD) between patients with major depressive disorder (MDD) and healthy comparison subjects (HCS)..…………………….…….…………………...236

**Supplementary Figure 16** Differences in axial diffusivity (AD) between patients with major depressive disorder (MDD) and healthy comparison subjects (HCS)…………………….…….………………….…237

**Supplementary Figure 17** Differences in radial diffusivity (RD) between patients with major depressive disorder (MDD) and healthy comparison subjects (HCS)...………………….…….………………….….237

**Abbreviations of Supplementary Figure 6–17** ...…………………….……...………………….………..238

**SUPPLEMENTARY METHODS**

**Supplementary Method 1** Subject inclusion and exclusion criteria by site

Participants recruited from the Osaka site had no biological relations, and all of them were of Japanese descent.^1-6^ The subjects were excluded if they had neurological or medical conditions that could potentially affect the central nervous system, such as atypical headaches, head trauma with loss of consciousness, chronic lung disease, kidney disease, chronic hepatic disease, thyroid disease, active cancer, cerebrovascular disease, epilepsy, seizures, substance-related disorders, or mental retardation. Patients with schizophrenia, bipolar disorder, autism spectrum disorder, and major depressive disorder were recruited from the Osaka University Hospital. Each patient had been diagnosed by at least two trained psychiatrists according to the criteria from the diagnostic and statistical manual of mental disorders, fourth edition (DSM-IV) based on the structured clinical interview for DSM-IV (SCID).^7^ Controls were recruited through local advertisements at Osaka University. Healthy comparison subjects were evaluated using the non-patient version of the SCID ^8^ to exclude individuals who had current or past contact with psychiatric services or who had received psychiatric medications.

Participants at the Hokkaido site were excluded if they had physical conditions that could potentially influence the central nervous system. Patients whose diagnosis fell under any of the following were recruited from the Hokkaido University Hospital; schizophrenia, bipolar disorder, and major depressive disorder. All patients were diagnosed by psychiatrists with at least 6 years of clinical experience according to the DSM-IV or DSM-5 criteria. Healthy comparison subjects were recruited through local advertisements at Hokkaido University. All healthy comparison subjects were screened to exclude any psychiatric disorders using the Japanese version of the mini international neuropsychiatric interview (M.I.N.I.) ^9^ by trained psychiatrists.

Inclusion and exclusion criteria for participants at the Tokyo A and B site has been described elsewhere.^10-12^ Briefly, the diagnosis of patients with schizophrenia was determined according to the SCID-I clinical version. Healthy comparison subjects were screened for neuropsychiatric disorders through the SCID-I non-patient edition. The exclusion criteria for both groups were current or past neurological illness, previous traumatic brain injury with any known cognitive consequences or loss of consciousness for more than 5 min, history of electroconvulsive therapy, autism spectrum disorder that met DSM-IV criteria, and previous substance abuse or dependence based on clinical histories. Additional exclusion criteria for the healthy comparison subjects were a history of psychiatric disease in the subjects themselves or of axis I disorders amongst their first-degree relatives.

Participants at the Tokyo C were excluded if they had physical conditions that might potentially influence the central nervous system. Patients with schizophrenia were recruited from the University of Tokyo Hospital, and all patients were diagnosed by trained psychiatrists according to the DSM-IV criteria. Healthy comparison subjects were sampled from the Japanese study of stratification, health, income, and neighborhood (J-SHINE) survey.^13^ All healthy comparison subjects were screened to exclude any psychiatric disorders using the M.I.N.I., which was administered by trained psychiatrists, or using the composite international diagnostic interview (CIDI), which was administered by trained interviewers.

Patients with schizophrenia, bipolar disorder, and major depressive disorder at the Tokyo D site were diagnosed according to the SCID-I clinical version. Healthy comparison subjects were screened for neuropsychiatric disorders through the M.I.N.I. The exclusion criteria for both groups were current or past neurological illness, previous traumatic brain injury with any known cognitive consequences or loss of consciousness for more than 5 min, history of electroconvulsive therapy, and previous substance abuse or dependence based on clinical histories. Additional exclusion criteria for the healthy comparison subjects were a history of psychiatric disease.

All subjects recruited in Kanazawa site were of Japanese descent, and all were biologically unrelated to at least the second degree. Patients were recruited from both the outpatient and inpatient populations at Kanazawa Medical University Hospital.^14-18^ Each patient with schizophrenia, bipolar disorder, or major depressive disorder had been diagnosed by at least two trained psychiatrists on the basis of unstructured clinical interviews, medical records and clinical conferences. Diagnoses were made according to criteria in the DSM-5. Healthy comparison subjects were recruited through local advertisements and from among hospital staff at Kanazawa Medical University. Healthy comparison subjects were evaluated using unstructured psychiatric interviews to exclude individuals who had had current or past contact with psychiatric services or who had received psychiatric medication. Subjects were excluded from analysis if they had neurological or medical conditions that could affect the central nervous system, including head trauma with loss of consciousness, chronic lung disease, chronic hepatic disease, kidney disease, active cancer, cerebrovascular disease, seizures, epilepsy, substance-related disorders or intellectual disorder.^14-18^ Written informed consent was obtained from all subjects after the procedures were fully explained. This study was performed according to the world medical association’s declaration of Helsinki and was approved by the research ethical committee of Kanazawa Medical University.

Participants at the Nagoya site were excluded if they had physical conditions that might potentially influence the central nervous system. All patients (schizophrenia, bipolar disorder and autism spectrum disorder) were recruited from Nagoya University Hospital and affiliated psychiatric hospitals. All patients were diagnosed by trained psychiatrists according to the DSM-IV criteria. Healthy comparison subjects were recruited from the local community, hospital staff, and university students at Nagoya University and affiliated psychiatric hospitals. Healthy comparison subjects were evaluated using the non-patient version of the SCID to exclude individuals who had current or past contact with psychiatric services or who had received psychiatric medications.

Participants recruited from the Hiroshima site were all right-handed, native speakers of Japanese. Patients with bipolar disorder and major depressive disorder were recruited from Hiroshima University Hospital or local clinics in Hiroshima city. The patients were diagnosed by a senior psychiatrist according to the criteria from the DSM-5 beforehand, and the M.I.N.I. was performed at the time of participation in the study to confirm the diagnosis. Healthy comparison subjects were recruited through a newspaper advertisement. These participants were interviewed prior to enrollment by a trained psychiatrist using the M.I.N.I. Participants were excluded from all groups if they had diagnosis of schizophrenia, alcohol and substance abuse/dependence, dementia, developmental disorders, eating disorders, personality disorder, or severe physical illness, or if they had high-level suicide risk, or if they were currently breast-feeding during pregnancy or in the postpartum period. Individuals who demonstrated a history of psychiatric illness or contact with psychiatric services were excluded from the healthy comparison subject group.

The Yamaguchi sample was collected from 2008 to 2015. Patients were recruited from Yamaguchi University Hospital and referred by area clinics and hospitals. Patients met the DSM-IV-text revision (TR) criteria for bipolar disorder and major depressive disorder by M.I.N.I., clinical interviews, and case conferences by senior psychiatrists. Healthy participants were recruited by advertisements and word-of-mouth in the surrounding community. Patients with current or history of substance abuse or dependence and other psychotic illnesses were excluded. Healthy comparison subjects were screened during clinical interviews using the M.I.N.I. Healthy comparison subjects with immediate family members having any psychiatric disorder were excluded. Based on interviews, blood tests and physical examinations, subjects with an endocrinological disease, head trauma, neurological disease, family history of any hereditary neurological disorder, or other medical conditions (e.g., hypertension, diabetes, active liver disease, kidney problems, or respiratory problems) were also excluded. A depressed state was defined by a score greater than 18 on the Hamilton depression rating scale (HDRS).^19^ Subjects also participated in interviews to obtain clinical demographics. This study protocol was approved by the institutional review board of Yamaguchi University Hospital.

Participants recruited from the Showa site had no biological relations, and all of them were of Japanese descent. The subjects were excluded if they had neurological or medical conditions that could potentially affect the central nervous system. Individuals with autism spectrum disorder were recruited from the Showa University Karasuyama Hospital. The diagnostic procedure for individuals with autism spectrum disorder was the same as our previous studies.^20-23^ Briefly, experienced psychiatrists carefully diagnosed the patients as autism spectrum disorder if there was a consensus between the psychiatrist and clinical psychologist who interviewed the patients independently based on the DSM-IV-TR. Healthy comparison subjects were recruited by advertisements and acquaintances. None of the healthy comparison subjects reported any severe medical problem or any neurological or psychiatric history. Moreover, the M.I.N.I. was used to confirm that none of the healthy comparison subjects met the diagnostic criteria for any psychiatric disorder.

All subjects recruited from the Toyama site were Japanese and physically healthy at the time of the study. None had a lifetime history of serious head trauma, neurological illness, serious medical or surgical illness, or substance abuse disorders. Patients with schizophrenia fulfilling the DSM-IV-TR criteria were recruited from the in- and outpatient clinics of the department of neuropsychiatry of Toyama University Hospital. They were diagnosed based on information obtained from a clinical assessment using the SCID-I, a detailed chart review, as well as the clinical symptoms rated at the time of scanning. Healthy comparison subjects, who were screened for psychiatric illness using the SCID-I non-patient edition, were recruited from members of the local community, hospital staff, and university students. They were asked to complete a questionnaire consisting of 19 items concerning their personal (17 items; including a history of obstetric complications, substantial head injury, seizures, neurological illness, impaired thyroid function, hypertension, diabetes, and substance abuse) and family (2 items) histories of illness. Subjects with family history of psychiatric illness among their first-degree relatives were excluded.

Patients with schizophrenia recruited at Kyoto University included diagnoses of schizophrenia, schizoaffective disorder, and schizophreniform disorder. They were not comorbid with any other DSM-IV axis I psychiatric disorders. Healthy comparison subjects were recruited by local advertisements and word of mouth. They had no history of psychiatric illness. Exclusion criteria for all participants included a history of head trauma, neurological illness, mental retardation, and serious medical or surgical illness.

Participants recruited from Tokushima University Hospital had no biological relations, and all of them were of Japanese descent. The patients had been diagnosed with schizophrenia based on the criteria in the DSM-IV. At the time enrolment, all patients were clinically stable, as judged by a therapeutic psychiatrist. The criteria for determining clinical stability were no schedule to change treatment contents; essentially no psychopathological changes; judgment of clinically stable by a therapeutic psychiatrist and patients themselves. Each patient’s clinical stability was assessed from medical records, self-reports, and the observations of psychiatric staff and relatives. The exclusion criteria were a past history or presence of any serious disorders affecting the brain or cognitive functioning, such as epilepsy, serious head injury, or brain tumor; alcohol abuse; active drug use in the past year; or pregnancy or intention to become pregnant during the study period. Healthy comparison subjects were evaluated with structured clinical interviews to confirm the absence of schizophrenia and had no history of neurological or psychiatric disorders or any first-degree relatives with psychotic episodes.

All subjects recruited from the Kyushu site were Japanese and physically healthy at the time of the study. The exclusion criteria were: 1) neurological illness or major head trauma; 2) electroconvulsive therapy; 3) alcohol or drug dependence; 4) alcohol or drug abuse within the past 5 years. Healthy comparison subjects were screened using the SCID non-patient edition. No healthy comparison subjects had an Axis-I psychiatric disorder themselves or amongst their first-degree relatives. All patients were recruited from Hoaki Hospital and were diagnosed by at least two trained psychiatrists based on the SCID-DSM IV and medical records.**References of Supplementary Method 1**

1. Hashimoto R, Ohi K, Yasuda Y, Fukumoto M, Yamamori H, Takahashi H *et al.* Variants of the RELA gene are associated with schizophrenia and their startle responses. *Neuropsychopharmacology* 2011; **36:** 1921-1931.

2. Ohi K, Hashimoto R, Yasuda Y, Nemoto K, Ohnishi T, Fukumoto M *et al.* Impact of the genome wide supported NRGN gene on anterior cingulate morphology in schizophrenia. *PLoS One* 2012; **7:** e29780.

3. Hashimoto R, Ohi K, Yasuda Y, Fukumoto M, Yamamori H, Kamino K *et al.* The KCNH2 gene is associated with neurocognition and the risk of schizophrenia. *World J Biol Psychiatry* 2013; **14:** 114-120.

4. Ohi K, Hashimoto R, Yasuda Y, Kiribayashi M, Iike N, Yoshida T *et al.* TATA box-binding protein gene is associated with risk for schizophrenia, age at onset and prefrontal function. *Genes Brain Behav* 2009; **8:** 473-480.

5. Hashimoto R, Ohi K, Yasuda Y, Fukumoto M, Iwase M, Iike N *et al.* The impact of a genome-wide supported psychosis variant in the ZNF804A gene on memory function in schizophrenia. *Am J Med Genet B Neuropsychiatr Genet* 2010; **153b:** 1459-1464.

6. Hashimoto R, Ikeda M, Yamashita F, Ohi K, Yamamori H, Yasuda Y *et al.* Common variants at 1p36 are associated with superior frontal gyrus volume. *Transl Psychiatry* 2014; **4:** e472.

7. First M, Spitzer R, Gibbon M, Williams J. *Structured Clinical Interview for DSM-IV axis I disorders. Clinical version.* American Psychiatric Press: Washington, 1997. (Japanese translation: Kitamura T, Okano T. Nihon Hyoron-sha publishers; Tokyo, 2003)

8. First M, Spitzer R, Gibbon M, Williams J. *Structured Clinical Interview for DSM-IV axis I disorders, non-patient edition.* Biometrics Research Department, New York State Psychiatric Institute: New York, 1997. (Japanese translation: Kitamura T, Okano T. Nihon Hyoron-sha publishers: Tokyo, 2003)

9. Otsubo T, Tanaka K, Koda R, Shinoda J, Sano N, Tanaka S *et al.* Reliability and validity of Japanese version of the Mini-International Neuropsychiatric Interview. *Psychiatry Clin Neurosci* 2005; **59:** 517-526.

10. Iwashiro N, Suga M, Takano Y, Inoue H, Natsubori T, Satomura Y *et al.* Localized gray matter volume reductions in the pars triangularis of the inferior frontal gyrus in individuals at clinical high-risk for psychosis and first episode for schizophrenia. *Schizophr Res* 2012; **137:** 124-131.

11. Natsubori T, Inoue H, Abe O, Takano Y, Iwashiro N, Aoki Y *et al.* Reduced frontal glutamate + glutamine and N-acetylaspartate levels in patients with chronic schizophrenia but not in those at clinical high risk for psychosis or with first-episode schizophrenia. *Schizophr Bull* 2014; **40:** 1128-1139.

12. Natsubori T, Hashimoto R, Yahata N, Inoue H, Takano Y, Iwashiro N *et al.* An fMRI study of visual lexical decision in patients with schizophrenia and clinical high-risk individuals. *Schizophr Res* 2014; **157:** 218-224.

13. Takada M, Kondo N, Hashimoto H. Japanese study on stratification, health, income, and neighborhood: study protocol and profiles of participants. *J Epidemiol* 2014; **24:** 334-344.

14. Ohi K, Shimada T, Kihara H, Yasuyama T, Sawai K, Matsuda Y *et al.* Impact of familial loading on prefrontal activation in major psychiatric disorders: a Near-Infrared Spectroscopy (NIRS) Study. *Sci Rep* 2017; **7:** 44268.

15. Ohi K, Shimada T, Nemoto K, Kataoka Y, Yasuyama T, Kimura K *et al.* Cognitive clustering in schizophrenia patients, their first-degree relatives and healthy subjects is associated with anterior cingulate cortex volume. *Neuroimage Clin* 2017; **16:** 248-256.

16. Yasuyama T, Ohi K, Shimada T, Uehara T, Kawasaki Y. Differences in social functioning among patients with major psychiatric disorders: Interpersonal communication is impaired in patients with schizophrenia and correlates with an increase in schizotypal traits. *Psychiatry Res* 2017; **249:** 30-34.

17. Ohi K, Kataoka Y, Shimada T, Kuwata A, Okubo H, Kimura K *et al.* Meta-analysis of physical activity and effects of social function and quality of life on the physical activity in patients with schizophrenia. *Eur Arch Psychiatry Clin Neurosci* 2018.

18. Ohi K, Matsuda Y, Shimada T, Yasuyama T, Oshima K, Sawai K *et al.* Structural alterations of the superior temporal gyrus in schizophrenia: detailed subregional differences. *Eur Psychiatry* 2016; **35:** 25-31.

19. Hamilton M. A rating scale for depression. *J Neurol Neurosurg Psychiatry* 1960; **23:** 56-62.

20. Itahashi T, Yamada T, Watanabe H, Nakamura M, Jimbo D, Shioda S *et al.* Altered network topologies and hub organization in adults with autism: a resting-state fMRI study. *PLoS One* 2014; **9:** e94115.

21. Lin IF, Kashino M, Ohta H, Yamada T, Tani M, Watanabe H *et al.* The effect of intranasal oxytocin versus placebo treatment on the autonomic responses to human sounds in autism: a single-blind, randomized, placebo-controlled, crossover design study. *Mol Autism* 2014; **5:** 20.

22. Ohta H, Yamada T, Watanabe H, Kanai C, Tanaka E, Ohno T *et al.* An fMRI study of reduced perceptual load-dependent modulation of task-irrelevant activity in adults with autism spectrum conditions. *Neuroimage* 2012; **61:** 1176-1187.

23. Watanabe H, Nakamura M, Ohno T, Itahashi T, Tanaka E, Ohta H *et al.* Altered orbitofrontal sulcogyral patterns in adult males with high-functioning autism spectrum disorders. *Soc Cogn Affect Neurosci* 2014; **9:** 520-528.

**Supplementary Method 2** Detailed imaging parameters for each protocol

In the Osaka B group, whole-brain axial DTI scanning was performed on a 3.0 T GE DISCOVERY 750 scanner using an HNS coil with the following parameters: two-dimensional diffusion-weighted spin-echo echo planar imaging (EPI), repetition time (TR) = 12 s, echo time (TE) = 61.1 ms, acquisition matrix = 128 × 128, reconstruction matrix = 256 × 256, ASSET (Array coil Spatial Sensitivity Encoding) acceleration factor = 2, field of view (FOV) = 240 × 240 mm, slice thickness = 2.6 mm, voxel size = 0.94 × 0.94 × 2.6 mm, number of slices = 60. A diffusion sensitization gradient was applied with 15 noncollinear gradient directions and b values of 0 and 1000 s/mm^2^.

In the Hokkaido A group, whole-brain axial DTI scanning was performed on a 1.5 T Siemens Symphony scanner with the following parameters: two-dimensional diffusion-weighted spin-echo EPI, TR = 5.1 s, TE = 139 ms, acquisition matrix = 128 × 97, reconstruction matrix = 256 × 256, FOV = 240 × 240 mm, slice thickness = 6.5 mm, voxel size = 0.94 × 0.94 × 6.5 mm, number of slices = 23. A diffusion sensitization gradient was applied with 12 noncollinear gradient directions and b values of 0 and 1000 s/mm^2^.

In the Osaka A group, whole-brain axial DTI scanning was performed on a 3.0 T GE Signa HDxt scanner using an eight-channel brain coil with the following parameters: two-dimensional diffusion-weighted spin-echo EPI, TR = 15 s, TE = 82.9 ms, acquisition matrix = 96 × 96, reconstruction matrix = 256 × 256, ASSET acceleration factor = 2, FOV = 260 × 260 mm, slice thickness = 3 mm, voxel size = 1.016 × 1.016 × 3 mm, number of slices = 48. A diffusion sensitization gradient was applied with 15 non-collinear gradient directions and b values of 0 and 1000 s/mm^2^.

In the Kanazawa group, whole-brain axial DTI scanning was performed on a 3.0 T Siemens MAGNETOM Trio A Tim System scanner using an 32-channel brain coil with the following parameters: two-dimensional diffusion-weighted spin-echo EPI, TR = 5 s, TE = 81 ms, acquisition matrix = 128 × 128, reconstruction matrix = 128 × 128, GRAPPA (GeneRalized Autocalibrating Partial Parallel Acquisition) acceleration factor = 3, FOV = 240 × 240 mm, slice thickness = 3 mm, voxel size = 1.88 × 1.88 × 3 mm, number of slices = 50. A diffusion sensitization gradient was applied with 64 non-collinear gradient directions and b values of 0 and 1000 s/mm^2^.

In the Nagoya group, whole-brain axial DTI scanning was performed on a 3.0 T Siemens Verio scanner using an 32-channel brain coil with the following parameters: two-dimensional diffusion-weighted spin-echo EPI, TR = 9.4 s, TE = 92 ms, acquisition matrix = 98 × 98, reconstruction matrix = 98 × 98, GRAPPA acceleration factor = 2, FOV = 196 × 196 mm, slice thickness = 2 mm, voxel size = 2 × 2 × 2 mm, number of slices = 60. A diffusion sensitization gradient was applied with 64 non-collinear gradient directions and b values of 0 and 1000 s/mm^2^.

In the Hiroshima group, whole-brain axial DTI scanning was performed on a 3.0 T Siemens MAGNETOM Verio.Dot scanner using an 12-channel brain coil with the following parameters: two-dimensional diffusion-weighted spin-echo EPI, TR = 8.1 s, TE = 94 ms, acquisition matrix = 96 × 96, reconstruction matrix = 96 × 96, GRAPPA acceleration factor = 2, FOV = 240 × 240 mm, slice thickness = 2.5 mm, voxel size = 2.5 × 2.5 × 2.5 mm, number of slices = 60. A diffusion sensitization gradient was applied with 30 non-collinear gradient directions and b values of 0 and 1000 s/mm^2^.

In the Yamaguchi group, whole-brain axial DTI scanning was performed on a 3.0 T Siemens Skyra scanner using an 20-channel brain coil with the following parameters: two-dimensional diffusion-weighted spin-echo EPI, TR = 8.7 s, TE = 88 ms, acquisition matrix = 100 × 100, reconstruction matrix = 100 × 100, GRAPPA acceleration factor = 2, FOV = 235 × 235 mm, slice thickness = 2.4 mm, voxel size = 2.4 × 2.4 × 2.4 mm, number of slices = 70. A diffusion sensitization gradient was applied with 30 non-collinear gradient directions and b values of 0 and 1000 s/mm^2^.

In the Showa group, whole-brain axial DTI scanning was performed on a 3.0 T Siemens Verio scanner using an 12-channel brain coil with the following parameters: two-dimensional diffusion-weighted spin-echo EPI, TR = 13.7 s, TE = 79 ms, acquisition matrix = 100 × 100, reconstruction matrix = 100 × 100, GRAPPA acceleration factor = 2, FOV = 200 × 200 mm, slice thickness = 2 mm, voxel size = 2 × 2 × 2 mm, number of slices = 74. A diffusion sensitization gradient was applied with 65 non-collinear gradient directions and b values of 0 and 1000 s/mm^2^.

In the Kyoto group, whole-brain axial DTI scanning was performed on a 3.0 T Siemens Trio scanner using an eight-channel brain coil with the following parameters: two-dimensional diffusion-weighted spin-echo EPI, TR = 10.5 s, TE = 96 ms, acquisition matrix = 96 × 96, reconstruction matrix = 96 × 96, FOV = 192 × 192 mm, slice thickness = 2 mm, voxel size = 2 × 2 × 2 mm, number of slices = 70. A diffusion sensitization gradient was applied with 81 non-collinear gradient directions and b values of 0 and 1500 s/mm^2^.

In the Tokyo A group, whole-brain axial DTI scanning was performed on a 3.0 T GE Signa scanner using an eight-channel brain coil with the following parameters: two-dimensional diffusion-weighted spin-echo EPI, TR = 20 s, TE = 55.3 ms, acquisition matrix = 128 × 128, reconstruction matrix = 256 × 256, ASSET acceleration factor = 2, FOV = 240 × 240 mm, slice thickness = 2.4 mm, voxel size = 0.938 ×0.938 × 2.4 mm, number of slices = 67. A diffusion sensitization gradient was applied with 30 non-collinear gradient directions and b values of 0 and 1000 s/mm^2^.

In the Tokyo D group, whole-brain axial DTI scanning was performed on a 3.0 T GE Discovery 750w scanner using an 24-channel brain coil with the following parameters: two-dimensional diffusion-weighted spin-echo EPI, TR = 13 s, TE = 86.1 ms, acquisition matrix = 96 × 96, reconstruction matrix = 128 × 128, ASSET acceleration factor = 2, FOV = 240 × 240 mm, slice thickness = 2.5 mm, voxel size = 1.875 ×1.875 × 2.5 mm, number of slices = 60. A diffusion sensitization gradient was applied with 30 non-collinear gradient directions and b values of 0, 1000, 1500 and 2000 s/mm^2^.

In the Toyama A group, whole-brain axial DTI scanning was performed on a 3.0 T Siemens Verio scanner using an 12-channel brain coil with the following parameters: two-dimensional diffusion-weighted spin-echo EPI, TR = 11.5 s, TE = 87 ms, acquisition matrix = 112 × 112, reconstruction matrix = 112 × 112, GRAPPA acceleration factor = 2, FOV = 224 × 224 mm, slice thickness = 2 mm, voxel size = 2 × 2 × 2 mm, number of slices = 75. A diffusion sensitization gradient was applied with 30 non-collinear gradient directions and b values of 0 and 1000 s/mm^2^.

In the Hokkaido B group, whole-brain axial DTI scanning was performed on a 3.0 T Philips Achieva scanner using an 32-channel brain coil with the following parameters: two-dimensional diffusion-weighted spin-echo EPI, TR = 5.052 s, TE = 85 ms, acquisition matrix = 76 × 74, reconstruction matrix = 128 × 128, SENSE (Sensitivity Encoding) acceleration factor = 2, FOV = 224 × 224 mm, slice thickness = 3 mm, voxel size = 1.75 × 1.75 × 3 mm, number of slices = 43. A diffusion sensitization gradient was applied with 32 non-collinear gradient directions and b values of 0, 1000, and 2000 s/mm^2^.

In the Tokyo B group, whole-brain axial DTI scanning was performed on a 3.0 T GE Discovery MR750W scanner using an 32-channel brain coil with the following parameters: two-dimensional diffusion-weighted spin-echo EPI, TR = 16 s, TE = 95.7 ms, acquisition matrix = 128 × 128, reconstruction matrix = 256 × 256, ASSET acceleration factor = 2, FOV = 256 × 256 mm, slice thickness = 2.5 mm, voxel size = 1 × 1 × 2.5 mm, number of slices = 64. A diffusion sensitization gradient was applied with 30 non-collinear gradient directions and b values of 0 and 1000 s/mm^2^.

In the Tokushima group, whole-brain axial DTI scanning was performed on a 3.0 T GE Signa scanner using an 32-channel brain coil with the following parameters: two-dimensional diffusion-weighted spin-echo EPI, TR = 16 s, TE = 82.2 ms, acquisition matrix = 128 × 128, reconstruction matrix = 256 × 256, ASSET acceleration factor = 2, FOV = 240 × 240 mm, slice thickness = 2.9 mm, voxel size = 0.938 ×0.938 × 2.9 mm, number of slices = 64. A diffusion sensitization gradient was applied with 25 non-collinear gradient directions and b values of 0 and 1000 s/mm^2^.

In the Tokyo C group, whole-brain axial DTI scanning was performed on a 3.0 T Philips Achieva scanner using an eight-channel brain coil with the following parameters: two-dimensional diffusion-weighted spin-echo EPI, TR = 5.453 s, TE = 70 ms, acquisition matrix = 112 × 112, reconstruction matrix = 112 × 112, SENSE acceleration factor = 2, FOV = 224 × 224 mm, slice thickness = 2 mm, slice gap = 1 mm, voxel size = 2 × 2 × 3 mm, number of slices = 50. A diffusion sensitization gradient was applied with 32 non-collinear gradient directions and b values of 0 and 1000 s/mm^2^.

In the Toyama B group, whole-brain axial DTI scanning was performed on a 3.0 T Siemens Verio scanner using an 12-channel brain coil with the following parameters: two-dimensional diffusion-weighted spin-echo EPI, TR = 11.5 s, TE = 89 ms, acquisition matrix = 112 × 112, reconstruction matrix = 112 × 112, GRAPPA acceleration factor = 2, FOV = 224 × 224 mm, slice thickness = 2 mm, voxel size = 2 × 2 × 2 mm, number of slices = 75. A diffusion sensitization gradient was applied with 30 non-collinear gradient directions and b values of 0 and 1000 s/mm^2^.

In the Kyushu group, whole-brain axial DTI scanning was performed on a 3.0 T Siemens Spectra scanner using an 16-channel brain coil with the following parameters: two-dimensional diffusion-weighted spin-echo EPI, TR = 12.3 s, TE = 101 ms, acquisition matrix = 112 × 112, reconstruction matrix = 112 × 112, GRAPPA acceleration factor = 2, FOV = 224 × 224 mm, slice thickness = 2 mm, slice gap = 2 mm, voxel size = 2 × 2 × 4 mm, number of slices = 38. A diffusion sensitization gradient was applied with 30 non-collinear gradient directions and b values of 0 and 1000 s/mm^2^.**Supplementary Method 3** Variability ratio of the DTI indices

We calculated the relative variability of the DTI indices of patients compared with those of healthy comparison subjects, as indexed by the log variability ratio (lnVR), the natural logarithm of the ratio of unbiased estimates of the population SDs for each protocol as follows:

lnVR = ln(*σ̂_p_*/*σ̂_c_*) = ln(*s_p_*/*s_c_*) + 1/2(*n_p_* – 1) – 1/2(*n_c_* – 1)

where *σ̂_p_* and *σ̂_c_* are unbiased estimates of population SDs, *s_p_* and *s_c_* are the reported sample SDs, and *n_p_* and *n_c_* are the sample sizes for patient and comparison groups, respectively, in each protocol. For the log variability ratio, the sampling variance *S^2^_lnVR_* is given by:

*S^2^_lnVR_* = 1/2(*n_p_* – 1) + 1/2(*n_c_* – 1)

where *n_p_* and *n_c_* are the sample sizes for the patient and comparison groups, respectively, in each protocol. Then, we performed mega-analyses across protocols with the log variability ratio and that of variance using Metasoft software (http://genetics.cs.ucla.edu/meta).

**SUPPLEMENTARY TABLES**

**Supplementary Table 1** Basic characteristics of the included protocols in comparison among the patient groups.

| Protocol name | Individuals with schizophrenia | | | | |  | Individuals with bipolar disorder | | | | |
| --- | --- | --- | --- | --- | --- | --- | --- | --- | --- | --- | --- |
|  |  |  |  | Age | |  |  |  |  | Age | |
|  | N | Male | Female | Mean | *s.d.* |  | N | Male | Female | Mean | *s.d.* |
| 01. Hokkaido A | 92 | 36 | 56 | 36.0 | 13.6 |  | 77 | 41 | 36 | 44.8 | 14.6 |
| 02. Kanazawa | 111 | 44 | 67 | 39.9 | 12.5 |  | 35 | 18 | 17 | 46.0 | 15.0 |
| 03. Osaka B | 87 | 40 | 47 | 34.0 | 12.9 |  | 5 | 4 | 1 | 41.8 | 17.2 |
| 04. Nagoya | 53 | 29 | 24 | 43.0 | 9.9 |  | 22 | 9 | 13 | 50.3 | 13.8 |
| 05. Tokyo D | 16 | 10 | 6 | 29.4 | 9.1 |  | 15 | 7 | 8 | 32.5 | 7.7 |
| Total | 359 | 159 | 200 | 37.5 | 12.9 |  | 154 | 79 | 75 | 44.6 | 14.7 |
|  | Individuals with schizophrenia | | | | |  | Individuals with autism spectrum disorder | | | | |
| 01. Osaka B | 87 | 40 | 47 | 34.0 | 12.9 |  | 26 | 16 | 10 | 27.2 | 8.9 |
| 02. Osaka A | 69 | 35 | 34 | 34.6 | 12.4 |  | 12 | 8 | 4 | 24.8 | 10.1 |
| 03. Nagoya | 53 | 29 | 24 | 43.0 | 9.9 |  | 13 | 13 | 0 | 31.3 | 9.4 |
| Total | 209 | 104 | 105 | 36.5 | 12.6 |  | 51 | 37 | 14 | 27.7 | 9.4 |
|  | Individuals with schizophrenia | | | | |  | Individuals with major depressive disorder | | | | |
| 01. Hokkaido A | 92 | 36 | 56 | 36.0 | 13.6 |  | 163 | 81 | 82 | 47.9 | 17.5 |
| 02. Kanazawa | 111 | 44 | 67 | 39.9 | 12.5 |  | 43 | 27 | 16 | 43.2 | 13.8 |
| 03. Osaka B | 87 | 40 | 47 | 34.0 | 12.9 |  | 16 | 5 | 11 | 49.1 | 14.9 |
| 04. Osaka A | 69 | 35 | 34 | 34.6 | 12.4 |  | 5 | 3 | 2 | 48.6 | 20.1 |
| 05. Tokyo D | 16 | 10 | 6 | 29.4 | 9.1 |  | 30 | 12 | 18 | 37.1 | 11.0 |
| Total | 375 | 165 | 210 | 36.1 | 12.9 |  | 257 | 128 | 129 | 46.0 | 16.5 |
|  | Individuals with bipolar disorder | | | | |  | Individuals with autism spectrum disorder | | | | |
| 01. Nagoya | 22 | 9 | 13 | 50.3 | 13.8 |  | 13 | 13 | 0 | 31.3 | 9.4 |
| 02. Osaka B | 5 | 4 | 1 | 41.8 | 17.2 |  | 26 | 16 | 10 | 27.2 | 8.9 |
| Total | 27 | 13 | 14 | 48.7 | 14.5 |  | 39 | 29 | 10 | 28.6 | 9.2 |
|  | Individuals with bipolar disorder | | | | |  | Individuals with major depressive disorder | | | | |
| 01. Hokkaido A | 77 | 41 | 36 | 44.8 | 14.6 |  | 163 | 81 | 82 | 47.9 | 17.5 |
| 02. Hiroshima | 39 | 16 | 23 | 52.2 | 13.6 |  | 84 | 31 | 53 | 50.0 | 13.8 |
| 03. Kanazawa | 35 | 18 | 17 | 46.0 | 15.0 |  | 43 | 27 | 16 | 43.2 | 13.8 |
| 04. Yamaguchi | 18 | 10 | 8 | 41.7 | 12.5 |  | 57 | 24 | 33 | 51.7 | 12.5 |
| 05. Tokyo D | 15 | 7 | 8 | 32.5 | 7.7 |  | 30 | 12 | 18 | 37.1 | 11.0 |
| 06. Osaka B | 5 | 4 | 1 | 41.8 | 17.2 |  | 16 | 5 | 11 | 49.1 | 14.9 |
| Total | 189 | 96 | 93 | 45.2 | 14.6 |  | 393 | 180 | 213 | 47.6 | 15.5 |
|  | Individuals with autism spectrum disorder | | | | |  | Individuals with major depressive disorder | | | | |
| 01. Osaka B | 26 | 16 | 10 | 27.2 | 8.9 |  | 16 | 5 | 11 | 49.1 | 14.9 |
| 02. Osaka A | 12 | 8 | 4 | 24.8 | 10.1 |  | 5 | 3 | 2 | 48.6 | 20.1 |
| Total | 38 | 24 | 14 | 26.4 | 9.2 |  | 21 | 8 | 13 | 49.0 | 15.8 |

**Supplementary Table 2** Mega-analysis results of variability ratios (VR) of fractional anisotropy (FA) between patients with schizophrenia and healthy comparison subjects.

| Region of interest | lnVR | lnVR var | VR | *p* | *I^2^* (%) |
| --- | --- | --- | --- | --- | --- |
| ACR | 0.091 | 0.029 | 1.09 | **1.5 × 10^–3^** | 97.3 |
| ALIC | 0.061 | 0.044 | 1.06 | 0.17 | 98.9 |
| Average FA | 0.132 | 0.046 | 1.14 | 3.9 × 10^–3^ | 99.0 |
| BCC | 0.181 | 0.057 | 1.20 | **1.5 × 10^–3^** | 99.4 |
| CC | 0.161 | 0.051 | 1.17 | **1.6 × 10^–3^** | 99.2 |
| CGC | 0.157 | 0.042 | 1.17 | **1.6 × 10^–4^** | 98.8 |
| CGH | 0.092 | 0.044 | 1.10 | 3.5 × 10^–2^ | 98.9 |
| CR | 0.137 | 0.043 | 1.15 | **1.5 × 10^–3^** | 98.9 |
| CST | 0.007 | 0.031 | 1.01 | 0.81 | 97.8 |
| EC | 0.075 | 0.034 | 1.08 | 2.9 × 10^–2^ | 98.2 |
| FX | 0.158 | 0.030 | 1.17 | **9.0 × 10^–8^** | 97.5 |
| FXST | 0.078 | 0.056 | 1.08 | 0.16 | 99.4 |
| GCC | 0.104 | 0.032 | 1.11 | **1.3 × 10^–3^** | 97.9 |
| IC | 0.115 | 0.047 | 1.12 | 1.4 × 10^–2^ | 99.1 |
| IFO | 0.064 | 0.038 | 1.07 | 9.2 × 10^–2^ | 98.6 |
| PCR | 0.112 | 0.047 | 1.12 | 1.7 × 10^–2^ | 99.1 |
| PLIC | 0.132 | 0.046 | 1.14 | 4.4 × 10^–3^ | 99.1 |
| PTR | 0.115 | 0.034 | 1.12 | **8.5 × 10^–4^** | 98.2 |
| RLIC | 0.078 | 0.041 | 1.08 | 5.5 × 10^–2^ | 98.8 |
| SCC | 0.098 | 0.044 | 1.10 | 2.6 × 10^–2^ | 98.9 |
| SCR | 0.153 | 0.036 | 1.17 | **2.8 × 10^–5^** | 98.4 |
| SFO | 0.085 | 0.028 | 1.09 | 2.5 × 10^–3^ | 97.2 |
| SLF | 0.121 | 0.034 | 1.13 | **3.9 × 10^–4^** | 98.2 |
| SS | 0.052 | 0.038 | 1.05 | 0.18 | 98.6 |
| UNC | 0.054 | 0.036 | 1.06 | 0.13 | 98.4 |

**Supplementary Table 3** Mega-analysis results of variability ratios (VR) of mean diffusivity (MD) between patients with schizophrenia and healthy comparison subjects.

| Region of interest | lnVR | lnVR var | VR | *p* | *I^2^* (%) |
| --- | --- | --- | --- | --- | --- |
| ACR | 0.269 | 0.056 | 1.31 | **1.7 × 10^–6^** | 99.4 |
| ALIC | 0.089 | 0.061 | 1.09 | 0.15 | 99.5 |
| Average MD | 0.121 | 0.033 | 1.13 | **2.5 × 10^–4^** | 98.0 |
| BCC | 0.216 | 0.032 | 1.24 | **2.4 × 10^–11^** | 98.0 |
| CC | 0.272 | 0.045 | 1.31 | **1.8 × 10^–9^** | 99.0 |
| CGC | 0.145 | 0.038 | 1.16 | **1.6 × 10^–4^** | 98.6 |
| CGH | 0.087 | 0.043 | 1.09 | 4.3 × 10^–2^ | 98.9 |
| CR | 0.235 | 0.054 | 1.27 | **1.5 × 10^–5^** | 99.3 |
| CST | 0.139 | 0.046 | 1.15 | 2.3 × 10^–3^ | 99.0 |
| EC | 0.069 | 0.040 | 1.07 | 8.4 × 10^–2^ | 98.7 |
| FX | 0.167 | 0.054 | 1.18 | 2.2 × 10^–3^ | 99.3 |
| FXST | 0.051 | 0.053 | 1.05 | 0.34 | 99.3 |
| GCC | 0.368 | 0.072 | 1.45 | **2.8 × 10^–7^** | 99.6 |
| IC | 0.131 | 0.049 | 1.14 | 7.6 × 10^–3^ | 99.2 |
| IFO | 0.247 | 0.053 | 1.28 | **3.4 × 10^–6^** | 99.3 |
| PCR | 0.055 | 0.061 | 1.06 | 0.37 | 99.5 |
| PLIC | 0.110 | 0.052 | 1.12 | 3.7 × 10^–2^ | 99.3 |
| PTR | 0.059 | 0.070 | 1.06 | 0.40 | 99.6 |
| RLIC | 0.100 | 0.036 | 1.10 | 5.3 × 10^–3^ | 98.4 |
| SCC | 0.089 | 0.045 | 1.09 | 4.7 × 10^–2^ | 99.0 |
| SCR | 0.215 | 0.064 | 1.24 | **8.3 × 10^–4^** | 99.5 |
| SFO | 0.202 | 0.087 | 1.22 | 2.0 × 10^–2^ | 99.7 |
| SLF | 0.108 | 0.040 | 1.11 | 7.0 × 10^–3^ | 98.7 |
| SS | 0.104 | 0.035 | 1.11 | 2.7 × 10^–3^ | 98.2 |
| UNC | 0.156 | 0.128 | 1.17 | 0.22 | 99.9 |

**Supplementary Table 4** Mega-analysis results of variability ratios (VR) in axial diffusivity (AD) between patients with schizophrenia and healthy comparison subjects.

| Region of interest | lnVR | lnVR var | VR | *p* | *I^2^* (%) |
| --- | --- | --- | --- | --- | --- |
| ACR | 0.158 | 0.040 | 1.17 | **7.4 × 10^–5^** | 98.7 |
| ALIC | 0.179 | 0.049 | 1.20 | **2.4 × 10^–4^** | 99.2 |
| Average AD | 0.160 | 0.046 | 1.17 | **5.0 × 10^–4^** | 99.0 |
| BCC | 0.116 | 0.021 | 1.12 | **5.6 × 10^–8^** | 94.9 |
| CC | 0.103 | 0.030 | 1.11 | **5.3 × 10^–4^** | 97.6 |
| CGC | 0.084 | 0.033 | 1.09 | 1.1 × 10^–2^ | 98.0 |
| CGH | 0.095 | 0.043 | 1.10 | 2.7 × 10^–2^ | 98.9 |
| CR | 0.188 | 0.046 | 1.21 | **5.1 × 10^–5^** | 99.1 |
| CST | 0.126 | 0.055 | 1.13 | 2.1 × 10^–2^ | 99.3 |
| EC | 0.127 | 0.034 | 1.14 | **1.9 × 10^–4^** | 98.2 |
| FX | 0.100 | 0.044 | 1.11 | 2.4 × 10^–2^ | 99.0 |
| FXST | 0.093 | 0.029 | 1.10 | **1.3 × 10^–3^** | 97.4 |
| GCC | 0.073 | 0.034 | 1.08 | 3.0 × 10^–2^ | 98.1 |
| IC | 0.160 | 0.045 | 1.17 | **3.5 × 10^–4^** | 99.0 |
| IFO | 0.106 | 0.037 | 1.11 | 4.6 × 10^–3^ | 98.5 |
| PCR | 0.061 | 0.044 | 1.06 | 0.17 | 99.0 |
| PLIC | 0.181 | 0.044 | 1.20 | **4.4 × 10^–5^** | 99.0 |
| PTR | 0.027 | 0.036 | 1.03 | 0.45 | 98.4 |
| RLIC | 0.056 | 0.042 | 1.06 | 0.18 | 98.8 |
| SCC | 0.054 | 0.048 | 1.06 | 0.26 | 99.1 |
| SCR | 0.208 | 0.052 | 1.23 | **5.4 × 10^–5^** | 99.2 |
| SFO | 0.250 | 0.081 | 1.28 | **1.9 × 10^–3^** | 99.7 |
| SLF | 0.117 | 0.035 | 1.12 | **7.2 × 10^–4^** | 98.2 |
| SS | 0.024 | 0.032 | 1.02 | 0.45 | 97.9 |
| UNC | 0.043 | 0.063 | 1.04 | 0.50 | 99.5 |

**Supplementary Table 5** Mega-analysis results of variability ratios (VR) in radial diffusivity (RD) between patients with schizophrenia and healthy comparison subjects.

| Region of interest | lnVR | lnVR var | VR | *p* | *I^2^* (%) |
| --- | --- | --- | --- | --- | --- |
| ACR | 0.234 | 0.056 | 1.26 | **2.7 × 10^–5^** | 99.4 |
| ALIC | 0.049 | 0.057 | 1.05 | 0.39 | 99.4 |
| Average RD | 0.139 | 0.033 | 1.15 | **3.0 × 10^–5^** | 98.1 |
| BCC | 0.250 | 0.043 | 1.28 | **8.9 × 10^–9^** | 98.9 |
| CC | 0.255 | 0.058 | 1.29 | **1.3 × 10^–5^** | 99.4 |
| CGC | 0.157 | 0.041 | 1.17 | **1.3 × 10^–4^** | 98.8 |
| CGH | 0.105 | 0.046 | 1.11 | 2.1 × 10^–2^ | 99.0 |
| CR | 0.210 | 0.055 | 1.23 | **1.2 × 10^–4^** | 99.3 |
| CST | 0.118 | 0.045 | 1.12 | 9.4 × 10^–3^ | 99.0 |
| EC | 0.078 | 0.029 | 1.08 | 7.3 × 10^–3^ | 97.4 |
| FX | 0.201 | 0.058 | 1.22 | **5.5 × 10^–4^** | 99.4 |
| FXST | 0.073 | 0.051 | 1.08 | 0.15 | 99.2 |
| GCC | 0.257 | 0.069 | 1.29 | **1.8 × 10^–4^** | 99.6 |
| IC | 0.086 | 0.051 | 1.09 | 9.3 × 10^–2^ | 99.2 |
| IFO | 0.201 | 0.038 | 1.22 | **1.7 × 10^–7^** | 98.6 |
| PCR | 0.062 | 0.078 | 1.06 | 0.43 | 99.7 |
| PLIC | 0.127 | 0.049 | 1.14 | 1.0 × 10^–2^ | 99.2 |
| PTR | 0.128 | 0.067 | 1.14 | 5.6 × 10^–2^ | 99.6 |
| RLIC | 0.103 | 0.039 | 1.11 | 8.2 × 10^–3^ | 98.6 |
| SCC | 0.116 | 0.049 | 1.12 | 1.9 × 10^–2^ | 99.2 |
| SCR | 0.198 | 0.046 | 1.22 | **1.5 × 10^–5^** | 99.0 |
| SFO | 0.132 | 0.084 | 1.14 | 0.12 | 99.7 |
| SLF | 0.138 | 0.037 | 1.15 | **2.2 × 10^–4^** | 98.5 |
| SS | 0.072 | 0.039 | 1.07 | 6.6 × 10^–2^ | 98.6 |
| UNC | 0.172 | 0.114 | 1.19 | 0.13 | 99.9 |

**Supplementary Table 6** Mega-analysis results of variability ratios (VR) in fractional anisotropy (FA) between patients with bipolar disorder and healthy comparison subjects.

| Region of interest | lnVR | lnVR var | VR | *p* | *I^2^* (%) |
| --- | --- | --- | --- | --- | --- |
| ACR | 0.072 | 0.077 | 1.07 | 0.35 | 97.7 |
| ALIC | 0.202 | 0.133 | 1.22 | 0.13 | 99.2 |
| Average FA | 0.165 | 0.123 | 1.18 | 0.18 | 99.1 |
| BCC | 0.103 | 0.129 | 1.11 | 0.42 | 99.2 |
| CC | 0.120 | 0.123 | 1.13 | 0.33 | 99.1 |
| CGC | 0.171 | 0.109 | 1.19 | 0.12 | 98.9 |
| CGH | 0.132 | 0.062 | 1.14 | 3.3 × 10^–2^ | 96.4 |
| CR | 0.057 | 0.081 | 1.06 | 0.48 | 97.9 |
| CST | 0.080 | 0.074 | 1.08 | 0.28 | 97.5 |
| EC | 0.013 | 0.065 | 1.01 | 0.84 | 96.7 |
| FX | 0.082 | 0.105 | 1.09 | 0.43 | 98.8 |
| FXST | –0.350 | 0.091 | 0.70 | **1.2 × 10^–4^** | 98.4 |
| GCC | 0.102 | 0.112 | 1.11 | 0.36 | 98.9 |
| IC | 0.045 | 0.073 | 1.05 | 0.54 | 97.4 |
| IFO | –0.081 | 0.036 | 0.92 | 2.4 × 10^–2^ | 88.6 |
| PCR | –0.102 | 0.091 | 0.90 | 0.26 | 98.4 |
| PLIC | 0.022 | 0.076 | 1.02 | 0.78 | 97.6 |
| PTR | –0.062 | 0.069 | 0.94 | 0.37 | 97.0 |
| RLIC | –0.109 | 0.059 | 0.90 | 6.6 × 10^–2^ | 96.0 |
| SCC | 0.141 | 0.119 | 1.15 | 0.24 | 99.1 |
| SCR | 0.115 | 0.060 | 1.12 | 5.3 × 10^–2^ | 96.0 |
| SFO | 0.156 | 0.070 | 1.17 | 2.6 × 10^–2^ | 97.2 |
| SLF | 0.127 | 0.091 | 1.14 | 0.16 | 98.4 |
| SS | –0.026 | 0.095 | 0.97 | 0.78 | 98.5 |
| UNC | 0.049 | 0.075 | 1.05 | 0.51 | 97.5 |

**Supplementary Table 7** Mega-analysis results of variability ratios (VR) in mean diffusivity (MD) between patients with bipolar disorder and healthy comparison subjects.

| Region of interest | lnVR | lnVR var | VR | *p* | *I^2^* (%) |
| --- | --- | --- | --- | --- | --- |
| ACR | 0.253 | 0.133 | 1.29 | 5.7 × 10^–2^ | 99.2 |
| ALIC | 0.283 | 0.173 | 1.33 | 0.10 | 99.6 |
| Average MD | 0.254 | 0.106 | 1.29 | 1.6 × 10^–2^ | 98.8 |
| BCC | 0.237 | 0.149 | 1.27 | 0.11 | 99.4 |
| CC | 0.265 | 0.168 | 1.30 | 0.11 | 99.5 |
| CGC | 0.080 | 0.095 | 1.08 | 0.40 | 98.5 |
| CGH | 0.066 | 0.067 | 1.07 | 0.32 | 96.9 |
| CR | 0.288 | 0.138 | 1.33 | 3.7 × 10^–2^ | 99.3 |
| CST | 0.135 | 0.114 | 1.14 | 0.23 | 99.0 |
| EC | 0.265 | 0.134 | 1.30 | 4.8 × 10^–2^ | 99.3 |
| FX | 0.099 | 0.105 | 1.10 | 0.35 | 98.8 |
| FXST | 0.083 | 0.128 | 1.09 | 0.52 | 99.2 |
| GCC | 0.300 | 0.146 | 1.35 | 4.1 × 10^–2^ | 99.4 |
| IC | 0.199 | 0.117 | 1.22 | 8.9 × 10^–2^ | 99.0 |
| IFO | 0.036 | 0.199 | 1.04 | 0.86 | 99.7 |
| PCR | 0.180 | 0.189 | 1.20 | 0.34 | 99.6 |
| PLIC | 0.100 | 0.074 | 1.11 | 0.18 | 97.5 |
| PTR | 0.032 | 0.105 | 1.03 | 0.76 | 98.8 |
| RLIC | 0.194 | 0.127 | 1.21 | 0.13 | 99.2 |
| SCC | 0.046 | 0.149 | 1.05 | 0.76 | 99.4 |
| SCR | 0.317 | 0.135 | 1.37 | 1.8 × 10^–2^ | 99.3 |
| SFO | 0.257 | 0.121 | 1.29 | 3.4 × 10^–2^ | 99.1 |
| SLF | 0.184 | 0.115 | 1.20 | 0.11 | 99.0 |
| SS | 0.211 | 0.111 | 1.23 | 5.7 × 10^–2^ | 98.9 |
| UNC | 0.099 | 0.211 | 1.10 | 0.64 | 99.7 |

**Supplementary Table 8** Mega-analysis results of variability ratios (VR) in axial diffusivity (AD) between patients with bipolar disorder and healthy comparison subjects.

| Region of interest | lnVR | lnVR var | VR | *p* | *I^2^* (%) |
| --- | --- | --- | --- | --- | --- |
| ACR | 0.103 | 0.102 | 1.11 | 0.31 | 98.7 |
| ALIC | 0.144 | 0.110 | 1.16 | 0.19 | 98.9 |
| Average AD | 0.129 | 0.053 | 1.14 | 1.4 × 10^–2^ | 94.9 |
| BCC | 0.047 | 0.051 | 1.05 | 0.35 | 94.4 |
| CC | 0.147 | 0.083 | 1.16 | 7.7 × 10^–2^ | 98.0 |
| CGC | –0.024 | 0.062 | 0.98 | 0.70 | 96.4 |
| CGH | 0.093 | 0.073 | 1.10 | 0.20 | 97.4 |
| CR | 0.293 | 0.112 | 1.34 | 8.9 × 10^–3^ | 98.9 |
| CST | 0.062 | 0.102 | 1.06 | 0.54 | 98.7 |
| EC | 0.166 | 0.125 | 1.18 | 0.18 | 99.1 |
| FX | 0.028 | 0.093 | 1.03 | 0.76 | 98.4 |
| FXST | 0.085 | 0.106 | 1.09 | 0.43 | 98.8 |
| GCC | 0.124 | 0.137 | 1.13 | 0.37 | 99.3 |
| IC | 0.149 | 0.090 | 1.16 | 0.10 | 98.3 |
| IFO | 0.102 | 0.129 | 1.11 | 0.43 | 99.2 |
| PCR | 0.274 | 0.144 | 1.31 | 5.6 × 10^–2^ | 99.4 |
| PLIC | 0.100 | 0.050 | 1.11 | 4.6 × 10^–2^ | 94.4 |
| PTR | 0.028 | 0.136 | 1.03 | 0.84 | 99.3 |
| RLIC | 0.203 | 0.105 | 1.23 | 5.2 × 10^–2^ | 98.8 |
| SCC | 0.114 | 0.074 | 1.12 | 0.12 | 97.5 |
| SCR | 0.347 | 0.110 | 1.42 | **1.6 × 10^–3^** | 98.9 |
| SFO | 0.197 | 0.108 | 1.22 | 6.7 × 10^–2^ | 98.8 |
| SLF | 0.096 | 0.096 | 1.10 | 0.32 | 98.5 |
| SS | 0.080 | 0.091 | 1.08 | 0.38 | 98.4 |
| UNC | 0.180 | 0.134 | 1.20 | 0.18 | 99.3 |

**Supplementary Table 9** Mega-analysis results of variability ratios (VR) in radial diffusivity (RD) between patients with bipolar disorder and healthy comparison subjects.

| Region of interest | lnVR | lnVR var | VR | *p* | *I^2^* (%) |
| --- | --- | --- | --- | --- | --- |
| ACR | 0.210 | 0.118 | 1.23 | 7.5 × 10^–2^ | 99.0 |
| ALIC | 0.330 | 0.187 | 1.39 | 7.8 × 10^–2^ | 99.6 |
| Average RD | 0.290 | 0.128 | 1.34 | 2.4 × 10^–2^ | 99.2 |
| BCC | 0.230 | 0.167 | 1.26 | 0.17 | 99.5 |
| CC | 0.250 | 0.176 | 1.28 | 0.15 | 99.6 |
| CGC | 0.180 | 0.105 | 1.20 | 8.4 × 10^–2^ | 98.8 |
| CGH | 0.088 | 0.053 | 1.09 | 0.10 | 95.0 |
| CR | 0.214 | 0.125 | 1.24 | 8.7 × 10^–2^ | 99.1 |
| CST | 0.200 | 0.130 | 1.22 | 0.12 | 99.2 |
| EC | 0.222 | 0.121 | 1.25 | 6.6 × 10^–2^ | 99.1 |
| FX | 0.131 | 0.108 | 1.14 | 0.22 | 98.8 |
| FXST | –0.215 | 0.126 | 0.81 | 8.9 × 10^–2^ | 99.2 |
| GCC | 0.264 | 0.145 | 1.30 | 6.9 × 10^–2^ | 99.4 |
| IC | 0.133 | 0.117 | 1.14 | 0.26 | 99.0 |
| IFO | –0.024 | 0.131 | 0.98 | 0.86 | 99.2 |
| PCR | 0.045 | 0.195 | 1.05 | 0.82 | 99.7 |
| PLIC | 0.070 | 0.079 | 1.07 | 0.38 | 97.8 |
| PTR | –0.011 | 0.102 | 0.99 | 0.92 | 98.7 |
| RLIC | 0.053 | 0.090 | 1.05 | 0.56 | 98.3 |
| SCC | 0.141 | 0.177 | 1.15 | 0.43 | 99.6 |
| SCR | 0.219 | 0.107 | 1.24 | 4.0 × 10^–2^ | 98.8 |
| SFO | 0.253 | 0.126 | 1.29 | 4.4 × 10^–2^ | 99.2 |
| SLF | 0.196 | 0.120 | 1.22 | 0.10 | 99.1 |
| SS | 0.148 | 0.102 | 1.16 | 0.15 | 98.7 |
| UNC | 0.065 | 0.213 | 1.07 | 0.76 | 99.7 |

**Supplementary Table 10** Mega-analysis results of variability ratios (VR) in fractional anisotropy (FA) between individuals with autism spectrum disorder and healthy comparison subjects.

| Region of interest | lnVR | lnVR var | VR | *p* | *I^2^* (%) |
| --- | --- | --- | --- | --- | --- |
| ACR | 0.178 | 0.093 | 1.19 | 5.5 × 10^–2^ | 98.0 |
| ALIC | 0.094 | 0.090 | 1.10 | 0.29 | 97.9 |
| Average FA | 0.060 | 0.049 | 1.06 | 0.22 | 92.5 |
| BCC | –0.042 | 0.075 | 0.96 | 0.57 | 96.9 |
| CC | –0.051 | 0.070 | 0.95 | 0.46 | 96.4 |
| CGC | 0.068 | 0.046 | 1.07 | 0.14 | 91.5 |
| CGH | –0.114 | 0.125 | 0.89 | 0.36 | 98.9 |
| CR | 0.123 | 0.128 | 1.13 | 0.34 | 99.0 |
| CST | 0.006 | 0.136 | 1.01 | 0.97 | 99.1 |
| EC | 0.069 | 0.128 | 1.07 | 0.59 | 98.9 |
| FX | –0.088 | 0.058 | 0.92 | 0.13 | 94.8 |
| FXST | –0.008 | 0.084 | 0.99 | 0.93 | 97.6 |
| GCC | 3.3 × 10^–4^ | 0.071 | 1.00 | 1.00 | 96.5 |
| IC | 0.097 | 0.068 | 1.10 | 0.16 | 96.2 |
| IFO | –0.077 | 0.091 | 0.93 | 0.40 | 97.9 |
| PCR | 0.137 | 0.106 | 1.15 | 0.20 | 98.5 |
| PLIC | –0.004 | 0.074 | 1.00 | 0.95 | 96.8 |
| PTR | 0.176 | 0.105 | 1.19 | 9.3 × 10^–2^ | 98.4 |
| RLIC | 0.135 | 0.057 | 1.14 | 1.8 × 10^–2^ | 94.6 |
| SCC | –0.044 | 0.032 | 0.96 | 0.16 | 81.5 |
| SCR | 0.070 | 0.110 | 1.07 | 0.52 | 98.6 |
| SFO | –0.029 | 0.034 | 0.97 | 0.39 | 83.7 |
| SLF | 0.199 | 0.111 | 1.22 | 7.4 × 10^–2^ | 98.6 |
| SS | 0.048 | 0.090 | 1.05 | 0.59 | 97.9 |
| UNC | –0.035 | 0.047 | 0.97 | 0.46 | 91.9 |

**Supplementary Table 11** Mega-analysis results of variability ratios (VR) in mean diffusivity (MD) between individuals with autism spectrum disorder and healthy comparison subjects.

| Region of interest | lnVR | lnVR var | VR | *p* | *I^2^* (%) |
| --- | --- | --- | --- | --- | --- |
| ACR | 0.121 | 0.146 | 1.13 | 0.41 | 99.2 |
| ALIC | –0.144 | 0.196 | 0.87 | 0.46 | 99.6 |
| Average MD | 0.023 | 0.082 | 1.02 | 0.78 | 97.4 |
| BCC | 0.065 | 0.088 | 1.07 | 0.46 | 97.8 |
| CC | 0.036 | 0.094 | 1.04 | 0.70 | 98.0 |
| CGC | 0.074 | 0.090 | 1.08 | 0.41 | 97.9 |
| CGH | –0.026 | 0.104 | 0.97 | 0.80 | 98.4 |
| CR | 0.095 | 0.136 | 1.10 | 0.48 | 99.1 |
| CST | 0.100 | 0.081 | 1.11 | 0.22 | 97.4 |
| EC | 0.048 | 0.144 | 1.05 | 0.74 | 99.2 |
| FX | –0.038 | 0.129 | 0.96 | 0.77 | 99.0 |
| FXST | 0.007 | 0.120 | 1.01 | 0.95 | 98.8 |
| GCC | 0.012 | 0.154 | 1.01 | 0.94 | 99.3 |
| IC | –0.044 | 0.121 | 0.96 | 0.72 | 98.8 |
| IFO | –0.085 | 0.074 | 0.92 | 0.25 | 96.8 |
| PCR | 0.095 | 0.065 | 1.10 | 0.14 | 95.9 |
| PLIC | –0.046 | 0.078 | 0.96 | 0.56 | 97.2 |
| PTR | 0.147 | 0.057 | 1.16 | 1.0 × 10^–2^ | 94.6 |
| RLIC | 0.121 | 0.050 | 1.13 | 1.6 × 10^–2^ | 92.8 |
| SCC | 0.072 | 0.052 | 1.07 | 0.17 | 93.5 |
| SCR | 0.013 | 0.117 | 1.01 | 0.91 | 98.7 |
| SFO | –0.007 | 0.122 | 0.99 | 0.95 | 98.8 |
| SLF | 0.051 | 0.084 | 1.05 | 0.55 | 97.5 |
| SS | 0.049 | 0.075 | 1.05 | 0.51 | 96.9 |
| UNC | 0.049 | 0.026 | 1.05 | 6.1 × 10^–2^ | 72.3 |

**Supplementary Table 12** Mega-analysis results of variability ratios (VR) in axial diffusivity (AD) between individuals with autism spectrum disorder and healthy comparison subjects.

| Region of interest | lnVR | lnVR var | VR | *p* | *I^2^* (%) |
| --- | --- | --- | --- | --- | --- |
| ACR | 0.075 | 0.143 | 1.08 | 0.60 | 99.2 |
| ALIC | 0.009 | 0.182 | 1.01 | 0.96 | 99.5 |
| Average AD | –0.064 | 0.191 | 0.94 | 0.74 | 99.5 |
| BCC | –0.005 | 0.090 | 0.99 | 0.95 | 97.8 |
| CC | –0.092 | 0.061 | 0.91 | 0.13 | 95.3 |
| CGC | 0.029 | 0.086 | 1.03 | 0.73 | 97.7 |
| CGH | –0.131 | 0.143 | 0.88 | 0.36 | 99.2 |
| CR | –4.3 × 10^–5^ | 0.180 | 1.00 | 1.00 | 99.5 |
| CST | 0.139 | 0.048 | 1.15 | 4.2 × 10^–3^ | 92.3 |
| EC | –0.107 | 0.131 | 0.90 | 0.41 | 99.0 |
| FX | 0.013 | 0.130 | 1.01 | 0.92 | 99.0 |
| FXST | –0.076 | 0.130 | 0.93 | 0.56 | 99.0 |
| GCC | –0.188 | 0.124 | 0.83 | 0.13 | 98.9 |
| IC | 0.087 | 0.168 | 1.09 | 0.60 | 99.4 |
| IFO | –0.060 | 0.092 | 0.94 | 0.51 | 98.0 |
| PCR | –0.006 | 0.055 | 0.99 | 0.91 | 94.2 |
| PLIC | 0.117 | 0.083 | 1.12 | 0.16 | 97.5 |
| PTR | 0.024 | 0.040 | 1.02 | 0.55 | 88.3 |
| RLIC | 0.166 | 0.118 | 1.18 | 0.16 | 98.8 |
| SCC | –0.164 | 0.081 | 0.85 | 4.2 × 10^–2^ | 97.3 |
| SCR | 0.059 | 0.106 | 1.06 | 0.58 | 98.5 |
| SFO | –0.060 | 0.082 | 0.94 | 0.47 | 97.4 |
| SLF | –0.029 | 0.140 | 0.97 | 0.83 | 99.1 |
| SS | –0.023 | 0.077 | 0.98 | 0.76 | 97.1 |
| UNC | –0.021 | 0.091 | 0.98 | 0.82 | 97.9 |

**Supplementary Table 13** Mega-analysis results of variability ratios (VR) in radial diffusivity (RD) between individuals with autism spectrum disorder and healthy comparison subjects.

| Region of interest | lnVR | lnVR var | VR | *p* | *I^2^* (%) |
| --- | --- | --- | --- | --- | --- |
| ACR | 0.151 | 0.096 | 1.16 | 0.12 | 98.1 |
| ALIC | 0.009 | 0.106 | 1.01 | 0.93 | 98.5 |
| Average RD | 0.052 | 0.050 | 1.05 | 0.30 | 93.0 |
| BCC | 0.030 | 0.080 | 1.03 | 0.71 | 97.3 |
| CC | 0.041 | 0.069 | 1.04 | 0.55 | 96.3 |
| CGC | 0.121 | 0.060 | 1.13 | 4.3 × 10^–2^ | 95.0 |
| CGH | –0.067 | 0.107 | 0.94 | 0.53 | 98.5 |
| CR | 0.155 | 0.102 | 1.17 | 0.13 | 98.4 |
| CST | –0.058 | 0.067 | 0.94 | 0.38 | 96.0 |
| EC | 0.111 | 0.064 | 1.12 | 8.5 × 10^–2^ | 95.7 |
| FX | –0.061 | 0.126 | 0.94 | 0.63 | 98.9 |
| FXST | –0.082 | 0.117 | 0.92 | 0.48 | 98.7 |
| GCC | 0.063 | 0.094 | 1.06 | 0.50 | 98.1 |
| IC | –0.005 | 0.045 | 1.00 | 0.91 | 91.1 |
| IFO | –0.055 | 0.065 | 0.95 | 0.40 | 95.8 |
| PCR | 0.181 | 0.087 | 1.20 | 3.7 × 10^–2^ | 97.7 |
| PLIC | –0.116 | 0.067 | 0.89 | 8.3 × 10^–2^ | 96.1 |
| PTR | 0.177 | 0.077 | 1.19 | 2.1 × 10^–2^ | 97.1 |
| RLIC | 0.097 | 0.052 | 1.10 | 6.4 × 10^–2^ | 93.5 |
| SCC | 0.109 | 0.020 | 1.12 | **2.6 × 10^–8^** | 51.8 |
| SCR | 0.090 | 0.082 | 1.09 | 0.28 | 97.4 |
| SFO | 0.076 | 0.068 | 1.08 | 0.26 | 96.2 |
| SLF | 0.165 | 0.096 | 1.18 | 8.4 × 10^–2^ | 98.1 |
| SS | 0.046 | 0.083 | 1.05 | 0.58 | 97.5 |
| UNC | 0.026 | 0.059 | 1.03 | 0.66 | 94.9 |

**Supplementary Table 14** Mega-analysis results of variability ratios (VR) in fractional anisotropy (FA) between patients with major depressive disorder and healthy comparison subjects.

| Region of interest | lnVR | lnVR var | VR | *p* | *I^2^* (%) |
| --- | --- | --- | --- | --- | --- |
| ACR | 0.057 | 0.073 | 1.06 | 0.43 | 98.8 |
| ALIC | 0.171 | 0.094 | 1.19 | 6.9 × 10^–2^ | 99.3 |
| Average FA | 0.144 | 0.078 | 1.16 | 6.4 × 10^–2^ | 98.9 |
| BCC | –0.035 | 0.050 | 0.97 | 0.48 | 97.3 |
| CC | –0.023 | 0.052 | 0.98 | 0.66 | 97.5 |
| CGC | 0.036 | 0.079 | 1.04 | 0.65 | 98.9 |
| CGH | 0.115 | 0.043 | 1.12 | 8.1 × 10^–3^ | 96.3 |
| CR | 0.056 | 0.058 | 1.06 | 0.33 | 98.0 |
| CST | 0.195 | 0.058 | 1.22 | **8.4 × 10^–4^** | 98.0 |
| EC | 0.080 | 0.065 | 1.08 | 0.22 | 98.4 |
| FX | 0.011 | 0.064 | 1.01 | 0.87 | 98.4 |
| FXST | 0.067 | 0.040 | 1.07 | 9.2 × 10^–2^ | 95.6 |
| GCC | 0.009 | 0.058 | 1.01 | 0.87 | 98.0 |
| IC | 0.087 | 0.043 | 1.09 | 4.0 × 10^–2^ | 96.2 |
| IFO | 0.086 | 0.053 | 1.09 | 0.11 | 97.6 |
| PCR | –0.081 | 0.039 | 0.92 | 3.6 × 10^–2^ | 95.3 |
| PLIC | 0.076 | 0.060 | 1.08 | 0.21 | 98.2 |
| PTR | –0.069 | 0.045 | 0.93 | 0.12 | 96.5 |
| RLIC | –0.025 | 0.028 | 0.98 | 0.37 | 90.4 |
| SCC | 0.013 | 0.082 | 1.01 | 0.87 | 99.0 |
| SCR | 0.101 | 0.056 | 1.11 | 7.2 × 10^–2^ | 97.9 |
| SFO | 0.122 | 0.057 | 1.13 | 3.2 × 10^–2^ | 97.9 |
| SLF | 0.052 | 0.046 | 1.05 | 0.25 | 96.7 |
| SS | 0.068 | 0.044 | 1.07 | 0.12 | 96.5 |
| UNC | 0.077 | 0.056 | 1.08 | 0.17 | 97.9 |

**Supplementary Table 15** Mega-analysis results of variability ratios (VR) in mean diffusivity (MD) between patients with major depressive disorder and healthy comparison subjects.

| Region of interest | lnVR | lnVR var | VR | *p* | *I^2^* (%) |
| --- | --- | --- | --- | --- | --- |
| ACR | 0.172 | 0.087 | 1.19 | 4.9 × 10^–2^ | 99.2 |
| ALIC | 0.213 | 0.133 | 1.24 | 0.11 | 99.6 |
| Average MD | 0.188 | 0.078 | 1.21 | 1.7 × 10^–2^ | 98.9 |
| BCC | 0.064 | 0.069 | 1.07 | 0.36 | 98.6 |
| CC | 0.100 | 0.085 | 1.10 | 0.24 | 99.1 |
| CGC | 0.040 | 0.058 | 1.04 | 0.49 | 98.1 |
| CGH | 0.070 | 0.069 | 1.07 | 0.31 | 98.6 |
| CR | 0.127 | 0.072 | 1.14 | 7.6 × 10^–2^ | 98.7 |
| CST | 0.290 | 0.042 | 1.34 | **3.3 × 10^–12^** | 96.0 |
| EC | 0.256 | 0.082 | 1.29 | **1.9 × 10^–3^** | 99.0 |
| FX | 0.098 | 0.074 | 1.10 | 0.18 | 98.8 |
| FXST | 0.264 | 0.086 | 1.30 | 2.2 × 10^–3^ | 99.1 |
| GCC | 0.166 | 0.078 | 1.18 | 3.3 × 10^–2^ | 98.9 |
| IC | 0.177 | 0.056 | 1.19 | **1.5 × 10^–3^** | 97.8 |
| IFO | 0.215 | 0.091 | 1.24 | 1.8 × 10^–2^ | 99.2 |
| PCR | 0.049 | 0.065 | 1.05 | 0.45 | 98.4 |
| PLIC | 0.104 | 0.049 | 1.11 | 3.2 × 10^–2^ | 97.1 |
| PTR | 0.124 | 0.075 | 1.13 | 0.10 | 98.8 |
| RLIC | 0.205 | 0.057 | 1.23 | **3.2 × 10^–4^** | 97.9 |
| SCC | 0.068 | 0.061 | 1.07 | 0.27 | 98.2 |
| SCR | 0.171 | 0.071 | 1.19 | 1.6 × 10^–2^ | 98.7 |
| SFO | 0.134 | 0.094 | 1.14 | 0.15 | 99.3 |
| SLF | 0.036 | 0.066 | 1.04 | 0.59 | 98.5 |
| SS | 0.211 | 0.087 | 1.23 | 1.5 × 10^–2^ | 99.1 |
| UNC | 0.167 | 0.095 | 1.18 | 7.8 × 10^–2^ | 99.3 |

**Supplementary Table 16** Mega-analysis results of variability ratios (VR) in axial diffusivity (AD) between patients with major depressive disorder and healthy comparison subjects.

| Region of interest | lnVR | lnVR var | VR | *p* | *I^2^* (%) |
| --- | --- | --- | --- | --- | --- |
| ACR | 0.064 | 0.083 | 1.07 | 0.44 | 99.1 |
| ALIC | 0.184 | 0.105 | 1.20 | 8.1 × 10^–2^ | 99.4 |
| Average AD | 0.107 | 0.062 | 1.11 | 8.4 × 10^–2^ | 98.3 |
| BCC | –0.046 | 0.056 | 0.95 | 0.41 | 97.9 |
| CC | 0.060 | 0.079 | 1.06 | 0.45 | 99.0 |
| CGC | 0.020 | 0.043 | 1.02 | 0.64 | 96.2 |
| CGH | 0.099 | 0.049 | 1.10 | 4.3 × 10^–2^ | 97.2 |
| CR | 0.099 | 0.068 | 1.10 | 0.14 | 98.6 |
| CST | 0.167 | 0.062 | 1.18 | 6.6 × 10^–3^ | 98.3 |
| EC | 0.126 | 0.075 | 1.13 | 9.1 × 10^–2^ | 98.8 |
| FX | 0.081 | 0.073 | 1.08 | 0.26 | 98.8 |
| FXST | 0.021 | 0.102 | 1.02 | 0.84 | 99.4 |
| GCC | 0.121 | 0.053 | 1.13 | 2.3 × 10^–2^ | 97.6 |
| IC | 0.144 | 0.050 | 1.16 | 4.1 × 10^–3^ | 97.3 |
| IFO | 0.001 | 0.078 | 1.00 | 0.99 | 98.9 |
| PCR | 0.073 | 0.040 | 1.08 | 7.2 × 10^–2^ | 95.8 |
| PLIC | 0.080 | 0.035 | 1.08 | 2.1 × 10^–2^ | 94.1 |
| PTR | 0.108 | 0.048 | 1.11 | 2.6 × 10^–2^ | 97.1 |
| RLIC | 0.195 | 0.064 | 1.22 | 2.2 × 10^–3^ | 98.4 |
| SCC | 0.077 | 0.060 | 1.08 | 0.20 | 98.2 |
| SCR | 0.155 | 0.072 | 1.17 | 3.1 × 10^–2^ | 98.7 |
| SFO | 0.207 | 0.082 | 1.23 | 1.2 × 10^–2^ | 99.0 |
| SLF | 0.009 | 0.054 | 1.01 | 0.86 | 97.7 |
| SS | 0.152 | 0.091 | 1.16 | 9.4 × 10^–2^ | 99.2 |
| UNC | 0.077 | 0.037 | 1.08 | 3.7 × 10^–2^ | 94.8 |

**Supplementary Table 17** Mega-analysis results of variability ratios (VR) in radial diffusivity (RD) between patients with major depressive disorder and healthy comparison subjects.

| Region of interest | lnVR | lnVR var | VR | *p* | *I^2^* (%) |
| --- | --- | --- | --- | --- | --- |
| ACR | 0.179 | 0.069 | 1.20 | 9.6 × 10^–3^ | 98.6 |
| ALIC | 0.197 | 0.139 | 1.22 | 0.16 | 99.7 |
| Average RD | 0.243 | 0.089 | 1.27 | 6.6 × 10^–3^ | 99.2 |
| BCC | 0.054 | 0.071 | 1.06 | 0.44 | 98.7 |
| CC | 0.072 | 0.082 | 1.07 | 0.38 | 99.0 |
| CGC | 0.098 | 0.086 | 1.10 | 0.25 | 99.1 |
| CGH | 0.056 | 0.061 | 1.06 | 0.36 | 98.2 |
| CR | 0.128 | 0.060 | 1.14 | 3.4 × 10^–2^ | 98.2 |
| CST | 0.283 | 0.065 | 1.33 | **1.2 × 10^–5^** | 98.4 |
| EC | 0.263 | 0.083 | 1.30 | **1.6 × 10^–3^** | 99.1 |
| FX | 0.098 | 0.075 | 1.10 | 0.19 | 98.8 |
| FXST | 0.241 | 0.056 | 1.27 | **2.0 × 10^–5^** | 97.9 |
| GCC | 0.106 | 0.075 | 1.11 | 0.16 | 98.8 |
| IC | 0.154 | 0.060 | 1.17 | 1.0 × 10^–2^ | 98.2 |
| IFO | 0.253 | 0.058 | 1.29 | **1.3 × 10^–5^** | 98.0 |
| PCR | –0.018 | 0.087 | 0.98 | 0.84 | 99.1 |
| PLIC | 0.092 | 0.051 | 1.10 | 7.3 × 10^–2^ | 97.4 |
| PTR | 0.052 | 0.071 | 1.05 | 0.46 | 98.7 |
| RLIC | 0.106 | 0.051 | 1.11 | 3.7 × 10^–2^ | 97.4 |
| SCC | 0.034 | 0.087 | 1.03 | 0.70 | 99.1 |
| SCR | 0.154 | 0.061 | 1.17 | 1.1 × 10^–2^ | 98.2 |
| SFO | 0.152 | 0.115 | 1.16 | 0.18 | 99.5 |
| SLF | 0.082 | 0.065 | 1.09 | 0.21 | 98.4 |
| SS | 0.153 | 0.059 | 1.17 | 1.0 × 10^–2^ | 98.1 |
| UNC | 0.178 | 0.117 | 1.19 | 0.13 | 99.5 |

**Legends of Supplementary Table 2–17**: Bold means statistical significant [*p* < 0.002 (0.05/25)].

Abbreviations: ACR, anterior corona radiata; AD, axial diffusivity; ALIC, anterior limb of internal capsule; BCC, body of corpus callosum; CC, corpus callosum; CGC, cingulum (cingulate gyrus); CGH, cingulum (hippocampus); CR, corona radiata; CST, corticospinal tract; EC, external capsule; FA, fractional anisotropy; FX, fornix; FX/ST, fornix (crus)/stria terminalis; GCC, genu of corpus callosum; IC, internal capsule; IFO, inferior fronto-occipital fasciculus; lnVR, log variability ratio; MD, mean diffusivity; PCR, posterior corona radiata; PLIC, posterior limb of internal capsule; PTR, posterior thalamic radiation; RD, radial diffusivity; RLIC, retrolenticular part of internal capsule; SCC, splenium of corpus callosum; SCR, superior corona radiata; SFO, superior fronto-occipital fasciculus; SLF, superior longitudinal fasciculus; SS, sagittal stratum; UNC, uncinate fasciculus; var, sampling variance; VR, variability ratio.

**Supplementary Table 18** Mega-analysis results of differences in fractional anisotropy (FA) between patients with schizophrenia and healthy comparison subjects.

| Region of interest | Cohen’s *d* | Standard deviation | *p* | *I^2^* (%) |
| --- | --- | --- | --- | --- |
| ACR | –0.602 | 0.095 | **2.0 × 10^–10^** | 63.5 |
| ALIC | –0.376 | 0.056 | **2.2 × 10^–11^** | 5.4 |
| Average FA | –0.444 | 0.092 | **1.6 × 10^–6^** | 61.4 |
| BCC | –0.499 | 0.094 | **1.2 × 10^–7^** | 60.8 |
| CC | –0.466 | 0.103 | **5.6 × 10^–6^** | 67.7 |
| CGC | –0.372 | 0.094 | **8.2 × 10^–5^** | 60.8 |
| CGH | –0.049 | 0.096 | 0.61 | 61.3 |
| CR | –0.355 | 0.077 | **3.9 × 10^–6^** | 44.9 |
| CST | –0.067 | 0.056 | 0.23 | 4.5 |
| EC | –0.265 | 0.080 | **9.8 × 10^–4^** | 46.3 |
| FX | –0.413 | 0.058 | **7.1 × 10^–13^** | 11.7 |
| FXST | –0.252 | 0.077 | **9.7 × 10^–4^** | 41.4 |
| GCC | –0.402 | 0.103 | **9.2 × 10^–5^** | 69.2 |
| IC | –0.075 | 0.065 | 0.25 | 23.5 |
| IFO | –0.094 | 0.057 | 0.10 | 5.7 |
| PCR | –0.203 | 0.074 | 6.0 × 10^–3^ | 38.2 |
| PLIC | 0.201 | 0.095 | 3.4 × 10^–2^ | 59.4 |
| PTR | –0.328 | 0.071 | **4.0 × 10^–6^** | 38.6 |
| RLIC | –0.063 | 0.066 | 0.35 | 26.9 |
| SCC | –0.182 | 0.097 | 6.2 × 10^–2^ | 62.9 |
| SCR | –0.001 | 0.056 | 0.98 | 5.2 |
| SFO | –0.364 | 0.053 | **7.6 × 10^–12^** | 0 |
| SLF | –0.175 | 0.057 | **2.0 × 10^–3^** | 7.9 |
| SS | –0.298 | 0.095 | **1.6 × 10^–3^** | 62.4 |
| UNC | –0.124 | 0.054 | 2.2 × 10^–2^ | 0 |

**Supplementary Table 19** Mega-analysis results of difference of mean diffusivity (MD) between patients with schizophrenia and healthy comparison subjects.

| Region of interest | Cohen’s *d* | Standard deviation | *p* | *I^2^* (%) |
| --- | --- | --- | --- | --- |
| ACR | 0.375 | 0.105 | **3.4 × 10^–4^** | 68.4 |
| ALIC | 0.209 | 0.079 | 8.5 × 10^–3^ | 47.3 |
| Average MD | 0.489 | 0.111 | **1.1 × 10^–5^** | 72.3 |
| BCC | 0.463 | 0.095 | **9.9 × 10^–7^** | 59.5 |
| CC | 0.464 | 0.116 | **6.5 × 10^–5^** | 73.5 |
| CGC | 0.278 | 0.095 | 3.4 × 10^–3^ | 61.2 |
| CGH | 0.081 | 0.105 | 0.44 | 69.7 |
| CR | 0.408 | 0.094 | **1.6 × 10^–5^** | 60.8 |
| CST | 0.118 | 0.090 | 0.19 | 56.2 |
| EC | 0.292 | 0.111 | 8.6 × 10^–3^ | 73.2 |
| FX | 0.583 | 0.102 | **1.0 × 10^–8^** | 69.3 |
| FXST | 0.296 | 0.109 | 6.5 × 10^–3^ | 70.9 |
| GCC | 0.427 | 0.136 | **1.7 × 10^–3^** | 81.0 |
| IC | 0.165 | 0.070 | 1.7 × 10^–2^ | 32.8 |
| IFO | 0.243 | 0.087 | 5.1 × 10^–3^ | 53.7 |
| PCR | 0.375 | 0.091 | **3.4 × 10^–5^** | 56.1 |
| PLIC | 0.033 | 0.059 | 0.58 | 12.5 |
| PTR | 0.223 | 0.090 | 1.3 × 10^–2^ | 56.4 |
| RLIC | 0.234 | 0.093 | 1.2 × 10^–2^ | 58.8 |
| SCC | 0.251 | 0.110 | 2.3 × 10^–2^ | 70.1 |
| SCR | 0.356 | 0.072 | **8.1 × 10^–7^** | 34.9 |
| SFO | 0.305 | 0.083 | **2.3 × 10^–4^** | 49.3 |
| SLF | 0.309 | 0.079 | **8.9 × 10^–5^** | 42.8 |
| SS | 0.230 | 0.098 | 2.0 × 10^–2^ | 62.7 |
| UNC | 0.425 | 0.090 | **2.2 × 10^–6^** | 58.5 |

**Supplementary Table 20** Mega-analysis results of differences in axial diffusivity (AD) between patients with schizophrenia and healthy comparison subjects.

| Region of interest | Cohen’s *d* | Standard deviation | *p* | *I^2^* (%) |
| --- | --- | --- | --- | --- |
| ACR | –0.047 | 0.082 | 0.57 | 49.9 |
| ALIC | –0.088 | 0.070 | 0.2 | 35.8 |
| Average AD | 0.208 | 0.105 | 4.9 × 10^–2^ | 72.1 |
| BCC | 0.005 | 0.067 | 0.94 | 26.5 |
| CC | 0.081 | 0.085 | 0.34 | 52.5 |
| CGC | –0.046 | 0.088 | 0.60 | 54.1 |
| CGH | 0.022 | 0.108 | 0.84 | 70.6 |
| CR | 0.181 | 0.074 | 1.5 × 10^–2^ | 40.0 |
| CST | 0.131 | 0.122 | 0.28 | 76.4 |
| EC | 0.017 | 0.078 | 0.83 | 46.4 |
| FX | 0.538 | 0.100 | **6.4 × 10^–8^** | 67.1 |
| FXST | –0.013 | 0.096 | 0.90 | 64.5 |
| GCC | 0.120 | 0.113 | 0.29 | 73.3 |
| IC | 0.127 | 0.064 | 4.6 × 10^–2^ | 28.9 |
| IFO | 0.072 | 0.075 | 0.34 | 37.7 |
| PCR | 0.269 | 0.081 | **9.2 × 10^–4^** | 47.7 |
| PLIC | 0.217 | 0.075 | 3.7 × 10^–3^ | 43.0 |
| PTR | –0.097 | 0.060 | 0.11 | 12.9 |
| RLIC | 0.155 | 0.082 | 5.9 × 10^–2^ | 51.9 |
| SCC | 0.121 | 0.095 | 0.20 | 61.4 |
| SCR | 0.312 | 0.069 | **5.5 × 10^–6^** | 29.7 |
| SFO | 0.021 | 0.062 | 0.73 | 19.5 |
| SLF | 0.144 | 0.052 | 6.2 × 10^–3^ | 0 |
| SS | –0.004 | 0.069 | 0.95 | 29.7 |
| UNC | 0.342 | 0.086 | **6.7 × 10^–5^** | 54.2 |

**Supplementary Table 21** Mega-analysis results of differences in radial diffusivity (RD) between patients with schizophrenia and healthy comparison subjects.

| Region of interest | Cohen’s *d* | Standard deviation | *p* | *I^2^* (%) |
| --- | --- | --- | --- | --- |
| ACR | 0.564 | 0.108 | **1.8 × 10^–7^** | 71.1 |
| ALIC | 0.359 | 0.076 | **2.1 × 10^–6^** | 40.1 |
| Average RD | 0.568 | 0.105 | **6.1 × 10^–8^** | 68.9 |
| BCC | 0.518 | 0.087 | **2.4 × 10^–9^** | 53.0 |
| CC | 0.536 | 0.102 | **1.7 × 10^–7^** | 66.7 |
| CGC | 0.383 | 0.079 | **1.2 × 10^–6^** | 44.2 |
| CGH | 0.067 | 0.096 | 0.49 | 61.9 |
| CR | 0.447 | 0.093 | **1.4 × 10^–6^** | 59.5 |
| CST | 0.121 | 0.054 | 2.4 × 10^–2^ | 0 |
| EC | 0.358 | 0.112 | **1.4 × 10^–3^** | 72.5 |
| FX | 0.580 | 0.097 | **2.3 × 10^–9^** | 66.5 |
| FXST | 0.340 | 0.078 | **1.2 × 10^–5^** | 42.1 |
| GCC | 0.491 | 0.114 | **1.6 × 10^–5^** | 73.9 |
| IC | 0.107 | 0.064 | 9.4 × 10^–2^ | 19.9 |
| IFO | 0.246 | 0.081 | 2.4 × 10^–3^ | 46.3 |
| PCR | 0.326 | 0.082 | **7.4 × 10^–5^** | 47.5 |
| PLIC | –0.135 | 0.087 | 0.12 | 52.1 |
| PTR | 0.354 | 0.089 | **7.4 × 10^–5^** | 58.2 |
| RLIC | 0.143 | 0.072 | 4.7 × 10^–2^ | 34.2 |
| SCC | 0.301 | 0.099 | 2.5 × 10^–3^ | 64.0 |
| SCR | 0.208 | 0.061 | **6.4 × 10^–4^** | 14.3 |
| SFO | 0.413 | 0.083 | **6.4 × 10^–7^** | 49.3 |
| SLF | 0.301 | 0.076 | **7.5 × 10^–5^** | 39.7 |
| SS | 0.309 | 0.100 | **1.9 × 10^–3^** | 65.0 |
| UNC | 0.320 | 0.071 | **6.3 × 10^–6^** | 34.6 |

**Supplementary Table 22** Mega-analysis results of differences in fractional anisotropy (FA) between patients with bipolar disorder and healthy comparison subjects.

| Region of interest | Cohen’s *d* | Standard deviation | *p* | *I^2^* (%) |
| --- | --- | --- | --- | --- |
| ACR | –0.225 | 0.112 | 4.3 × 10^–2^ | 35.5 |
| ALIC | –0.119 | 0.104 | 0.25 | 13.9 |
| Average FA | –0.162 | 0.126 | 0.20 | 47.9 |
| BCC | –0.351 | 0.128 | 6.0 × 10^–3^ | 46.9 |
| CC | –0.285 | 0.147 | 5.3 × 10^–2^ | 60.6 |
| CGC | –0.378 | 0.110 | **6.2 × 10^–4^** | 27.8 |
| CGH | 0.274 | 0.097 | 4.6 × 10^–3^ | 0 |
| CR | –0.102 | 0.125 | 0.41 | 44.2 |
| CST | 0.044 | 0.095 | 0.64 | 0 |
| EC | –0.103 | 0.115 | 0.37 | 28.2 |
| FX | –0.262 | 0.125 | 3.7 × 10^–2^ | 53.3 |
| FXST | –0.077 | 0.086 | 0.37 | 0 |
| GCC | –0.259 | 0.116 | 2.5 × 10^–2^ | 40.7 |
| IC | 0.007 | 0.123 | 0.95 | 35.2 |
| IFO | 0.070 | 0.118 | 0.55 | 26.8 |
| PCR | 0.025 | 0.137 | 0.86 | 48.3 |
| PLIC | 0.130 | 0.189 | 0.49 | 70.3 |
| PTR | –0.095 | 0.093 | 0.31 | 8.8 |
| RLIC | 0.153 | 0.093 | 0.10 | 0 |
| SCC | –0.085 | 0.165 | 0.61 | 64.3 |
| SCR | –0.032 | 0.106 | 0.77 | 16.1 |
| SFO | –0.204 | 0.094 | 3.0 × 10^–2^ | 0 |
| SLF | –0.077 | 0.160 | 0.63 | 62.8 |
| SS | 0.162 | 0.088 | 6.6 × 10^–2^ | 0 |
| UNC | –0.158 | 0.131 | 0.23 | 41.2 |

**Supplementary Table 23** Mega-analysis results of differences in mean diffusivity (MD) between patients with bipolar disorder and healthy comparison subjects.

| Region of interest | Cohen’s *d* | Standard deviation | *p* | *I^2^* (%) |
| --- | --- | --- | --- | --- |
| ACR | 0.176 | 0.111 | 0.11 | 31.0 |
| ALIC | 0.156 | 0.086 | 7.1 × 10^–2^ | 0 |
| Average MD | 0.259 | 0.086 | 2.5 × 10^–3^ | 0 |
| BCC | 0.287 | 0.088 | **1.2 × 10^–3^** | 0 |
| CC | 0.254 | 0.093 | 6.4 × 10^–3^ | 8.1 |
| CGC | 0.104 | 0.092 | 0.26 | 0 |
| CGH | –0.029 | 0.095 | 0.76 | 0 |
| CR | 0.194 | 0.088 | 2.7 × 10^–2^ | 0 |
| CST | –0.034 | 0.133 | 0.80 | 46.5 |
| EC | 0.119 | 0.085 | 0.16 | 0 |
| FX | 0.406 | 0.122 | **8.3 × 10^–4^** | 53.0 |
| FXST | 0.113 | 0.090 | 0.21 | 0 |
| GCC | 0.295 | 0.133 | 2.6 × 10^–2^ | 51.6 |
| IC | 0.171 | 0.091 | 6.1 × 10^–2^ | 0 |
| IFO | 0.073 | 0.112 | 0.51 | 23.1 |
| PCR | 0.131 | 0.092 | 0.15 | 0 |
| PLIC | 0.154 | 0.121 | 0.21 | 32.1 |
| PTR | 0.008 | 0.094 | 0.93 | 0 |
| RLIC | 0.063 | 0.091 | 0.49 | 0 |
| SCC | 0.087 | 0.101 | 0.39 | 12.6 |
| SCR | 0.208 | 0.087 | 1.7 × 10^–2^ | 0 |
| SFO | 0.227 | 0.085 | 7.6 × 10^–3^ | 0 |
| SLF | 0.158 | 0.095 | 9.5 × 10^–2^ | 0 |
| SS | 0.141 | 0.090 | 0.12 | 0 |
| UNC | 0.206 | 0.088 | 2.0 × 10^–2^ | 0 |

**Supplementary Table 24** Mega-analysis results of differences in axial diffusivity (AD) between patients with bipolar disorder and healthy comparison subjects.

| Region of interest | Cohen’s *d* | Standard deviation | *p* | *I^2^* (%) |
| --- | --- | --- | --- | --- |
| ACR | 0.017 | 0.125 | 0.89 | 37.4 |
| ALIC | 0.110 | 0.088 | 0.21 | 0 |
| Average AD | 0.181 | 0.090 | 4.5 × 10^–2^ | 0 |
| BCC | –0.023 | 0.097 | 0.81 | 0 |
| CC | 0.041 | 0.094 | 0.66 | 0 |
| CGC | –0.101 | 0.143 | 0.48 | 50.0 |
| CGH | 0.264 | 0.159 | 9.8 × 10^–2^ | 60.5 |
| CR | 0.162 | 0.128 | 0.21 | 45.5 |
| CST | –0.007 | 0.121 | 0.95 | 29.9 |
| EC | 0.091 | 0.087 | 0.30 | 0 |
| FX | 0.430 | 0.124 | **5.3 × 10^–4^** | 51.5 |
| FXST | 0.051 | 0.099 | 0.61 | 7.6 |
| GCC | 0.142 | 0.113 | 0.21 | 26.0 |
| IC | 0.255 | 0.091 | 5.3 × 10^–3^ | 0 |
| IFO | 0.078 | 0.097 | 0.43 | 0 |
| PCR | 0.202 | 0.124 | 0.10 | 40.1 |
| PLIC | 0.314 | 0.097 | **1.2 × 10^–3^** | 0 |
| PTR | –0.138 | 0.099 | 0.16 | 0 |
| RLIC | 0.181 | 0.094 | 5.4 × 10^–2^ | 0 |
| SCC | 0.024 | 0.095 | 0.80 | 0 |
| SCR | 0.209 | 0.102 | 4.0 × 10^–2^ | 18.2 |
| SFO | 0.132 | 0.086 | 0.13 | 0 |
| SLF | 0.099 | 0.120 | 0.41 | 32.9 |
| SS | 0.242 | 0.096 | 1.1 × 10^–2^ | 0 |
| UNC | 0.086 | 0.088 | 0.33 | 0.3 |

**Supplementary Table 25** Mega-analysis results of differences in radial diffusivity (RD) between patients with bipolar disorder and healthy comparison subjects.

| Region of interest | Cohen’s *d* | Standard deviation | *p* | *I^2^* (%) |
| --- | --- | --- | --- | --- |
| ACR | 0.227 | 0.101 | 2.4 × 10^–2^ | 21.3 |
| ALIC | 0.185 | 0.103 | 7.4 × 10^–2^ | 23.1 |
| Average RD | 0.270 | 0.083 | **1.2 × 10^–3^** | 0 |
| BCC | 0.346 | 0.098 | **4.3 × 10^–4^** | 16.9 |
| CC | 0.295 | 0.114 | 9.9 × 10^–3^ | 37.2 |
| CGC | 0.250 | 0.090 | 5.3 × 10^–3^ | 0 |
| CGH | –0.213 | 0.096 | 2.6 × 10^–2^ | 0 |
| CR | 0.200 | 0.088 | 2.4 × 10^–2^ | 0 |
| CST | –0.011 | 0.120 | 0.93 | 36.0 |
| EC | 0.134 | 0.087 | 0.12 | 0 |
| FX | 0.384 | 0.114 | **7.8 × 10^–4^** | 48.1 |
| FXST | 0.151 | 0.083 | 6.9 × 10^–2^ | 0 |
| GCC | 0.296 | 0.115 | 1.0 × 10^–2^ | 41.2 |
| IC | 0.079 | 0.105 | 0.45 | 19.4 |
| IFO | 0.029 | 0.119 | 0.81 | 28.4 |
| PCR | 0.077 | 0.093 | 0.41 | 0 |
| PLIC | –0.057 | 0.178 | 0.75 | 67.0 |
| PTR | 0.092 | 0.090 | 0.30 | 0 |
| RLIC | –0.009 | 0.091 | 0.92 | 0 |
| SCC | 0.123 | 0.130 | 0.34 | 44.9 |
| SCR | 0.166 | 0.091 | 6.9 × 10^–2^ | 0 |
| SFO | 0.258 | 0.087 | 3.1 × 10^–3^ | 0 |
| SLF | 0.173 | 0.094 | 6.6 × 10^–2^ | 0 |
| SS | 0.064 | 0.088 | 0.47 | 0 |
| UNC | 0.247 | 0.092 | 7.0 × 10^–3^ | 0 |

**Supplementary Table 26** Mega-analysis results of differences in fractional anisotropy (FA) between individuals with autism spectrum disorder and healthy comparison subjects.

| Region of interest | Cohen’s *d* | Standard deviation | *p* | *I^2^* (%) |
| --- | --- | --- | --- | --- |
| ACR | –0.189 | 0.154 | 0.22 | 50.8 |
| ALIC | –0.172 | 0.179 | 0.34 | 58.9 |
| Average FA | –0.255 | 0.122 | 3.6 × 10^–2^ | 23.3 |
| BCC | –0.342 | 0.110 | **1.9 × 10^–3^** | 0 |
| CC | –0.278 | 0.107 | 9.7 × 10^–3^ | 0 |
| CGC | –0.274 | 0.109 | 1.2 × 10^–2^ | 0 |
| CGH | –0.150 | 0.152 | 0.32 | 42.0 |
| CR | –0.192 | 0.106 | 6.9 × 10^–2^ | 1.1 |
| CST | –0.170 | 0.111 | 0.13 | 0 |
| EC | –0.155 | 0.109 | 0.15 | 0 |
| FX | –0.136 | 0.106 | 0.20 | 0 |
| FXST | –0.238 | 0.107 | 2.6 × 10^–2^ | 0 |
| GCC | –0.173 | 0.103 | 9.3 × 10^–2^ | 0 |
| IC | –0.188 | 0.133 | 0.16 | 29.5 |
| IFO | –0.239 | 0.112 | 3.3 × 10^–2^ | 0 |
| PCR | –0.200 | 0.107 | 6.1 × 10^–2^ | 0 |
| PLIC | –0.195 | 0.153 | 0.20 | 43.4 |
| PTR | –0.170 | 0.156 | 0.27 | 51.4 |
| RLIC | –0.117 | 0.108 | 0.28 | 0 |
| SCC | –0.128 | 0.109 | 0.24 | 0 |
| SCR | –0.111 | 0.115 | 0.34 | 7.1 |
| SFO | –0.161 | 0.132 | 0.22 | 27.0 |
| SLF | –0.213 | 0.131 | 0.10 | 29.6 |
| SS | –0.079 | 0.137 | 0.56 | 33.2 |
| UNC | –0.380 | 0.219 | 8.3 × 10^–2^ | 70.9 |

**Supplementary Table 27** Mega-analysis results of differences in mean diffusivity (MD) between individuals with autism spectrum disorder and healthy comparison subjects.

| Region of interest | Cohen’s *d* | Standard deviation | *p* | *I^2^* (%) |
| --- | --- | --- | --- | --- |
| ACR | 0.025 | 0.152 | 0.87 | 42.7 |
| ALIC | 0.116 | 0.171 | 0.50 | 57.7 |
| Average MD | 0.218 | 0.121 | 7.1 × 10^–2^ | 13.7 |
| BCC | 0.221 | 0.112 | 4.8 × 10^–2^ | 0 |
| CC | 0.222 | 0.112 | 4.7 × 10^–2^ | 0 |
| CGC | 0.170 | 0.111 | 0.13 | 0 |
| CGH | 0.039 | 0.132 | 0.77 | 30.8 |
| CR | 0.064 | 0.157 | 0.68 | 45.4 |
| CST | 0.035 | 0.173 | 0.84 | 58.7 |
| EC | 0.147 | 0.108 | 0.17 | 0 |
| FX | 0.100 | 0.107 | 0.35 | 0 |
| FXST | 0.291 | 0.161 | 7.1 × 10^–2^ | 52.6 |
| GCC | 0.091 | 0.111 | 0.41 | 0 |
| IC | 0.156 | 0.142 | 0.27 | 40.6 |
| IFO | 0.024 | 0.109 | 0.83 | 0 |
| PCR | 0.104 | 0.145 | 0.48 | 35.6 |
| PLIC | 0.243 | 0.141 | 8.5 × 10^–2^ | 41.8 |
| PTR | 0.100 | 0.111 | 0.37 | 0 |
| RLIC | 0.040 | 0.110 | 0.72 | 0 |
| SCC | 0.263 | 0.113 | 1.9 × 10^–2^ | 0 |
| SCR | 0.079 | 0.165 | 0.63 | 50.0 |
| SFO | 0.108 | 0.161 | 0.50 | 50.0 |
| SLF | 0.174 | 0.112 | 0.12 | 0 |
| SS | 0.085 | 0.132 | 0.52 | 24.0 |
| UNC | 0.175 | 0.128 | 0.17 | 23.4 |

**Supplementary Table 28** Mega-analysis results of differences in axial diffusivity (AD) between individuals with autism spectrum disorder and healthy comparison subjects.

| Region of interest | Cohen’s *d* | Standard deviation | *p* | *I^2^* (%) |
| --- | --- | --- | --- | --- |
| ACR | –0.142 | 0.106 | 0.18 | 0 |
| ALIC | 0.040 | 0.102 | 0.70 | 0 |
| Average AD | 0.045 | 0.101 | 0.66 | 0 |
| BCC | –0.152 | 0.108 | 0.16 | 0 |
| CC | –0.043 | 0.105 | 0.68 | 0 |
| CGC | –0.146 | 0.109 | 0.18 | 0 |
| CGH | –0.111 | 0.107 | 0.30 | 0 |
| CR | –0.090 | 0.136 | 0.51 | 34.4 |
| CST | –0.063 | 0.200 | 0.75 | 67.9 |
| EC | –0.035 | 0.125 | 0.78 | 25.2 |
| FX | 0.065 | 0.108 | 0.55 | 0 |
| FXST | 0.040 | 0.099 | 0.68 | 0 |
| GCC | –0.072 | 0.103 | 0.48 | 0 |
| IC | 0.042 | 0.098 | 0.67 | 0 |
| IFO | –0.279 | 0.199 | 0.16 | 66.6 |
| PCR | –0.061 | 0.147 | 0.68 | 39.9 |
| PLIC | 0.096 | 0.101 | 0.34 | 0 |
| PTR | –0.103 | 0.185 | 0.58 | 61.0 |
| RLIC | –0.045 | 0.105 | 0.67 | 0 |
| SCC | 0.154 | 0.112 | 0.17 | 5.0 |
| SCR | –0.061 | 0.197 | 0.76 | 65.9 |
| SFO | –0.011 | 0.106 | 0.92 | 1.6 |
| SLF | –0.005 | 0.159 | 0.97 | 50.9 |
| SS | –0.014 | 0.108 | 0.89 | 0 |
| UNC | –0.096 | 0.110 | 0.38 | 0 |

**Supplementary Table 29** Mega-analysis results of differences in radial diffusivity (RD) between individuals with autism spectrum disorder and healthy comparison subjects.

| Region of interest | Cohen’s *d* | Standard deviation | *p* | *I^2^* (%) |
| --- | --- | --- | --- | --- |
| ACR | 0.113 | 0.158 | 0.48 | 48.3 |
| ALIC | 0.162 | 0.204 | 0.43 | 68.1 |
| Average RD | 0.275 | 0.122 | 2.4 × 10^–2^ | 15.8 |
| BCC | 0.318 | 0.111 | 4.1 × 10^–3^ | 0 |
| CC | 0.297 | 0.110 | 6.8 × 10^–3^ | 0 |
| CGC | 0.309 | 0.111 | 5.3 × 10^–3^ | 0 |
| CGH | 0.159 | 0.176 | 0.37 | 57.9 |
| CR | 0.146 | 0.132 | 0.27 | 26.1 |
| CST | 0.096 | 0.154 | 0.54 | 46.9 |
| EC | 0.185 | 0.110 | 9.2 × 10^–2^ | 0 |
| FX | 0.116 | 0.106 | 0.28 | 0 |
| FXST | 0.351 | 0.125 | 4.9 × 10^–3^ | 17.8 |
| GCC | 0.176 | 0.107 | 0.10 | 0 |
| IC | 0.191 | 0.151 | 0.21 | 42.3 |
| IFO | 0.175 | 0.111 | 0.11 | 0 |
| PCR | 0.175 | 0.111 | 0.11 | 0 |
| PLIC | 0.246 | 0.173 | 0.15 | 55.4 |
| PTR | 0.209 | 0.106 | 5.0 × 10^–2^ | 0 |
| RLIC | 0.091 | 0.111 | 0.41 | 0 |
| SCC | 0.240 | 0.110 | 2.9 × 10^–2^ | 0 |
| SCR | 0.130 | 0.111 | 0.24 | 0 |
| SFO | 0.175 | 0.151 | 0.25 | 41.0 |
| SLF | 0.184 | 0.111 | 0.10 | 0 |
| SS | 0.128 | 0.147 | 0.38 | 38.2 |
| UNC | 0.357 | 0.197 | 7.0 × 10^–2^ | 65.0 |

**Supplementary Table 30** Mega-analysis results of differences in fractional anisotropy (FA) between patients with major depressive disorder and healthy comparison subjects.

| Region of interest | Cohen’s *d* | Standard deviation | *p* | *I^2^* (%) |
| --- | --- | --- | --- | --- |
| ACR | –0.189 | 0.072 | 8.5 × 10^–3^ | 0 |
| ALIC | 0.056 | 0.079 | 0.48 | 0 |
| Average FA | –0.092 | 0.072 | 0.20 | 0 |
| BCC | –0.104 | 0.076 | 0.17 | 0 |
| CC | –0.108 | 0.075 | 0.15 | 0 |
| CGC | –0.030 | 0.077 | 0.70 | 0 |
| CGH | 0.226 | 0.083 | 6.4 × 10^–3^ | 0 |
| CR | –0.099 | 0.074 | 0.18 | 0 |
| CST | 0.149 | 0.082 | 7.0 × 10^–2^ | 0 |
| EC | –0.042 | 0.101 | 0.68 | 34.0 |
| FX | –0.118 | 0.070 | 9.2 × 10^–2^ | 0 |
| FXST | 0.017 | 0.073 | 0.81 | 0 |
| GCC | –0.193 | 0.072 | 7.1 × 10^–3^ | 0 |
| IC | 0.143 | 0.081 | 7.7 × 10^–2^ | 0 |
| IFO | 0.072 | 0.083 | 0.39 | 0 |
| PCR | –0.040 | 0.079 | 0.61 | 0 |
| PLIC | 0.248 | 0.118 | 3.6 × 10^–2^ | 44.7 |
| PTR | –0.078 | 0.075 | 0.30 | 0 |
| RLIC | 0.061 | 0.078 | 0.43 | 0 |
| SCC | 0.037 | 0.123 | 0.76 | 51.0 |
| SCR | 0.015 | 0.080 | 0.85 | 0 |
| SFO | 0.074 | 0.079 | 0.35 | 0 |
| SLF | –0.006 | 0.080 | 0.94 | 0 |
| SS | 0.023 | 0.075 | 0.75 | 0 |
| UNC | 0.004 | 0.081 | 0.96 | 0 |

**Supplementary Table 31** Mega-analysis results of differences in mean diffusivity (MD) between patients with major depressive disorder and healthy comparison subjects.

| Region of interest | Cohen’s *d* | Standard deviation | *p* | *I^2^* (%) |
| --- | --- | --- | --- | --- |
| ACR | 0.053 | 0.093 | 0.57 | 29.2 |
| ALIC | –0.047 | 0.068 | 0.49 | 0 |
| Average MD | 0.141 | 0.069 | 4.1 × 10^–2^ | 0 |
| BCC | 0.067 | 0.073 | 0.36 | 0 |
| CC | 0.075 | 0.097 | 0.44 | 36.2 |
| CGC | 0.032 | 0.075 | 0.67 | 0 |
| CGH | –0.097 | 0.094 | 0.30 | 23.3 |
| CR | 0.041 | 0.073 | 0.57 | 1.1 |
| CST | –0.140 | 0.143 | 0.33 | 64.2 |
| EC | 0.131 | 0.068 | 5.2 × 10^–2^ | 0 |
| FX | 0.179 | 0.067 | 7.2 × 10^–3^ | 0 |
| FXST | 0.069 | 0.074 | 0.35 | 0 |
| GCC | 0.124 | 0.107 | 0.24 | 46.5 |
| IC | –0.077 | 0.074 | 0.30 | 0 |
| IFO | –0.034 | 0.099 | 0.73 | 27.5 |
| PCR | 0.024 | 0.076 | 0.75 | 0 |
| PLIC | –0.133 | 0.112 | 0.24 | 42.1 |
| PTR | –0.004 | 0.082 | 0.96 | 7.1 |
| RLIC | 0.007 | 0.074 | 0.93 | 0 |
| SCC | 0.004 | 0.127 | 0.98 | 57.7 |
| SCR | 0.008 | 0.072 | 0.91 | 0 |
| SFO | –0.043 | 0.069 | 0.53 | 0 |
| SLF | –0.006 | 0.085 | 0.94 | 10.3 |
| SS | 0.004 | 0.100 | 0.97 | 37.4 |
| UNC | 0.065 | 0.073 | 0.37 | 0 |

**Supplementary Table 32** Mega-analysis results of differences in axial diffusivity (AD) between patients with major depressive disorder and healthy comparison subjects.

| Region of interest | Cohen’s *d* | Standard deviation | *p* | *I^2^* (%) |
| --- | --- | --- | --- | --- |
| ACR | –0.061 | 0.109 | 0.57 | 41.8 |
| ALIC | –0.041 | 0.093 | 0.66 | 35.5 |
| Average AD | 0.101 | 0.073 | 0.17 | 4.1 |
| BCC | –0.045 | 0.079 | 0.57 | 0 |
| CC | –0.005 | 0.093 | 0.96 | 27.1 |
| CGC | 0.064 | 0.114 | 0.57 | 44.3 |
| CGH | 0.070 | 0.113 | 0.53 | 42.9 |
| CR | –0.002 | 0.089 | 0.99 | 22.4 |
| CST | –0.118 | 0.167 | 0.48 | 72.6 |
| EC | 0.128 | 0.074 | 8.6 × 10^–2^ | 9.2 |
| FX | 0.204 | 0.068 | 2.9 × 10^–3^ | 0 |
| FXST | 0.045 | 0.078 | 0.57 | 0 |
| GCC | –0.028 | 0.136 | 0.84 | 64.3 |
| IC | 0.008 | 0.092 | 0.93 | 31.7 |
| IFO | –0.023 | 0.081 | 0.78 | 0 |
| PCR | 0.002 | 0.077 | 0.98 | 0 |
| PLIC | 0.023 | 0.109 | 0.83 | 40.9 |
| PTR | –0.047 | 0.103 | 0.65 | 30.1 |
| RLIC | 0.053 | 0.076 | 0.49 | 0 |
| SCC | 0.046 | 0.078 | 0.55 | 0 |
| SCR | 0.032 | 0.086 | 0.71 | 17.0 |
| SFO | 0.021 | 0.083 | 0.80 | 19.5 |
| SLF | –0.002 | 0.117 | 0.99 | 48.5 |
| SS | 0.040 | 0.123 | 0.75 | 53.9 |
| UNC | 0.066 | 0.075 | 0.37 | 0 |

**Supplementary Table 33** Mega-analysis results of differences in radial diffusivity (RD) between patients with major depressive disorder and healthy comparison subjects.

| Region of interest | Cohen’s *d* | Standard deviation | *p* | *I^2^* (%) |
| --- | --- | --- | --- | --- |
| ACR | 0.116 | 0.071 | 0.10 | 0 |
| ALIC | –0.028 | 0.071 | 0.70 | 0 |
| Average RD | 0.151 | 0.068 | 2.6 × 10^–2^ | 0 |
| BCC | 0.101 | 0.074 | 0.17 | 0 |
| CC | 0.108 | 0.092 | 0.24 | 28.9 |
| CGC | 0.021 | 0.073 | 0.78 | 0 |
| CGH | –0.189 | 0.092 | 4.0 × 10^–2^ | 17.6 |
| CR | 0.064 | 0.073 | 0.38 | 0 |
| CST | –0.093 | 0.116 | 0.42 | 46.2 |
| EC | 0.113 | 0.075 | 0.13 | 10.5 |
| FX | 0.165 | 0.067 | 1.3 × 10^–2^ | 0 |
| FXST | 0.063 | 0.071 | 0.37 | 0 |
| GCC | 0.183 | 0.070 | 9.3 × 10^–3^ | 0 |
| IC | –0.097 | 0.076 | 0.20 | 0 |
| IFO | –0.015 | 0.082 | 0.86 | 0 |
| PCR | 0.034 | 0.078 | 0.66 | 0 |
| PLIC | –0.197 | 0.108 | 6.7 × 10^–2^ | 35.1 |
| PTR | 0.030 | 0.076 | 0.69 | 0 |
| RLIC | –0.014 | 0.075 | 0.85 | 0 |
| SCC | 0.003 | 0.129 | 0.98 | 58.1 |
| SCR | <0.001 | 0.075 | 1.00 | 0 |
| SFO | –0.073 | 0.072 | 0.30 | 0 |
| SLF | –0.001 | 0.078 | 0.99 | 0 |
| SS | 0.007 | 0.074 | 0.93 | 2.9 |
| UNC | 0.056 | 0.075 | 0.46 | 0 |

**Legends of Supplementary Table 18–33**: Bold means statistical significant [*p* < 0.002 (0.05/25)].

Abbreviations: ACR, anterior corona radiata; AD, axial diffusivity; ALIC, anterior limb of internal capsule; BCC, body of corpus callosum; CC, corpus callosum; CGC, cingulum (cingulate gyrus); CGH, cingulum (hippocampus); CR, corona radiata; CST, corticospinal tract; EC, external capsule; FA, fractional anisotropy; FX, fornix; FX/ST, fornix (crus)/stria terminalis; GCC, genu of corpus callosum; IC, internal capsule; IFO, inferior fronto-occipital fasciculus; MD, mean diffusivity; PCR, posterior corona radiata; PLIC, posterior limb of internal capsule; PTR, posterior thalamic radiation; RD, radial diffusivity; RLIC, retrolenticular part of internal capsule; SCC, splenium of corpus callosum; SCR, superior corona radiata; SFO, superior fronto-occipital fasciculus; SLF, superior longitudinal fasciculus; SS, sagittal stratum; UNC, uncinate fasciculus.

**Supplementary Table 34** Mega-analysis results of differences in fractional anisotropy (FA) between patients with schizophrenia and patients with bipolar disorder.

| Region of interest | Cohen’s *d* | Standard deviation | *p* | *I^2^* (%) |
| --- | --- | --- | --- | --- |
| ACR | –0.364 | 0.132 | 5.9 × 10^–3^ | 37.2 |
| ALIC | –0.211 | 0.103 | 4.1 × 10^–2^ | 0 |
| Average FA | –0.264 | 0.125 | 3.5 × 10^–2^ | 26.8 |
| BCC | –0.209 | 0.099 | 3.5 × 10^–2^ | 0 |
| CC | –0.197 | 0.100 | 4.9 × 10^–2^ | 0 |
| CGC | –0.039 | 0.102 | 0.70 | 0 |
| CGH | 0.031 | 0.186 | 0.87 | 61.7 |
| CR | –0.291 | 0.119 | 1.4 × 10^–2^ | 21.8 |
| CST | –0.091 | 0.162 | 0.57 | 49.1 |
| EC | –0.128 | 0.151 | 0.40 | 42.4 |
| FX | –0.209 | 0.160 | 0.19 | 53.9 |
| FXST | 0.008 | 0.106 | 0.94 | 0 |
| GCC | –0.161 | 0.100 | 0.11 | 0 |
| IC | –0.128 | 0.155 | 0.41 | 46.1 |
| IFO | –0.205 | 0.109 | 6.0 × 10^–2^ | 0 |
| PCR | –0.280 | 0.136 | 3.9 × 10^–2^ | 33.1 |
| PLIC | 0.052 | 0.129 | 0.69 | 23.2 |
| PTR | –0.158 | 0.132 | 0.23 | 34.0 |
| RLIC | –0.203 | 0.184 | 0.27 | 60.6 |
| SCC | –0.090 | 0.107 | 0.40 | 0 |
| SCR | –0.091 | 0.102 | 0.38 | 0 |
| SFO | –0.135 | 0.101 | 0.18 | 0 |
| SLF | –0.117 | 0.138 | 0.40 | 32.6 |
| SS | –0.402 | 0.178 | 2.4 × 10^–2^ | 59.2 |
| UNC | –0.149 | 0.106 | 0.16 | 0 |

**Supplementary Table 35** Mega-analysis results of difference of mean diffusivity (MD) between patients with schizophrenia and patients with bipolar disorder.

| Region of interest | Cohen’s *d* | Standard deviation | *p* | *I^2^* (%) |
| --- | --- | --- | --- | --- |
| ACR | 0.322 | 0.176 | 6.7 × 10^–2^ | 63.4 |
| ALIC | 0.032 | 0.103 | 0.75 | 5.2 |
| Average MD | 0.216 | 0.159 | 0.17 | 53.3 |
| BCC | 0.165 | 0.099 | 9.5 × 10^–2^ | 0 |
| CC | 0.145 | 0.099 | 0.14 | 0 |
| CGC | 0.188 | 0.173 | 0.28 | 55.3 |
| CGH | 0.069 | 0.106 | 0.51 | 0 |
| CR | 0.252 | 0.156 | 0.11 | 53.4 |
| CST | –0.076 | 0.105 | 0.47 | 0 |
| EC | 0.214 | 0.172 | 0.21 | 59.0 |
| FX | 0.107 | 0.113 | 0.34 | 22.9 |
| FXST | 0.018 | 0.195 | 0.92 | 64.3 |
| GCC | 0.114 | 0.150 | 0.45 | 48.9 |
| IC | 0.069 | 0.152 | 0.65 | 45.7 |
| IFO | 0.234 | 0.107 | 2.8 × 10^–2^ | 0 |
| PCR | 0.159 | 0.123 | 0.20 | 26.4 |
| PLIC | 0.002 | 0.151 | 0.99 | 41.2 |
| PTR | 0.126 | 0.105 | 0.23 | 4.1 |
| RLIC | 0.096 | 0.145 | 0.50 | 37.0 |
| SCC | 0.143 | 0.106 | 0.18 | 0 |
| SCR | 0.195 | 0.142 | 0.17 | 42.5 |
| SFO | 0.141 | 0.154 | 0.36 | 48.6 |
| SLF | 0.207 | 0.149 | 0.17 | 43.5 |
| SS | 0.122 | 0.166 | 0.46 | 52.1 |
| UNC | 0.257 | 0.098 | 8.8 × 10^–3^ | 0 |

**Supplementary Table 36** Mega-analysis results of differences in axial diffusivity (AD) between patients with schizophrenia and patients with bipolar disorder.

| Region of interest | Cohen’s *d* | Standard deviation | *p* | *I^2^* (%) |
| --- | --- | --- | --- | --- |
| ACR | 0.056 | 0.119 | 0.64 | 19.8 |
| ALIC | –0.108 | 0.101 | 0.29 | 0 |
| Average AD | 0.121 | 0.156 | 0.44 | 48.7 |
| BCC | 0.018 | 0.106 | 0.86 | 0 |
| CC | 0.012 | 0.105 | 0.91 | 0 |
| CGC | 0.068 | 0.108 | 0.53 | 0 |
| CGH | –0.084 | 0.184 | 0.65 | 59.0 |
| CR | 0.089 | 0.110 | 0.42 | 11.8 |
| CST | –0.078 | 0.107 | 0.47 | 0 |
| EC | 0.134 | 0.145 | 0.35 | 42.5 |
| FX | 0.059 | 0.094 | 0.53 | 0 |
| FXST | –0.101 | 0.149 | 0.50 | 41.4 |
| GCC | –0.012 | 0.155 | 0.94 | 45.1 |
| IC | –0.034 | 0.102 | 0.74 | 0 |
| IFO | 0.078 | 0.106 | 0.46 | 0 |
| PCR | 0.054 | 0.142 | 0.70 | 42.5 |
| PLIC | –0.015 | 0.105 | 0.88 | 0 |
| PTR | 0.077 | 0.107 | 0.47 | 0 |
| RLIC | 0.017 | 0.107 | 0.87 | 3.6 |
| SCC | 0.050 | 0.105 | 0.63 | 0 |
| SCR | 0.104 | 0.102 | 0.31 | 0 |
| SFO | 0.047 | 0.122 | 0.70 | 23.5 |
| SLF | 0.083 | 0.104 | 0.42 | 0 |
| SS | –0.038 | 0.165 | 0.82 | 48.7 |
| UNC | 0.339 | 0.160 | 3.5 × 10^–2^ | 56.7 |

**Supplementary Table 37** Mega-analysis results of differences in radial diffusivity (RD) between patients with schizophrenia and patients with bipolar disorder.

| Region of interest | Cohen’s *d* | Standard deviation | *p* | *I^2^* (%) |
| --- | --- | --- | --- | --- |
| ACR | 0.393 | 0.176 | 2.6 × 10^–2^ | 64.3 |
| ALIC | 0.099 | 0.110 | 0.37 | 12.3 |
| Average RD | 0.248 | 0.151 | 0.10 | 50.3 |
| BCC | 0.212 | 0.098 | 3.0 × 10^–2^ | 0 |
| CC | 0.205 | 0.097 | 3.6 × 10^–2^ | 0 |
| CGC | 0.159 | 0.161 | 0.33 | 51.4 |
| CGH | 0.090 | 0.156 | 0.56 | 44.8 |
| CR | 0.272 | 0.155 | 7.9 × 10^–2^ | 52.9 |
| CST | –0.045 | 0.103 | 0.66 | 0 |
| EC | 0.179 | 0.170 | 0.29 | 57.6 |
| FX | 0.131 | 0.121 | 0.28 | 31.9 |
| FXST | 0.011 | 0.191 | 0.95 | 62.4 |
| GCC | 0.150 | 0.108 | 0.16 | 13.8 |
| IC | 0.106 | 0.175 | 0.54 | 57.4 |
| IFO | 0.264 | 0.107 | 1.4 × 10^–2^ | 0 |
| PCR | 0.198 | 0.100 | 4.8 × 10^–2^ | 0 |
| PLIC | –0.021 | 0.159 | 0.90 | 46.5 |
| PTR | 0.151 | 0.109 | 0.16 | 11.3 |
| RLIC | 0.145 | 0.177 | 0.41 | 57.0 |
| SCC | 0.148 | 0.105 | 0.16 | 0 |
| SCR | 0.116 | 0.113 | 0.31 | 16.4 |
| SFO | 0.113 | 0.145 | 0.43 | 41.8 |
| SLF | 0.204 | 0.152 | 0.18 | 45.8 |
| SS | 0.207 | 0.181 | 0.25 | 60.5 |
| UNC | 0.194 | 0.101 | 5.6 × 10^–2^ | 0 |

**Supplementary Table 38** Mega-analysis results of differences in fractional anisotropy (FA) between patients with schizophrenia and individuals with autism spectrum disorder.

| Region of interest | Cohen’s *d* | Standard deviation | *p* | *I^2^* (%) |
| --- | --- | --- | --- | --- |
| ACR | –0.353 | 0.174 | 4.2 × 10^–2^ | 17.8 |
| ALIC | –0.155 | 0.309 | 0.61 | 69.6 |
| Average FA | –0.122 | 0.218 | 0.58 | 46.3 |
| BCC | –0.098 | 0.164 | 0.55 | 0 |
| CC | –0.086 | 0.185 | 0.64 | 23.1 |
| CGC | 0.000 | 0.165 | 1.00 | 0 |
| CGH | 0.189 | 0.249 | 0.45 | 51.7 |
| CR | 0.052 | 0.157 | 0.74 | 0 |
| CST | –0.017 | 0.164 | 0.92 | 0 |
| EC | 0.135 | 0.166 | 0.42 | 0 |
| FX | –0.601 | 0.163 | **2.2 × 10^–4^** | 0 |
| FXST | –0.191 | 0.167 | 0.25 | 0 |
| GCC | –0.152 | 0.207 | 0.46 | 40.9 |
| IC | 0.221 | 0.231 | 0.34 | 44.9 |
| IFO | 0.303 | 0.173 | 8.0 × 10^–2^ | 0 |
| PCR | 0.287 | 0.161 | 7.4 × 10^–2^ | 0 |
| PLIC | 0.529 | 0.229 | 2.1 × 10^–2^ | 40.1 |
| PTR | –0.116 | 0.319 | 0.72 | 71.7 |
| RLIC | 0.156 | 0.166 | 0.35 | 0 |
| SCC | 0.058 | 0.167 | 0.73 | 1.1 |
| SCR | 0.394 | 0.166 | 1.8 × 10^–2^ | 0 |
| SFO | –0.144 | 0.236 | 0.54 | 45.4 |
| SLF | 0.136 | 0.206 | 0.51 | 30.9 |
| SS | –0.270 | 0.292 | 0.36 | 65.9 |
| UNC | 0.427 | 0.294 | 0.15 | 66.5 |

**Supplementary Table 39** Mega-analysis results of differences in mean diffusivity (MD) between patients with schizophrenia and individuals with autism spectrum disorder.

| Region of interest | Cohen’s *d* | Standard deviation | *p* | *I^2^* (%) |
| --- | --- | --- | --- | --- |
| ACR | 0.168 | 0.187 | 0.37 | 26.6 |
| ALIC | 0.056 | 0.157 | 0.72 | 0 |
| Average MD | 0.199 | 0.168 | 0.23 | 3.0 |
| BCC | 0.146 | 0.165 | 0.37 | 0 |
| CC | 0.023 | 0.184 | 0.90 | 20.3 |
| CGC | –0.147 | 0.173 | 0.39 | 0 |
| CGH | –0.298 | 0.243 | 0.22 | 48.2 |
| CR | 0.218 | 0.198 | 0.27 | 33.6 |
| CST | –0.052 | 0.210 | 0.81 | 31.1 |
| EC | –0.039 | 0.170 | 0.82 | 0 |
| FX | 0.667 | 0.160 | **2.9 × 10^–5^** | 0 |
| FXST | 0.010 | 0.169 | 0.96 | 0 |
| GCC | 0.023 | 0.300 | 0.94 | 70.2 |
| IC | 0.028 | 0.165 | 0.86 | 0 |
| IFO | 0.247 | 0.165 | 0.14 | 0 |
| PCR | 0.224 | 0.163 | 0.17 | 2.7 |
| PLIC | –0.222 | 0.193 | 0.25 | 22.7 |
| PTR | –0.028 | 0.163 | 0.86 | 0 |
| RLIC | 0.254 | 0.172 | 0.14 | 0 |
| SCC | –0.187 | 0.168 | 0.27 | 0 |
| SCR | 0.260 | 0.213 | 0.22 | 40.8 |
| SFO | 0.111 | 0.160 | 0.49 | 0 |
| SLF | 0.125 | 0.170 | 0.46 | 0 |
| SS | 0.068 | 0.167 | 0.68 | 0 |
| UNC | 0.067 | 0.166 | 0.68 | 18.6 |

**Supplementary Table 40** Mega-analysis results of differences in axial diffusivity (AD) between patients with schizophrenia and individuals with autism spectrum disorder.

| Region of interest | Cohen’s *d* | Standard deviation | *p* | *I^2^* (%) |
| --- | --- | --- | --- | --- |
| ACR | –0.089 | 0.158 | 0.58 | 0 |
| ALIC | –0.073 | 0.194 | 0.71 | 33.1 |
| Average AD | 0.073 | 0.162 | 0.65 | 0 |
| BCC | 0.066 | 0.166 | 0.69 | 0 |
| CC | –0.075 | 0.163 | 0.64 | 0 |
| CGC | –0.104 | 0.173 | 0.55 | 0 |
| CGH | –0.157 | 0.162 | 0.33 | 0 |
| CR | 0.333 | 0.156 | 3.3 × 10^–2^ | 0 |
| CST | –0.091 | 0.249 | 0.71 | 47.7 |
| EC | 0.046 | 0.166 | 0.78 | 0 |
| FX | 0.689 | 0.160 | **1.7 × 10^–5^** | 0 |
| FXST | –0.198 | 0.166 | 0.23 | 0 |
| GCC | –0.163 | 0.191 | 0.39 | 24.8 |
| IC | 0.181 | 0.150 | 0.23 | 0 |
| IFO | 0.468 | 0.165 | 4.5 × 10^–3^ | 0 |
| PCR | 0.473 | 0.159 | 2.9 × 10^–3^ | 0 |
| PLIC | 0.201 | 0.157 | 0.20 | 0 |
| PTR | –0.171 | 0.332 | 0.61 | 70.1 |
| RLIC | 0.326 | 0.161 | 4.3 × 10^–2^ | 0 |
| SCC | –0.233 | 0.228 | 0.31 | 38.9 |
| SCR | 0.583 | 0.159 | **2.5 × 10^–4^** | 0 |
| SFO | 0.047 | 0.162 | 0.77 | 9.8 |
| SLF | 0.244 | 0.168 | 0.15 | 0 |
| SS | –0.106 | 0.170 | 0.53 | 0 |
| UNC | 0.349 | 0.144 | 1.5 × 10^–2^ | 0 |

**Supplementary Table 41** Mega-analysis results of differences in radial diffusivity (RD) between patients with schizophrenia and individuals with autism spectrum disorder.

| Region of interest | Cohen’s *d* | Standard deviation | *p* | *I^2^* (%) |
| --- | --- | --- | --- | --- |
| ACR | 0.288 | 0.194 | 0.14 | 33.0 |
| ALIC | 0.176 | 0.225 | 0.43 | 44.8 |
| Average RD | 0.252 | 0.167 | 0.13 | 7.4 |
| BCC | 0.141 | 0.164 | 0.39 | 0 |
| CC | 0.097 | 0.175 | 0.58 | 15.6 |
| CGC | –0.073 | 0.169 | 0.67 | 0 |
| CGH | –0.328 | 0.288 | 0.25 | 63.0 |
| CR | 0.086 | 0.184 | 0.64 | 23.0 |
| CST | 0.020 | 0.237 | 0.93 | 46.4 |
| EC | –0.081 | 0.169 | 0.63 | 0 |
| FX | 0.651 | 0.159 | **4.4 × 10^–5^** | 0 |
| FXST | 0.194 | 0.174 | 0.26 | 5.1 |
| GCC | 0.140 | 0.237 | 0.56 | 55.2 |
| IC | –0.115 | 0.189 | 0.54 | 18.2 |
| IFO | –0.020 | 0.173 | 0.91 | 0 |
| PCR | –0.017 | 0.161 | 0.92 | 0 |
| PLIC | –0.491 | 0.219 | 2.5 × 10^–2^ | 34.1 |
| PTR | 0.015 | 0.227 | 0.95 | 46.7 |
| RLIC | 0.067 | 0.171 | 0.70 | 0 |
| SCC | –0.100 | 0.176 | 0.57 | 10.7 |
| SCR | –0.153 | 0.166 | 0.35 | 0 |
| SFO | 0.137 | 0.165 | 0.41 | 0 |
| SLF | 0.005 | 0.177 | 0.98 | 8.0 |
| SS | 0.165 | 0.231 | 0.48 | 46.4 |
| UNC | –0.174 | 0.214 | 0.42 | 45.2 |

**Supplementary Table 42** Mega-analysis results of differences in fractional anisotropy (FA) between patients with schizophrenia and patients with major depressive disorder.

| Region of interest | Cohen’s *d* | Standard deviation | *p* | *I^2^* (%) |
| --- | --- | --- | --- | --- |
| ACR | –0.268 | 0.119 | 2.4 × 10^–2^ | 25.2 |
| ALIC | –0.401 | 0.096 | **2.8 × 10^–5^** | 0 |
| Average FA | –0.280 | 0.101 | 5.7 × 10^–3^ | 10.8 |
| BCC | –0.258 | 0.094 | 5.8 × 10^–3^ | 0 |
| CC | –0.286 | 0.092 | **2.0 × 10^–3^** | 0 |
| CGC | –0.085 | 0.137 | 0.54 | 35.4 |
| CGH | –0.163 | 0.097 | 9.3 × 10^–2^ | 0 |
| CR | –0.190 | 0.092 | 3.9 × 10^–2^ | 0 |
| CST | –0.010 | 0.106 | 0.93 | 7.2 |
| EC | –0.273 | 0.141 | 5.3 × 10^–2^ | 39.6 |
| FX | –0.251 | 0.089 | 4.9 × 10^–3^ | 0 |
| FXST | –0.176 | 0.094 | 6.1 × 10^–2^ | 0 |
| GCC | –0.169 | 0.091 | 6.3 × 10^–2^ | 0 |
| IC | –0.254 | 0.097 | 8.9 × 10^–3^ | 0 |
| IFO | –0.050 | 0.100 | 0.61 | 0 |
| PCR | –0.123 | 0.114 | 0.28 | 16.1 |
| PLIC | –0.057 | 0.125 | 0.65 | 23.5 |
| PTR | –0.134 | 0.094 | 0.15 | 0 |
| RLIC | –0.178 | 0.098 | 6.9 × 10^–2^ | 0 |
| SCC | –0.261 | 0.097 | 7.3 × 10^–3^ | 0 |
| SCR | –0.072 | 0.156 | 0.65 | 48.3 |
| SFO | –0.313 | 0.095 | **1.0 × 10^–3^** | 1.9 |
| SLF | –0.136 | 0.097 | 0.16 | 0 |
| SS | –0.390 | 0.138 | 4.6 × 10^–3^ | 36.9 |
| UNC | –0.161 | 0.142 | 0.26 | 38.2 |

**Supplementary Table 43** Mega-analysis results of differences in mean diffusivity (MD) between patients with schizophrenia and patients with major depressive disorder.

| Region of interest | Cohen’s *d* | Standard deviation | *p* | *I^2^* (%) |
| --- | --- | --- | --- | --- |
| ACR | 0.403 | 0.204 | 4.9 × 10^–2^ | 70.5 |
| ALIC | 0.051 | 0.089 | 0.57 | 0 |
| Average MD | 0.404 | 0.153 | 8.2 × 10^–3^ | 52.5 |
| BCC | 0.310 | 0.092 | **8.0 × 10^–4^** | 0 |
| CC | 0.329 | 0.090 | **2.5 × 10^–4^** | 0 |
| CGC | 0.307 | 0.094 | **1.2 × 10^–3^** | 0 |
| CGH | 0.168 | 0.094 | 7.4 × 10^–2^ | 0 |
| CR | 0.395 | 0.189 | 3.6 × 10^–2^ | 64.9 |
| CST | 0.002 | 0.099 | 0.99 | 0 |
| EC | 0.086 | 0.095 | 0.36 | 7.7 |
| FX | 0.255 | 0.116 | 2.8 × 10^–2^ | 28.0 |
| FXST | –0.004 | 0.090 | 0.96 | 0 |
| GCC | 0.336 | 0.180 | 6.2 × 10^–2^ | 62.9 |
| IC | 0.158 | 0.141 | 0.26 | 42.1 |
| IFO | 0.176 | 0.191 | 0.36 | 63.9 |
| PCR | 0.317 | 0.170 | 6.2 × 10^–2^ | 55.9 |
| PLIC | 0.060 | 0.106 | 0.57 | 8.3 |
| PTR | 0.200 | 0.140 | 0.15 | 39.5 |
| RLIC | 0.208 | 0.154 | 0.18 | 47.4 |
| SCC | 0.318 | 0.093 | **6.2 × 10^–4^** | 0 |
| SCR | 0.328 | 0.168 | 5.1 × 10^–2^ | 55.5 |
| SFO | 0.327 | 0.218 | 0.13 | 74.3 |
| SLF | 0.381 | 0.163 | 1.9 × 10^–2^ | 53.2 |
| SS | 0.380 | 0.225 | 9.1 × 10^–2^ | 75.1 |
| UNC | 0.205 | 0.133 | 0.12 | 36.8 |

**Supplementary Table 44** Mega-analysis results of differences in axial diffusivity (AD) between patients with schizophrenia and patients with major depressive disorder.

| Region of interest | Cohen’s *d* | Standard deviation | *p* | *I^2^* (%) |
| --- | --- | --- | --- | --- |
| ACR | 0.184 | 0.214 | 0.39 | 72.3 |
| ALIC | –0.191 | 0.090 | 3.4 × 10^–2^ | 0 |
| Average AD | 0.329 | 0.147 | 2.6 × 10^–2^ | 51.0 |
| BCC | 0.177 | 0.096 | 6.6 × 10^–2^ | 0 |
| CC | 0.178 | 0.095 | 6.1 × 10^–2^ | 0 |
| CGC | 0.185 | 0.098 | 5.8 × 10^–2^ | 0 |
| CGH | 0.045 | 0.112 | 0.69 | 17.6 |
| CR | 0.263 | 0.168 | 0.12 | 56.2 |
| CST | –0.041 | 0.100 | 0.68 | 0 |
| EC | –0.027 | 0.085 | 0.75 | 0 |
| FX | 0.211 | 0.113 | 6.2 × 10^–2^ | 24.7 |
| FXST | –0.111 | 0.091 | 0.22 | 0 |
| GCC | 0.265 | 0.200 | 0.18 | 68.1 |
| IC | –0.001 | 0.105 | 0.99 | 17.0 |
| IFO | –0.099 | 0.097 | 0.31 | 0 |
| PCR | 0.231 | 0.150 | 0.12 | 45.4 |
| PLIC | 0.086 | 0.141 | 0.54 | 39.9 |
| PTR | 0.011 | 0.098 | 0.91 | 0 |
| RLIC | 0.053 | 0.089 | 0.55 | 0 |
| SCC | 0.157 | 0.094 | 9.4 × 10^–2^ | 0 |
| SCR | 0.231 | 0.133 | 8.2 × 10^–2^ | 34.0 |
| SFO | –0.018 | 0.189 | 0.92 | 66.0 |
| SLF | 0.204 | 0.103 | 4.7 × 10^–2^ | 9.1 |
| SS | 0.013 | 0.128 | 0.92 | 29.4 |
| UNC | 0.149 | 0.091 | 0.10 | 0 |

**Supplementary Table 45** Mega-analysis results of differences in radial diffusivity (RD) between patients with schizophrenia and patients with major depressive disorder.

| Region of interest | Cohen’s *d* | Standard deviation | *p* | *I^2^* (%) |
| --- | --- | --- | --- | --- |
| ACR | 0.365 | 0.144 | 1.1 × 10^–2^ | 44.9 |
| ALIC | 0.199 | 0.101 | 4.8 × 10^–2^ | 8.2 |
| Average RD | 0.385 | 0.142 | 6.5 × 10^–3^ | 47.4 |
| BCC | 0.316 | 0.091 | **5.4 × 10^–4^** | 0 |
| CC | 0.339 | 0.089 | **1.5 × 10^–4^** | 0 |
| CGC | 0.297 | 0.093 | **1.4 × 10^–3^** | 0 |
| CGH | 0.204 | 0.095 | 3.3 × 10^–2^ | 0 |
| CR | 0.320 | 0.151 | 3.4 × 10^–2^ | 48.2 |
| CST | 0.029 | 0.099 | 0.77 | 0 |
| EC | 0.196 | 0.134 | 0.14 | 38.7 |
| FX | 0.257 | 0.106 | 1.5 × 10^–2^ | 19.5 |
| FXST | 0.059 | 0.094 | 0.53 | 4.1 |
| GCC | 0.193 | 0.090 | 3.3 × 10^–2^ | 0.7 |
| IC | 0.166 | 0.134 | 0.22 | 33.5 |
| IFO | 0.101 | 0.153 | 0.51 | 44.1 |
| PCR | 0.226 | 0.140 | 0.11 | 38.5 |
| PLIC | –0.005 | 0.105 | 0.96 | 6.4 |
| PTR | 0.249 | 0.147 | 9.1 × 10^–2^ | 45.7 |
| RLIC | 0.167 | 0.136 | 0.22 | 33.6 |
| SCC | 0.313 | 0.095 | **9.7 × 10^–4^** | 0 |
| SCR | 0.201 | 0.154 | 0.19 | 47.4 |
| SFO | 0.403 | 0.192 | 3.6 × 10^–2^ | 66.2 |
| SLF | 0.257 | 0.117 | 2.8 × 10^–2^ | 21.8 |
| SS | 0.430 | 0.222 | 5.3 × 10^–2^ | 75.0 |
| UNC | 0.182 | 0.143 | 0.20 | 40.7 |

**Supplementary Table 46** Mega-analysis results of differences in fractional anisotropy (FA) between patients with bipolar disorder and individuals with autism spectrum disorder.

| Region of interest | Cohen’s *d* | Standard deviation | *p* | *I^2^* (%) |
| --- | --- | --- | --- | --- |
| ACR | 0.228 | 0.352 | 0.52 | 0 |
| ALIC | 0.185 | 0.428 | 0.67 | 0 |
| Average FA | 0.222 | 0.342 | 0.52 | 0 |
| BCC | 0.521 | 0.361 | 0.15 | 0 |
| CC | 0.576 | 0.355 | 0.10 | 0 |
| CGC | 0.108 | 0.382 | 0.78 | 0 |
| CGH | –0.423 | 0.397 | 0.29 | 0 |
| CR | 0.503 | 0.367 | 0.17 | 0 |
| CST | 0.297 | 0.435 | 0.49 | 0 |
| EC | 0.113 | 0.394 | 0.78 | 0 |
| FX | –0.060 | 0.330 | 0.86 | 0 |
| FXST | –0.255 | 0.717 | 0.72 | 68.7 |
| GCC | 0.352 | 0.341 | 0.30 | 0 |
| IC | 0.670 | 0.391 | 8.7 × 10^–2^ | 0 |
| IFO | 0.049 | 0.417 | 0.91 | 0 |
| PCR | 0.488 | 0.385 | 0.20 | 0 |
| PLIC | 0.799 | 0.406 | 4.9 × 10^–2^ | 0 |
| PTR | 0.181 | 0.363 | 0.62 | 0 |
| RLIC | 0.524 | 0.393 | 0.18 | 0 |
| SCC | 0.624 | 0.394 | 0.11 | 0 |
| SCR | 0.740 | 0.409 | 7.0 × 10^–2^ | 0 |
| SFO | –0.201 | 0.442 | 0.65 | 11.8 |
| SLF | 0.546 | 0.368 | 0.14 | 0 |
| SS | 0.139 | 0.368 | 0.71 | 0 |
| UNC | –0.622 | 0.376 | 9.8 × 10^–2^ | 0 |

**Supplementary Table 47** Mega-analysis results of differences in mean diffusivity (MD) between patients with bipolar disorder and individuals with autism spectrum disorder.

| Region of interest | Cohen’s *d* | Standard deviation | *p* | *I^2^* (%) |
| --- | --- | --- | --- | --- |
| ACR | –0.495 | 0.341 | 0.15 | 0 |
| ALIC | –0.188 | 0.362 | 0.60 | 0 |
| Average MD | –0.273 | 0.341 | 0.42 | 0 |
| BCC | –0.223 | 0.333 | 0.50 | 0 |
| CC | –0.350 | 0.336 | 0.30 | 0 |
| CGC | –0.674 | 0.427 | 0.11 | 9.5 |
| CGH | –0.041 | 0.423 | 0.92 | 0 |
| CR | –0.466 | 0.333 | 0.16 | 0 |
| CST | –0.233 | 0.408 | 0.57 | 2.5 |
| EC | –0.592 | 0.373 | 0.11 | 0 |
| FX | –0.132 | 0.325 | 0.68 | 0 |
| FXST | –0.565 | 0.408 | 0.17 | 0 |
| GCC | –0.431 | 0.346 | 0.21 | 0 |
| IC | –0.379 | 0.384 | 0.32 | 0 |
| IFO | 0.134 | 0.436 | 0.76 | 0 |
| PCR | –0.486 | 0.328 | 0.14 | 0 |
| PLIC | –0.297 | 0.392 | 0.45 | 0 |
| PTR | –0.402 | 0.415 | 0.33 | 18.9 |
| RLIC | –0.410 | 0.412 | 0.32 | 0 |
| SCC | –0.310 | 0.381 | 0.42 | 0 |
| SCR | –0.321 | 0.345 | 0.35 | 0 |
| SFO | –0.050 | 0.366 | 0.89 | 0 |
| SLF | –0.595 | 0.362 | 0.10 | 0 |
| SS | –0.395 | 0.400 | 0.32 | 0 |
| UNC | –0.408 | 0.429 | 0.34 | 27.1 |

**Supplementary Table 48** Mega-analysis results of differences in axial diffusivity (AD) between patients with bipolar disorder and individuals with autism spectrum disorder.

| Region of interest | Cohen’s *d* | Standard deviation | *p* | *I^2^* (%) |
| --- | --- | --- | --- | --- |
| ACR | –0.328 | 0.385 | 0.39 | 0 |
| ALIC | –0.157 | 0.379 | 0.68 | 2.8 |
| Average AD | –0.158 | 0.415 | 0.70 | 15.3 |
| BCC | 0.267 | 0.432 | 0.54 | 0 |
| CC | 0.138 | 0.414 | 0.74 | 0 |
| CGC | –0.266 | 0.429 | 0.54 | 0 |
| CGH | –0.361 | 0.433 | 0.40 | 0 |
| CR | –0.090 | 0.352 | 0.80 | 0 |
| CST | –0.134 | 0.554 | 0.81 | 50.5 |
| EC | –0.695 | 0.458 | 0.13 | 24.4 |
| FX | –0.136 | 0.341 | 0.69 | 0 |
| FXST | –0.617 | 0.371 | 9.6 × 10^–2^ | 0 |
| GCC | –0.101 | 0.403 | 0.80 | 0 |
| IC | 0.061 | 0.374 | 0.87 | 0 |
| IFO | 0.063 | 0.393 | 0.87 | 0 |
| PCR | –0.145 | 0.348 | 0.68 | 0 |
| PLIC | 0.315 | 0.391 | 0.42 | 0 |
| PTR | –0.246 | 0.427 | 0.56 | 0 |
| RLIC | –0.143 | 0.397 | 0.72 | 0 |
| SCC | 0.161 | 0.464 | 0.73 | 25.2 |
| SCR | 0.200 | 0.362 | 0.58 | 0 |
| SFO | –0.060 | 0.576 | 0.92 | 57.8 |
| SLF | –0.135 | 0.367 | 0.71 | 0 |
| SS | –0.394 | 0.447 | 0.38 | 0 |
| UNC | –0.819 | 0.537 | 0.13 | 62.1 |

**Supplementary Table 49** Mega-analysis results of differences in radial diffusivity (RD) between patients with bipolar disorder and individuals with autism spectrum disorder.

| Region of interest | Cohen’s *d* | Standard deviation | *p* | *I^2^* (%) |
| --- | --- | --- | --- | --- |
| ACR | –0.423 | 0.338 | 0.21 | 0 |
| ALIC | –0.133 | 0.394 | 0.73 | 0 |
| Average RD | –0.266 | 0.325 | 0.41 | 0 |
| BCC | –0.356 | 0.329 | 0.28 | 0 |
| CC | –0.472 | 0.326 | 0.15 | 0 |
| CGC | –0.444 | 0.363 | 0.22 | 0 |
| CGH | 0.224 | 0.400 | 0.58 | 0 |
| CR | –0.559 | 0.343 | 0.10 | 0 |
| CST | –0.212 | 0.423 | 0.62 | 0 |
| EC | –0.326 | 0.387 | 0.40 | 0 |
| FX | –0.117 | 0.320 | 0.72 | 0 |
| FXST | 0.040 | 0.969 | 0.97 | 83.7 |
| GCC | –0.479 | 0.310 | 0.12 | 0 |
| IC | –0.557 | 0.393 | 0.16 | 0 |
| IFO | 0.129 | 0.440 | 0.77 | 0 |
| PCR | –0.594 | 0.354 | 9.3 × 10^–2^ | 0 |
| PLIC | –0.718 | 0.410 | 8.0 × 10^–2^ | 0 |
| PTR | –0.323 | 0.356 | 0.36 | 0 |
| RLIC | –0.419 | 0.400 | 0.29 | 0 |
| SCC | –0.520 | 0.382 | 0.17 | 0 |
| SCR | –0.594 | 0.373 | 0.11 | 0 |
| SFO | 0.097 | 0.395 | 0.81 | 0 |
| SLF | –0.624 | 0.369 | 9.1 × 10^–2^ | 0 |
| SS | –0.265 | 0.374 | 0.48 | 0 |
| UNC | 0.183 | 0.393 | 0.64 | 0 |

**Supplementary Table 50** Mega-analysis results of differences in fractional anisotropy (FA) between patients with bipolar disorder and patients with major depressive disorder.

| Region of interest | Cohen’s *d* | Standard deviation | *p* | *I^2^* (%) |
| --- | --- | --- | --- | --- |
| ACR | –0.054 | 0.082 | 0.51 | 0 |
| ALIC | –0.107 | 0.102 | 0.29 | 14.5 |
| Average FA | –0.140 | 0.108 | 0.19 | 31.3 |
| BCC | –0.249 | 0.105 | 1.7 × 10^–2^ | 20.0 |
| CC | –0.241 | 0.097 | 1.3 × 10^–2^ | 12.1 |
| CGC | –0.279 | 0.129 | 3.1 × 10^–2^ | 44.0 |
| CGH | –0.092 | 0.207 | 0.66 | 77.1 |
| CR | –0.029 | 0.085 | 0.74 | 0 |
| CST | –0.095 | 0.089 | 0.28 | 0 |
| EC | –0.048 | 0.092 | 0.60 | 6.1 |
| FX | –0.215 | 0.081 | 8.2 × 10^–3^ | 0 |
| FXST | –0.199 | 0.084 | 1.8 × 10^–2^ | 0 |
| GCC | –0.157 | 0.083 | 5.9 × 10^–2^ | 0 |
| IC | –0.098 | 0.102 | 0.34 | 12.4 |
| IFO | 0.084 | 0.092 | 0.36 | 0 |
| PCR | 0.067 | 0.105 | 0.52 | 16.0 |
| PLIC | –0.094 | 0.101 | 0.35 | 9.8 |
| PTR | –0.054 | 0.088 | 0.54 | 0 |
| RLIC | 0.045 | 0.119 | 0.70 | 29.4 |
| SCC | –0.185 | 0.129 | 0.15 | 37.8 |
| SCR | –0.025 | 0.089 | 0.78 | 0 |
| SFO | –0.125 | 0.108 | 0.25 | 22.0 |
| SLF | –0.143 | 0.101 | 0.16 | 13.8 |
| SS | 0.069 | 0.087 | 0.43 | 0 |
| UNC | –0.061 | 0.134 | 0.65 | 45.0 |

**Supplementary Table 51** Mega-analysis results of differences in mean diffusivity (MD) between patients with bipolar disorder and patients with major depressive disorder.

| Region of interest | Cohen’s *d* | Standard deviation | *p* | *I^2^* (%) |
| --- | --- | --- | --- | --- |
| ACR | 0.103 | 0.081 | 0.20 | 0 |
| ALIC | 0.009 | 0.076 | 0.91 | 0 |
| Average MD | 0.098 | 0.074 | 0.18 | 0 |
| BCC | 0.201 | 0.081 | 1.4 × 10^–2^ | 0 |
| CC | 0.210 | 0.080 | 8.7 × 10^–3^ | 0 |
| CGC | 0.105 | 0.083 | 0.20 | 0 |
| CGH | 0.050 | 0.088 | 0.57 | 0 |
| CR | 0.101 | 0.080 | 0.21 | 0 |
| CST | 0.120 | 0.110 | 0.27 | 20.9 |
| EC | –0.016 | 0.074 | 0.82 | 0 |
| FX | 0.237 | 0.075 | **1.6 × 10^–3^** | 0 |
| FXST | 0.099 | 0.081 | 0.23 | 0 |
| GCC | 0.209 | 0.080 | 8.7 × 10^–3^ | 0 |
| IC | 0.080 | 0.082 | 0.33 | 0 |
| IFO | –0.012 | 0.144 | 0.93 | 49.1 |
| PCR | 0.086 | 0.085 | 0.31 | 0 |
| PLIC | 0.138 | 0.089 | 0.12 | 0 |
| PTR | 0.001 | 0.087 | 0.99 | 0 |
| RLIC | 0.049 | 0.085 | 0.57 | 0 |
| SCC | 0.126 | 0.134 | 0.35 | 46.0 |
| SCR | 0.081 | 0.081 | 0.32 | 0 |
| SFO | 0.124 | 0.079 | 0.11 | 0 |
| SLF | 0.111 | 0.085 | 0.19 | 0 |
| SS | 0.035 | 0.082 | 0.67 | 0 |
| UNC | 0.069 | 0.093 | 0.46 | 12.6 |

**Supplementary Table 52** Mega-analysis results of differences in axial diffusivity (AD) between patients with bipolar disorder and patients with major depressive disorder.

| Region of interest | Cohen’s *d* | Standard deviation | *p* | *I^2^* (%) |
| --- | --- | --- | --- | --- |
| ACR | 0.093 | 0.107 | 0.38 | 24.4 |
| ALIC | –0.010 | 0.079 | 0.90 | 0 |
| Average AD | 0.046 | 0.075 | 0.54 | 0 |
| BCC | 0.048 | 0.087 | 0.58 | 0 |
| CC | 0.065 | 0.085 | 0.45 | 0 |
| CGC | –0.123 | 0.162 | 0.45 | 61.5 |
| CGH | 0.137 | 0.098 | 0.16 | 10.3 |
| CR | 0.149 | 0.112 | 0.18 | 36.0 |
| CST | 0.066 | 0.165 | 0.69 | 59.3 |
| EC | –0.111 | 0.134 | 0.41 | 57.9 |
| FX | 0.245 | 0.094 | 8.8 × 10^–3^ | 19.7 |
| FXST | 0.001 | 0.085 | 0.99 | 0 |
| GCC | 0.162 | 0.104 | 0.12 | 21.1 |
| IC | 0.133 | 0.130 | 0.31 | 52.0 |
| IFO | 0.058 | 0.128 | 0.65 | 37.0 |
| PCR | 0.145 | 0.084 | 8.2 × 10^–2^ | 0 |
| PLIC | 0.240 | 0.140 | 8.6 × 10^–2^ | 50.9 |
| PTR | –0.076 | 0.091 | 0.41 | 0 |
| RLIC | 0.050 | 0.124 | 0.69 | 44.5 |
| SCC | –0.026 | 0.092 | 0.78 | 5.0 |
| SCR | 0.128 | 0.108 | 0.24 | 33.4 |
| SFO | 0.084 | 0.100 | 0.40 | 26.5 |
| SLF | 0.044 | 0.086 | 0.61 | 0 |
| SS | 0.030 | 0.139 | 0.83 | 51.5 |
| UNC | 0.063 | 0.084 | 0.45 | 0 |

**Supplementary Table 53** Mega-analysis results of differences in radial diffusivity (RD) between patients with bipolar disorder and patients with major depressive disorder.

| Region of interest | Cohen’s *d* | Standard deviation | *p* | *I^2^* (%) |
| --- | --- | --- | --- | --- |
| ACR | 0.104 | 0.080 | 0.19 | 0 |
| ALIC | 0.022 | 0.079 | 0.78 | 0 |
| Average RD | 0.113 | 0.074 | 0.13 | 0 |
| BCC | 0.219 | 0.084 | 9.5 × 10^–3^ | 2.1 |
| CC | 0.221 | 0.082 | 6.9 × 10^–3^ | 0 |
| CGC | 0.177 | 0.082 | 3.1 × 10^–2^ | 0 |
| CGH | 0.038 | 0.171 | 0.83 | 65.2 |
| CR | 0.088 | 0.081 | 0.28 | 0 |
| CST | 0.125 | 0.090 | 0.16 | 0 |
| EC | 0.017 | 0.076 | 0.82 | 0 |
| FX | 0.236 | 0.075 | **1.6 × 10^–3^** | 0 |
| FXST | 0.165 | 0.081 | 4.0 × 10^–2^ | 0 |
| GCC | 0.179 | 0.080 | 2.6 × 10^–2^ | 0 |
| IC | 0.054 | 0.085 | 0.53 | 0 |
| IFO | –0.070 | 0.119 | 0.56 | 28.5 |
| PCR | 0.026 | 0.087 | 0.77 | 0 |
| PLIC | 0.079 | 0.091 | 0.38 | 0 |
| PTR | 0.043 | 0.087 | 0.62 | 0 |
| RLIC | 0.029 | 0.088 | 0.75 | 0 |
| SCC | 0.168 | 0.136 | 0.21 | 46.2 |
| SCR | 0.058 | 0.084 | 0.49 | 0 |
| SFO | 0.131 | 0.082 | 0.11 | 0 |
| SLF | 0.136 | 0.086 | 0.11 | 0 |
| SS | 0.029 | 0.082 | 0.73 | 0 |
| UNC | 0.066 | 0.101 | 0.51 | 18.9 |

**Supplementary Table 54** Mega-analysis results of differences in fractional anisotropy (FA) between individuals with autism spectrum disorder and patients with major depressive disorder.

| Region of interest | Cohen’s *d* | Standard deviation | *p* | *I^2^* (%) |
| --- | --- | --- | --- | --- |
| ACR | –0.002 | 0.370 | 1.00 | 0 |
| ALIC | –0.533 | 0.372 | 0.15 | 0 |
| Average FA | –0.249 | 0.263 | 0.34 | 0 |
| BCC | –0.087 | 0.416 | 0.83 | 0 |
| CC | –0.181 | 0.385 | 0.64 | 0 |
| CGC | –0.229 | 0.357 | 0.52 | 0 |
| CGH | –0.482 | 0.493 | 0.33 | 30.1 |
| CR | –0.224 | 0.344 | 0.51 | 0 |
| CST | –0.356 | 0.402 | 0.38 | 0 |
| EC | –0.488 | 0.349 | 0.16 | 0 |
| FX | 0.184 | 0.389 | 0.64 | 0 |
| FXST | –0.089 | 0.355 | 0.80 | 0 |
| GCC | –0.095 | 0.333 | 0.78 | 0 |
| IC | –0.590 | 0.467 | 0.21 | 28.5 |
| IFO | 0.170 | 0.417 | 0.68 | 0 |
| PCR | –0.409 | 0.343 | 0.23 | 0 |
| PLIC | –0.622 | 0.374 | 9.6 × 10^–2^ | 0 |
| PTR | 0.331 | 1.040 | 0.75 | 91.2 |
| RLIC | –0.233 | 0.605 | 0.70 | 47.2 |
| SCC | 0.020 | 0.737 | 0.98 | 71.8 |
| SCR | –0.297 | 0.355 | 0.40 | 0 |
| SFO | 0.013 | 0.383 | 0.97 | 0 |
| SLF | 0.099 | 0.944 | 0.92 | 85.1 |
| SS | –0.167 | 0.708 | 0.81 | 71.4 |
| UNC | –0.807 | 0.701 | 0.25 | 68.5 |

**Supplementary Table 55** Mega-analysis results of differences in mean diffusivity (MD) between individuals with autism spectrum disorder and patients with major depressive disorder.

| Region of interest | Cohen’s *d* | Standard deviation | *p* | *I^2^* (%) |
| --- | --- | --- | --- | --- |
| ACR | 0.479 | 0.348 | 0.17 | 0 |
| ALIC | –0.084 | 0.651 | 0.90 | 55.2 |
| Average MD | 0.506 | 0.375 | 0.18 | 0 |
| BCC | 0.380 | 0.404 | 0.35 | 0 |
| CC | 0.855 | 0.380 | 2.4 × 10^–2^ | 0 |
| CGC | 0.775 | 0.414 | 6.1 × 10^–2^ | 0 |
| CGH | 0.248 | 0.375 | 0.51 | 0 |
| CR | 0.492 | 0.356 | 0.17 | 0 |
| CST | 0.004 | 0.400 | 0.99 | 0 |
| EC | 0.261 | 0.354 | 0.46 | 0 |
| FX | –0.159 | 0.385 | 0.68 | 0 |
| FXST | 0.106 | 0.734 | 0.89 | 66.2 |
| GCC | 0.935 | 0.348 | 7.3 × 10^–3^ | 0 |
| IC | –0.027 | 0.528 | 0.96 | 44.0 |
| IFO | –0.076 | 0.405 | 0.85 | 0 |
| PCR | 0.478 | 0.344 | 0.17 | 0 |
| PLIC | –0.259 | 0.447 | 0.56 | 44.9 |
| PTR | 0.423 | 0.423 | 0.32 | 42.3 |
| RLIC | 0.186 | 0.395 | 0.64 | 0 |
| SCC | 1.042 | 0.382 | 6.4 × 10^–3^ | 0 |
| SCR | 0.357 | 0.379 | 0.35 | 0 |
| SFO | –0.216 | 0.388 | 0.58 | 0 |
| SLF | 0.740 | 0.539 | 0.17 | 39.6 |
| SS | 0.582 | 0.362 | 0.11 | 0 |
| UNC | 0.458 | 0.321 | 0.15 | 0 |

**Supplementary Table 56** Mega-analysis results of differences in axial diffusivity (AD) between individuals with autism spectrum disorder and patients with major depressive disorder.

| Region of interest | Cohen’s *d* | Standard deviation | *p* | *I^2^* (%) |
| --- | --- | --- | --- | --- |
| ACR | 0.403 | 0.392 | 0.30 | 0 |
| ALIC | –0.255 | 0.409 | 0.53 | 0 |
| Average AD | 0.394 | 0.398 | 0.32 | 0 |
| BCC | 0.364 | 0.422 | 0.39 | 0 |
| CC | 0.727 | 0.412 | 7.8 × 10^–2^ | 0 |
| CGC | 0.118 | 0.410 | 0.77 | 0 |
| CGH | –0.126 | 0.380 | 0.74 | 0 |
| CR | 0.312 | 0.421 | 0.46 | 0 |
| CST | –0.017 | 0.383 | 0.96 | 0 |
| EC | –0.290 | 0.616 | 0.64 | 37.8 |
| FX | –0.302 | 0.388 | 0.44 | 0 |
| FXST | 0.172 | 0.457 | 0.71 | 7.1 |
| GCC | 1.020 | 0.613 | 9.6 × 10^–2^ | 46.9 |
| IC | –0.403 | 0.374 | 0.28 | 0 |
| IFO | 0.122 | 0.398 | 0.76 | 0 |
| PCR | 0.426 | 0.386 | 0.27 | 0 |
| PLIC | –0.602 | 0.358 | 9.3 × 10^–2^ | 0 |
| PTR | 0.482 | 0.396 | 0.22 | 0 |
| RLIC | –0.076 | 0.396 | 0.85 | 0 |
| SCC | 0.810 | 0.413 | 5.0 × 10^–2^ | 0 |
| SCR | –0.035 | 0.411 | 0.93 | 0 |
| SFO | –0.295 | 0.408 | 0.47 | 0 |
| SLF | 0.574 | 0.397 | 0.15 | 0 |
| SS | 0.309 | 0.403 | 0.44 | 0 |
| UNC | –0.577 | 1.163 | 0.62 | 84.8 |

**Supplementary Table 57** Mega-analysis results of differences in radial diffusivity (RD) between individuals with autism spectrum disorder and patients with major depressive disorder.

| Region of interest | Cohen’s *d* | Standard deviation | *p* | *I^2^* (%) |
| --- | --- | --- | --- | --- |
| ACR | 0.300 | 0.348 | 0.39 | 0 |
| ALIC | 0.144 | 0.641 | 0.82 | 63.9 |
| Average RD | 0.435 | 0.331 | 0.19 | 0 |
| BCC | 0.254 | 0.408 | 0.53 | 0 |
| CC | 0.524 | 0.377 | 0.17 | 0 |
| CGC | 0.638 | 0.348 | 6.7 × 10^–2^ | 0 |
| CGH | 0.508 | 0.484 | 0.29 | 31.8 |
| CR | 0.393 | 0.331 | 0.23 | 4.0 |
| CST | –0.010 | 0.402 | 0.98 | 0 |
| EC | 0.350 | 0.343 | 0.31 | 0 |
| FX | –0.080 | 0.383 | 0.83 | 0 |
| FXST | 0.020 | 0.485 | 0.97 | 58.2 |
| GCC | 0.499 | 0.318 | 0.12 | 0 |
| IC | 0.181 | 0.614 | 0.77 | 67.2 |
| IFO | –0.203 | 0.412 | 0.62 | 0 |
| PCR | 0.372 | 0.350 | 0.29 | 8.4 |
| PLIC | 0.305 | 0.422 | 0.47 | 28.7 |
| PTR | 0.027 | 0.729 | 0.97 | 87.2 |
| RLIC | 0.181 | 0.479 | 0.71 | 29.3 |
| SCC | 0.420 | 0.665 | 0.53 | 67.5 |
| SCR | 0.436 | 0.360 | 0.23 | 9.5 |
| SFO | –0.101 | 0.383 | 0.79 | 0 |
| SLF | 0.333 | 0.740 | 0.65 | 74.7 |
| SS | 0.488 | 0.334 | 0.14 | 0 |
| UNC | 0.592 | 0.330 | 7.3 × 10^–2^ | 0 |

**Legends of Supplementary Table 34–57**: Bold means statistical significant [*p* < 0.002 (0.05/25)].

Abbreviations: ACR, anterior corona radiata; AD, axial diffusivity; ALIC, anterior limb of internal capsule; BCC, body of corpus callosum; CC, corpus callosum; CGC, cingulum (cingulate gyrus); CGH, cingulum (hippocampus); CR, corona radiata; CST, corticospinal tract; EC, external capsule; FA, fractional anisotropy; FX, fornix; FX/ST, fornix (crus)/stria terminalis; GCC, genu of corpus callosum; IC, internal capsule; IFO, inferior fronto-occipital fasciculus; MD, mean diffusivity; PCR, posterior corona radiata; PLIC, posterior limb of internal capsule; PTR, posterior thalamic radiation; RD, radial diffusivity; RLIC, retrolenticular part of internal capsule; SCC, splenium of corpus callosum; SCR, superior corona radiata; SFO, superior fronto-occipital fasciculus; SLF, superior longitudinal fasciculus; SS, sagittal stratum; UNC, uncinate fasciculus.

**Supplementary Table 58** Mega-analysis results of correlation between fractional anisotropy (FA) and duration of illness in patients with schizophrenia

| Region of interest | *β* | 95% CI-lower bound | 95% CI-upper bound | *p* | *I^2^* (%) |
| --- | --- | --- | --- | --- | --- |
| ACR | –0.315 | –0.441 | –0.177 | **1.4 × 10^–5^** | 67.9 |
| ALIC | –0.242 | –0.322 | –0.159 | **2.2 × 10^–8^** | 14.1 |
| Average FA | –0.230 | –0.355 | –0.097 | **8.3 × 10^–4^** | 63.1 |
| BCC | –0.260 | –0.342 | –0.174 | **8.2 × 10^–9^** | 20.1 |
| CC | –0.254 | –0.366 | –0.134 | **4.3 × 10^–5^** | 55.1 |
| CGC | –0.115 | –0.222 | –0.005 | 4.0 × 10^–2^ | 43.3 |
| CGH | 0.062 | –0.016 | 0.139 | 0.12 | 0 |
| CR | –0.295 | –0.414 | –0.166 | **1.3 × 10^–5^** | 62.8 |
| CST | 0.068 | –0.032 | 0.166 | 0.18 | 32.5 |
| EC | –0.175 | –0.272 | –0.075 | **6.7 × 10^–4^** | 34.9 |
| FX | –0.274 | –0.344 | –0.200 | **1.8 × 10^–12^** | 0 |
| FXST | –0.122 | –0.214 | –0.028 | 1.1 × 10^–2^ | 25.4 |
| GCC | –0.238 | –0.362 | –0.106 | **4.8 × 10^–4^** | 62.5 |
| IC | –0.200 | –0.297 | –0.098 | **1.4 × 10^–4^** | 37.3 |
| IFO | –0.051 | –0.139 | 0.039 | 0.27 | 19.0 |
| PCR | –0.253 | –0.382 | –0.114 | **4.3 × 10^–4^** | 66.4 |
| PLIC | –0.085 | –0.162 | –0.007 | 3.2 × 10^–2^ | 0 |
| PTR | –0.304 | –0.428 | –0.170 | **1.6 × 10^–5^** | 66.0 |
| RLIC | –0.195 | –0.327 | –0.056 | 6.3 × 10^–3^ | 65.4 |
| SCC | –0.119 | –0.222 | –0.014 | 2.7 × 10^–2^ | 39.1 |
| SCR | –0.184 | –0.281 | –0.083 | **3.8 × 10^–4^** | 35.8 |
| SFO | –0.262 | –0.359 | –0.160 | **8.6 × 10^–7^** | 40.3 |
| SLF | –0.195 | –0.288 | –0.097 | **1.1 × 10^–4^** | 32.1 |
| SS | –0.185 | –0.315 | –0.048 | 8.5 × 10^–3^ | 64.0 |
| UNC | –0.172 | –0.295 | –0.044 | 8.8 × 10^–3^ | 58.9 |

**Supplementary Table 59** Mega-analysis results of correlation between mean diffusivity (MD) and duration of illness in patients with schizophrenia

| Region of interest | *β* | 95% CI-lower bound | 95% CI-upper bound | *p* | *I^2^* (%) |
| --- | --- | --- | --- | --- | --- |
| ACR | 0.190 | 0.068 | 0.306 | 2.4 × 10^–3^ | 54.9 |
| ALIC | 0.008 | –0.137 | 0.152 | 0.92 | 67.2 |
| Average MD | 0.136 | –0.003 | 0.269 | 5.4 × 10^–2^ | 64.2 |
| BCC | 0.236 | 0.161 | 0.308 | **1.6 × 10^–9^** | 0 |
| CC | 0.244 | 0.157 | 0.327 | **6.4 × 10^–8^** | 19.7 |
| CGC | 0.066 | –0.053 | 0.182 | 0.28 | 50.9 |
| CGH | –0.113 | –0.241 | 0.019 | 9.3 × 10^–2^ | 60.3 |
| CR | 0.201 | 0.085 | 0.311 | **7.3 × 10^–4^** | 50.5 |
| CST | –0.064 | –0.151 | 0.024 | 0.15 | 16.5 |
| EC | 0.050 | –0.119 | 0.216 | 0.56 | 76.0 |
| FX | 0.326 | 0.254 | 0.394 | **2.1 × 10^–17^** | 0 |
| FXST | 0.003 | –0.093 | 0.100 | 0.95 | 28.5 |
| GCC | 0.207 | 0.104 | 0.306 | **1.0 × 10^–4^** | 39.2 |
| IC | 0.006 | –0.129 | 0.140 | 0.93 | 61.9 |
| IFO | –0.024 | –0.132 | 0.084 | 0.66 | 41.7 |
| PCR | 0.217 | 0.130 | 0.302 | **1.8 × 10^–6^** | 20.1 |
| PLIC | –0.028 | –0.136 | 0.080 | 0.61 | 42.1 |
| PTR | 0.213 | 0.106 | 0.315 | **1.1 × 10^–4^** | 43.0 |
| RLIC | 0.057 | –0.062 | 0.173 | 0.35 | 50.6 |
| SCC | 0.148 | 0.049 | 0.244 | 3.4 × 10^–3^ | 32.2 |
| SCR | 0.172 | 0.054 | 0.284 | 4.3 × 10^–3^ | 51.0 |
| SFO | 0.108 | –0.001 | 0.214 | 5.3 × 10^–2^ | 42.5 |
| SLF | 0.153 | 0.037 | 0.264 | 9.8 × 10^–3^ | 49.1 |
| SS | 0.158 | 0.052 | 0.261 | 3.7 × 10^–3^ | 41.1 |
| UNC | 0.271 | 0.165 | 0.371 | **1.1 × 10^–6^** | 44.6 |

**Supplementary Table 60** Mega-analysis results of correlation between axial diffusivity (AD) and duration of illness in patients with schizophrenia

| Region of interest | *β* | 95% CI-lower bound | 95% CI-upper bound | *p* | *I^2^* (%) |
| --- | --- | --- | --- | --- | --- |
| ACR | –0.022 | –0.173 | 0.130 | 0.78 | 70.3 |
| ALIC | –0.141 | –0.268 | –0.009 | 3.7 × 10^–2^ | 60.6 |
| Average AD | –0.055 | –0.200 | 0.092 | 0.46 | 68.1 |
| BCC | 0.071 | –0.007 | 0.148 | 7.5 × 10^–2^ | 0 |
| CC | 0.079 | 0.001 | 0.156 | 4.7 × 10^–2^ | 0 |
| CGC | –0.022 | –0.118 | 0.073 | 0.65 | 27.4 |
| CGH | –0.058 | –0.176 | 0.061 | 0.34 | 51.3 |
| CR | 0.021 | –0.111 | 0.152 | 0.76 | 60.4 |
| CST | –0.075 | –0.166 | 0.018 | 0.12 | 23.6 |
| EC | –0.098 | –0.276 | 0.087 | 0.30 | 80.1 |
| FX | 0.299 | 0.227 | 0.369 | **9.5 × 10^–15^** | 0 |
| FXST | –0.131 | –0.249 | –0.008 | 3.7 × 10^–2^ | 54.4 |
| GCC | 0.045 | –0.035 | 0.123 | 0.27 | 1.8 |
| IC | –0.129 | –0.263 | 0.009 | 6.8 × 10^–2^ | 64.3 |
| IFO | –0.093 | –0.188 | 0.004 | 6.1 × 10^–2^ | 29.2 |
| PCR | 0.043 | –0.089 | 0.174 | 0.52 | 60.4 |
| PLIC | –0.076 | –0.191 | 0.041 | 0.20 | 49.4 |
| PTR | –0.015 | –0.103 | 0.073 | 0.74 | 16.3 |
| RLIC | –0.101 | –0.243 | 0.045 | 0.18 | 67.7 |
| SCC | 0.076 | –0.032 | 0.181 | 0.17 | 40.5 |
| SCR | 0.057 | –0.055 | 0.168 | 0.32 | 45.1 |
| SFO | –0.073 | –0.189 | 0.044 | 0.22 | 49.9 |
| SLF | 0.020 | –0.094 | 0.133 | 0.73 | 46.8 |
| SS | 0.036 | –0.060 | 0.131 | 0.46 | 27.3 |
| UNC | 0.219 | 0.109 | 0.324 | **1.2 × 10^–4^** | 46.2 |

**Supplementary Table 61** Mega-analysis results of correlation between radial diffusivity (RD) and duration of illness in patients with schizophrenia

| Region of interest | *β* | 95% CI-lower bound | 95% CI-upper bound | *p* | *I^2^* (%) |
| --- | --- | --- | --- | --- | --- |
| ACR | 0.294 | 0.182 | 0.398 | **6.2 × 10^–7^** | 51.0 |
| ALIC | 0.171 | 0.044 | 0.292 | 8.4 × 10^–3^ | 57.8 |
| Average RD | 0.227 | 0.100 | 0.347 | **5.5 × 10^–4^** | 59.4 |
| BCC | 0.262 | 0.182 | 0.339 | **4.6 × 10^–10^** | 10.9 |
| CC | 0.277 | 0.177 | 0.372 | **1.5 × 10^–7^** | 39.5 |
| CGC | 0.140 | 0.022 | 0.253 | 2.0 × 10^–2^ | 50.4 |
| CGH | –0.088 | –0.210 | 0.037 | 0.17 | 55.6 |
| CR | 0.288 | 0.176 | 0.394 | **1.1 × 10^–6^** | 51.5 |
| CST | –0.034 | –0.119 | 0.052 | 0.44 | 12.7 |
| EC | 0.150 | 0.015 | 0.280 | 3.0 × 10^–2^ | 62.7 |
| FX | 0.332 | 0.260 | 0.399 | **5.3 × 10^–18^** | 0 |
| FXST | 0.119 | 0.036 | 0.200 | 5.0 × 10^–3^ | 9.0 |
| GCC | 0.248 | 0.129 | 0.360 | **5.9 × 10^–5^** | 54.5 |
| IC | 0.153 | 0.030 | 0.271 | 1.5 × 10^–2^ | 54.7 |
| IFO | 0.045 | –0.054 | 0.144 | 0.37 | 31.7 |
| PCR | 0.274 | 0.175 | 0.369 | **1.6 × 10^–7^** | 38.4 |
| PLIC | 0.071 | –0.007 | 0.148 | 7.4 × 10^–2^ | 0 |
| PTR | 0.290 | 0.168 | 0.402 | **5.2 × 10^–6^** | 57.7 |
| RLIC | 0.166 | 0.029 | 0.297 | 1.8 × 10^–2^ | 63.7 |
| SCC | 0.165 | 0.045 | 0.279 | 7.1 × 10^–3^ | 52.7 |
| SCR | 0.227 | 0.120 | 0.329 | **4.1 × 10^–5^** | 43.9 |
| SFO | 0.218 | 0.113 | 0.318 | **5.8 × 10^–5^** | 41.0 |
| SLF | 0.217 | 0.103 | 0.325 | **2.3 × 10^–4^** | 49.8 |
| SS | 0.205 | 0.083 | 0.321 | **1.1 × 10^–3^** | 55.4 |
| UNC | 0.236 | 0.101 | 0.362 | **7.0 × 10^–4^** | 63.9 |

**Supplementary Table 62** Mega-analysis results of correlation between fractional anisotropy (FA) and duration of illness in patients with bipolar disorder

| Region of interest | *β* | 95% CI-lower bound | 95% CI-upper bound | *p* | *I^2^* (%) |
| --- | --- | --- | --- | --- | --- |
| ACR | –0.287 | –0.417 | –0.147 | **9.1 × 10^–5^** | 0 |
| ALIC | –0.193 | –0.343 | –0.032 | 1.9 × 10^–2^ | 12.7 |
| Average FA | –0.258 | –0.396 | –0.108 | **9.0 × 10^–4^** | 6.7 |
| BCC | –0.236 | –0.421 | –0.033 | 2.3 × 10^–2^ | 42.9 |
| CC | –0.237 | –0.396 | –0.064 | 7.5 × 10^–3^ | 23.9 |
| CGC | –0.239 | –0.415 | –0.045 | 1.6 × 10^–2^ | 37.7 |
| CGH | 0.083 | –0.065 | 0.227 | 0.27 | 0 |
| CR | –0.218 | –0.353 | –0.073 | 3.5 × 10^–3^ | 0 |
| CST | 0.002 | –0.170 | 0.173 | 0.99 | 20.8 |
| EC | –0.304 | –0.509 | –0.066 | 1.3 × 10^–2^ | 59.3 |
| FX | –0.240 | –0.374 | –0.097 | **1.2 × 10^–3^** | 0 |
| FXST | –0.158 | –0.298 | –0.011 | 3.5 × 10^–2^ | 0 |
| GCC | –0.263 | –0.394 | –0.120 | **3.8 × 10^–4^** | 0 |
| IC | –0.155 | –0.296 | –0.008 | 3.8 × 10^–2^ | 0 |
| IFO | –0.093 | –0.237 | 0.055 | 0.22 | 0 |
| PCR | –0.081 | –0.225 | 0.067 | 0.29 | 0 |
| PLIC | –0.118 | –0.260 | 0.030 | 0.12 | 0 |
| PTR | –0.201 | –0.339 | –0.056 | 6.9 × 10^–3^ | 0 |
| RLIC | –0.077 | –0.221 | 0.071 | 0.31 | 0 |
| SCC | –0.097 | –0.241 | 0.050 | 0.20 | 0 |
| SCR | –0.115 | –0.292 | 0.070 | 0.22 | 29.7 |
| SFO | –0.253 | –0.443 | –0.042 | 1.9 × 10^–2^ | 47.2 |
| SLF | –0.186 | –0.324 | –0.040 | 1.3 × 10^–2^ | 0 |
| SS | –0.175 | –0.335 | –0.006 | 4.2 × 10^–2^ | 19.4 |
| UNC | –0.084 | –0.238 | 0.074 | 0.30 | 9 |

**Supplementary Table 63** Mega-analysis results of correlation between mean diffusivity (MD) and duration of illness in patients with bipolar disorder

| Region of interest | *β* | 95% CI-lower bound | 95% CI-upper bound | *p* | *I^2^* (%) |
| --- | --- | --- | --- | --- | --- |
| ACR | 0.174 | 0.028 | 0.313 | 2.0 × 10^–2^ | 0 |
| ALIC | 0.265 | 0.017 | 0.482 | 3.7 × 10^–2^ | 61.4 |
| Average MD | 0.222 | 0.077 | 0.357 | 2.9 × 10^–3^ | 0 |
| BCC | 0.186 | –0.025 | 0.382 | 8.4 × 10^–2^ | 45.8 |
| CC | 0.177 | 0.031 | 0.316 | 1.8 × 10^–2^ | 0.2 |
| CGC | 0.096 | –0.052 | 0.240 | 0.20 | 0 |
| CGH | –0.086 | –0.270 | 0.103 | 0.37 | 32.7 |
| CR | 0.170 | 0.023 | 0.309 | 2.3 × 10^–2^ | 0 |
| CST | –0.026 | –0.218 | 0.168 | 0.79 | 36.0 |
| EC | 0.311 | 0.084 | 0.508 | 7.9 × 10^–3^ | 55.8 |
| FX | 0.266 | 0.123 | 0.398 | **3.4 × 10^–4^** | 0.8 |
| FXST | 0.218 | 0.073 | 0.353 | 3.4 × 10^–3^ | 0 |
| GCC | 0.210 | 0.065 | 0.346 | 4.9 × 10^–3^ | 0 |
| IC | 0.224 | 0.080 | 0.360 | 2.5 × 10^–3^ | 0 |
| IFO | –0.035 | –0.317 | 0.253 | 0.82 | 71.0 |
| PCR | 0.135 | –0.012 | 0.277 | 7.2 × 10^–2^ | 0 |
| PLIC | 0.195 | 0.049 | 0.333 | 9.0 × 10^–3^ | 0 |
| PTR | 0.121 | –0.027 | 0.263 | 0.11 | 0 |
| RLIC | 0.156 | 0.009 | 0.297 | 3.7 × 10^–2^ | 0 |
| SCC | 0.054 | –0.094 | 0.200 | 0.47 | 0 |
| SCR | 0.172 | –0.002 | 0.337 | 5.3 × 10^–2^ | 23.4 |
| SFO | 0.216 | –0.069 | 0.468 | 0.14 | 70.0 |
| SLF | 0.108 | –0.040 | 0.251 | 0.15 | 0 |
| SS | 0.182 | 0.036 | 0.321 | 1.5 × 10^–2^ | 0 |
| UNC | 0.125 | –0.022 | 0.268 | 0.10 | 0 |

**Supplementary Table 64** Mega-analysis results of correlation between axial diffusivity (AD) and duration of illness in patients with bipolar disorder

| Region of interest | *β* | 95% CI-lower bound | 95% CI-upper bound | *p* | *I^2^* (%) |
| --- | --- | --- | --- | --- | --- |
| ACR | 0.064 | –0.105 | 0.230 | 0.46 | 18.6 |
| ALIC | 0.164 | 0.015 | 0.306 | 3.1 × 10^–2^ | 1.9 |
| Average AD | 0.127 | –0.020 | 0.269 | 9.1 × 10^–2^ | 0 |
| BCC | 0.055 | –0.093 | 0.201 | 0.46 | 0 |
| CC | 0.035 | –0.113 | 0.181 | 0.65 | 0 |
| CGC | –0.010 | –0.157 | 0.137 | 0.89 | 0 |
| CGH | –0.106 | –0.286 | 0.083 | 0.27 | 31.9 |
| CR | 0.128 | –0.020 | 0.270 | 8.9 × 10^–2^ | 0 |
| CST | –0.030 | –0.242 | 0.185 | 0.79 | 47.5 |
| EC | 0.190 | 0.044 | 0.328 | 1.1 × 10^–2^ | 0 |
| FX | 0.239 | 0.095 | 0.374 | **1.3 × 10^–3^** | 1.0 |
| FXST | 0.042 | –0.245 | 0.323 | 0.78 | 70.9 |
| GCC | 0.027 | –0.120 | 0.174 | 0.72 | 0 |
| IC | 0.163 | 0.016 | 0.302 | 3.0 × 10^–2^ | 0 |
| IFO | –0.148 | –0.358 | 0.077 | 0.20 | 51.1 |
| PCR | 0.129 | –0.018 | 0.271 | 8.5 × 10^–2^ | 0 |
| PLIC | 0.170 | 0.024 | 0.309 | 2.3 × 10^–2^ | 0 |
| PTR | –0.042 | –0.188 | 0.105 | 0.58 | 0 |
| RLIC | 0.052 | –0.140 | 0.240 | 0.60 | 34.6 |
| SCC | –0.018 | –0.165 | 0.129 | 0.81 | 0 |
| SCR | 0.168 | 0.021 | 0.307 | 2.5 × 10^–2^ | 0 |
| SFO | 0.110 | –0.115 | 0.325 | 0.34 | 51.4 |
| SLF | 0.010 | –0.137 | 0.157 | 0.89 | 0 |
| SS | 0.077 | –0.071 | 0.221 | 0.31 | 0 |
| UNC | 0.116 | –0.032 | 0.258 | 0.12 | 0 |

**Supplementary Table 65** Mega-analysis results of correlation between radial diffusivity (RD) and duration of illness in patients with bipolar disorder

| Region of interest | *β* | 95% CI-lower bound | 95% CI-upper bound | *p* | *I^2^* (%) |
| --- | --- | --- | --- | --- | --- |
| ACR | 0.221 | 0.076 | 0.356 | 3.0 × 10^–3^ | 0 |
| ALIC | 0.271 | 0.012 | 0.496 | 4.1 × 10^–2^ | 64.7 |
| Average RD | 0.253 | 0.110 | 0.386 | **6.1 × 10^–4^** | 0 |
| BCC | 0.217 | –0.002 | 0.417 | 5.2 × 10^–2^ | 50.0 |
| CC | 0.223 | 0.034 | 0.397 | 2.1 × 10^–2^ | 34.5 |
| CGC | 0.158 | –0.036 | 0.340 | 0.11 | 35.7 |
| CGH | –0.070 | –0.215 | 0.078 | 0.35 | 0 |
| CR | 0.179 | 0.033 | 0.318 | 1.7 × 10^–2^ | 0 |
| CST | 0.015 | –0.160 | 0.189 | 0.87 | 23.1 |
| EC | 0.359 | 0.080 | 0.585 | 1.3 × 10^–2^ | 71.4 |
| FX | 0.275 | 0.133 | 0.406 | **1.9 × 10^–4^** | 0 |
| FXST | 0.262 | 0.120 | 0.394 | **3.9 × 10^–4^** | 0 |
| GCC | 0.265 | 0.113 | 0.405 | **7.6 × 10^–4^** | 9.0 |
| IC | 0.234 | 0.090 | 0.368 | **1.6 × 10^–3^** | 0 |
| IFO | 0.062 | –0.197 | 0.314 | 0.64 | 63.7 |
| PCR | 0.122 | –0.026 | 0.264 | 0.10 | 0 |
| PLIC | 0.190 | 0.044 | 0.327 | 1.1 × 10^–2^ | 0 |
| PTR | 0.189 | 0.043 | 0.327 | 1.2 × 10^–2^ | 0 |
| RLIC | 0.187 | 0.041 | 0.325 | 1.2 × 10^–2^ | 0 |
| SCC | 0.108 | –0.040 | 0.251 | 0.15 | 0 |
| SCR | 0.149 | –0.042 | 0.330 | 0.13 | 34.1 |
| SFO | 0.249 | –0.038 | 0.498 | 8.8 × 10^–2^ | 70.8 |
| SLF | 0.145 | –0.020 | 0.302 | 8.5 × 10^–2^ | 15.3 |
| SS | 0.217 | 0.072 | 0.353 | 3.5 × 10^–3^ | 0 |
| UNC | 0.123 | –0.088 | 0.323 | 0.25 | 44.7 |

**Supplementary Table 66** Mega-analysis results of correlation between fractional anisotropy (FA) and duration of illness (age) in individuals with autism spectrum disorder

| Region of interest | *β* | 95% CI-lower bound | 95% CI-upper bound | *p* | *I^2^* (%) |
| --- | --- | --- | --- | --- | --- |
| ACR | –0.276 | –0.450 | –0.082 | 5.8 × 10^–3^ | 0 |
| ALIC | –0.065 | –0.292 | 0.169 | 0.59 | 18.3 |
| Average FA | –0.221 | –0.402 | –0.024 | 2.9 × 10^–2^ | 0 |
| BCC | –0.097 | –0.290 | 0.104 | 0.34 | 0 |
| CC | –0.226 | –0.406 | –0.029 | 2.5 × 10^–2^ | 0 |
| CGC | –0.008 | –0.206 | 0.191 | 0.94 | 0 |
| CGH | –0.029 | –0.292 | 0.238 | 0.83 | 33.9 |
| CR | –0.357 | –0.518 | –0.170 | **2.8 × 10^–4^** | 0 |
| CST | –0.017 | –0.214 | 0.183 | 0.87 | 0 |
| EC | –0.075 | –0.443 | 0.315 | 0.71 | 66.7 |
| FX | –0.077 | –0.271 | 0.124 | 0.46 | 0 |
| FXST | –0.202 | –0.385 | –0.004 | 4.6 × 10^–2^ | 0 |
| GCC | –0.380 | –0.549 | –0.182 | **2.9 × 10^–4^** | 8.4 |
| IC | –0.315 | –0.483 | –0.125 | **1.5 × 10^–3^** | 0 |
| IFO | 0.119 | –0.307 | 0.505 | 0.59 | 71.7 |
| PCR | –0.338 | –0.502 | –0.149 | **6.2 × 10^–4^** | 0 |
| PLIC | –0.292 | –0.464 | –0.100 | 3.3 × 10^–3^ | 0 |
| PTR | –0.397 | –0.552 | –0.216 | **4.2 × 10^–5^** | 0 |
| RLIC | –0.361 | –0.522 | –0.175 | **2.3 × 10^–4^** | 0 |
| SCC | –0.242 | –0.420 | –0.045 | 1.6 × 10^–2^ | 0 |
| SCR | –0.307 | –0.476 | –0.115 | 2.0 × 10^–3^ | 0 |
| SFO | –0.208 | –0.391 | –0.010 | 3.9 × 10^–2^ | 0 |
| SLF | –0.270 | –0.444 | –0.075 | 7.0 × 10^–3^ | 0 |
| SS | –0.378 | –0.637 | –0.041 | 2.9 × 10^–2^ | 58.6 |
| UNC | 0.059 | –0.318 | 0.421 | 0.76 | 64.8 |

**Supplementary Table 67** Mega-analysis results of correlation between mean diffusivity (MD) and duration of illness (age) in individuals with autism spectrum disorder

| Region of interest | *β* | 95% CI-lower bound | 95% CI-upper bound | *p* | *I^2^* (%) |
| --- | --- | --- | --- | --- | --- |
| ACR | 0.194 | –0.005 | 0.378 | 5.5 × 10^–2^ | 0 |
| ALIC | –0.318 | –0.539 | –0.055 | 1.8 × 10^–2^ | 34.8 |
| Average MD | 0.164 | –0.366 | 0.614 | 0.55 | 81.9 |
| BCC | 0.096 | –0.105 | 0.289 | 0.35 | 0 |
| CC | 0.201 | 0.003 | 0.384 | 4.7 × 10^–2^ | 0 |
| CGC | 0.064 | –0.136 | 0.260 | 0.53 | 0 |
| CGH | –0.081 | –0.275 | 0.119 | 0.43 | 0 |
| CR | 0.217 | –0.063 | 0.465 | 0.13 | 38.5 |
| CST | –0.169 | –0.496 | 0.200 | 0.37 | 62.0 |
| EC | –0.102 | –0.294 | 0.099 | 0.32 | 0 |
| FX | 0.127 | –0.209 | 0.435 | 0.46 | 54.7 |
| FXST | –0.220 | –0.538 | 0.154 | 0.25 | 62.9 |
| GCC | 0.246 | 0.050 | 0.424 | 1.4 × 10^–2^ | 0 |
| IC | –0.226 | –0.406 | –0.029 | 2.5 × 10^–2^ | 0 |
| IFO | –0.112 | –0.304 | 0.088 | 0.27 | 0 |
| PCR | 0.327 | –0.116 | 0.662 | 0.14 | 74.0 |
| PLIC | –0.354 | –0.516 | –0.167 | **3.1 × 10^–4^** | 0 |
| PTR | 0.421 | 0.006 | 0.712 | 4.7 × 10^–2^ | 72.5 |
| RLIC | 0.019 | –0.181 | 0.216 | 0.86 | 0 |
| SCC | 0.314 | –0.080 | 0.623 | 0.12 | 67.4 |
| SCR | 0.081 | –0.119 | 0.275 | 0.43 | 0 |
| SFO | –0.030 | –0.240 | 0.182 | 0.78 | 7.3 |
| SLF | 0.216 | –0.057 | 0.460 | 0.12 | 36.1 |
| SS | 0.218 | –0.296 | 0.634 | 0.41 | 80.3 |
| UNC | 0.415 | 0.118 | 0.644 | 7.4 × 10^–3^ | 50.6 |

**Supplementary Table 68** Mega-analysis results of correlation between axial diffusivity (AD) and duration of illness (age) in individuals with autism spectrum disorder

| Region of interest | *β* | 95% CI-lower bound | 95% CI-upper bound | *p* | *I^2^* (%) |
| --- | --- | --- | --- | --- | --- |
| ACR | –0.012 | –0.210 | 0.187 | 0.91 | 0 |
| ALIC | –0.388 | –0.545 | –0.205 | **6.6 × 10^–5^** | 0 |
| Average AD | –0.034 | –0.585 | 0.538 | 0.92 | 86.4 |
| BCC | –0.003 | –0.201 | 0.196 | 0.98 | 0 |
| CC | –0.024 | –0.221 | 0.176 | 0.82 | 0 |
| CGC | 0.014 | –0.185 | 0.212 | 0.89 | 0 |
| CGH | –0.040 | –0.455 | 0.388 | 0.86 | 73.3 |
| CR | –0.082 | –0.309 | 0.154 | 0.50 | 19.2 |
| CST | –0.231 | –0.480 | 0.053 | 0.11 | 39.9 |
| EC | –0.130 | –0.476 | 0.250 | 0.51 | 64.4 |
| FX | 0.076 | –0.230 | 0.368 | 0.63 | 47.1 |
| FXST | –0.357 | –0.563 | –0.111 | 5.3 × 10^–3^ | 30.5 |
| GCC | –0.081 | –0.275 | 0.119 | 0.43 | 0 |
| IC | –0.520 | –0.651 | –0.359 | **1.9 × 10^–8^** | 0 |
| IFO | 0.022 | –0.378 | 0.416 | 0.92 | 69.6 |
| PCR | 0.062 | –0.409 | 0.507 | 0.81 | 78.0 |
| PLIC | –0.550 | –0.682 | –0.384 | **1.5 × 10^–8^** | 7.2 |
| PTR | 0.104 | –0.250 | 0.433 | 0.57 | 59.3 |
| RLIC | –0.280 | –0.453 | –0.086 | 5.1 × 10^–3^ | 0 |
| SCC | 0.046 | –0.208 | 0.294 | 0.73 | 28 |
| SCR | –0.199 | –0.382 | 0 | 5.0 × 10^–2^ | 0 |
| SFO | –0.145 | –0.334 | 0.055 | 0.15 | 0 |
| SLF | –0.020 | –0.308 | 0.272 | 0.90 | 43.3 |
| SS | –0.111 | –0.348 | 0.139 | 0.38 | 25.8 |
| UNC | 0.473 | –0.209 | 0.845 | 0.17 | 89.6 |

**Supplementary Table 69** Mega-analysis results of correlation between radial diffusivity (RD) and duration of illness (age) in individuals with autism spectrum disorder

| Region of interest | *β* | 95% CI-lower bound | 95% CI-upper bound | *p* | *I^2^* (%) |
| --- | --- | --- | --- | --- | --- |
| ACR | 0.261 | 0.066 | 0.436 | 9.3 × 10^–3^ | 0 |
| ALIC | –0.071 | –0.277 | 0.142 | 0.52 | 7.1 |
| Average RD | 0.216 | –0.160 | 0.537 | 0.26 | 63.2 |
| BCC | 0.105 | –0.096 | 0.297 | 0.30 | 0 |
| CC | 0.236 | 0.040 | 0.415 | 1.9 × 10^–2^ | 0 |
| CGC | 0.047 | –0.153 | 0.243 | 0.64 | 0 |
| CGH | –0.027 | –0.224 | 0.173 | 0.79 | 0 |
| CR | 0.308 | 0.116 | 0.477 | **1.9 × 10^–3^** | 0 |
| CST | –0.064 | –0.419 | 0.307 | 0.74 | 63.5 |
| EC | 0.025 | –0.174 | 0.222 | 0.81 | 0 |
| FX | 0.151 | –0.197 | 0.466 | 0.40 | 57.7 |
| FXST | 0.040 | –0.248 | 0.321 | 0.79 | 41.6 |
| GCC | 0.413 | 0.131 | 0.632 | 5.0 × 10^–3^ | 46.0 |
| IC | 0.119 | –0.081 | 0.310 | 0.24 | 0 |
| IFO | –0.140 | –0.404 | 0.144 | 0.33 | 39.5 |
| PCR | 0.335 | 0.144 | 0.502 | **7.9 × 10^–4^** | 1.2 |
| PLIC | 0.087 | –0.113 | 0.281 | 0.39 | 0 |
| PTR | 0.411 | 0.215 | 0.574 | **8.6 × 10^–5^** | 9.2 |
| RLIC | 0.232 | 0.035 | 0.412 | 2.1 × 10^–2^ | 0 |
| SCC | 0.274 | 0.062 | 0.463 | 1.2 × 10^–2^ | 10.3 |
| SCR | 0.273 | 0.079 | 0.447 | 6.3 × 10^–3^ | 0 |
| SFO | 0.082 | –0.118 | 0.276 | 0.42 | 0 |
| SLF | 0.277 | 0.083 | 0.451 | 5.6 × 10^–3^ | 0 |
| SS | 0.302 | –0.157 | 0.654 | 0.19 | 75.5 |
| UNC | 0.174 | –0.025 | 0.360 | 8.7 × 10^–2^ | 0 |

**Supplementary Table 70** Mega-analysis results of correlation between fractional anisotropy (FA) and duration of illness in patients with major depressive disorder

| Region of interest | *β* | 95% CI-lower bound | 95% CI-upper bound | *p* | *I^2^* (%) |
| --- | --- | --- | --- | --- | --- |
| ACR | –0.181 | –0.327 | –0.026 | 2.2 × 10^–2^ | 45.7 |
| ALIC | –0.127 | –0.258 | 0.009 | 6.6 × 10^–2^ | 30.8 |
| Average FA | –0.194 | –0.293 | –0.091 | **2.5 × 10^–4^** | 0.2 |
| BCC | –0.192 | –0.353 | –0.020 | 2.9 × 10^–2^ | 55.8 |
| CC | –0.199 | –0.390 | 0.009 | 6.0 × 10^–2^ | 69.6 |
| CGC | –0.158 | –0.287 | –0.025 | 2.1 × 10^–2^ | 30.0 |
| CGH | –0.075 | –0.178 | 0.030 | 0.16 | 0 |
| CR | –0.138 | –0.263 | –0.009 | 3.7 × 10^–2^ | 25.8 |
| CST | –0.028 | –0.132 | 0.077 | 0.60 | 0 |
| EC | –0.173 | –0.291 | –0.049 | 6.5 × 10^–3^ | 21.3 |
| FX | –0.227 | –0.324 | –0.126 | **1.6 × 10^–5^** | 0 |
| FXST | –0.180 | –0.280 | –0.077 | **6.8 × 10^–4^** | 0 |
| GCC | –0.204 | –0.402 | 0.013 | 6.5 × 10^–2^ | 72.1 |
| IC | –0.091 | –0.194 | 0.014 | 9.0 × 10^–2^ | 0 |
| IFO | –0.097 | –0.199 | 0.008 | 7.1 × 10^–2^ | 0 |
| PCR | –0.076 | –0.179 | 0.030 | 0.16 | 0 |
| PLIC | –0.045 | –0.150 | 0.060 | 0.40 | 0 |
| PTR | –0.112 | –0.214 | –0.007 | 3.7 × 10^–2^ | 0 |
| RLIC | –0.073 | –0.209 | 0.066 | 0.30 | 33.3 |
| SCC | –0.115 | –0.270 | 0.046 | 0.16 | 48.9 |
| SCR | –0.050 | –0.186 | 0.088 | 0.48 | 32.6 |
| SFO | –0.209 | –0.307 | –0.107 | **7.7 × 10^–5^** | 0 |
| SLF | –0.054 | –0.158 | 0.051 | 0.31 | 0 |
| SS | –0.147 | –0.248 | –0.042 | 6.0 × 10^–3^ | 0 |
| UNC | –0.074 | –0.211 | 0.065 | 0.29 | 33.6 |

**Supplementary Table 71** Mega-analysis results of correlation between mean diffusivity (MD) and duration of illness in patients with major depressive disorder

| Region of interest | *β* | 95% CI-lower bound | 95% CI-upper bound | *p* | *I^2^* (%) |
| --- | --- | --- | --- | --- | --- |
| ACR | 0.035 | –0.184 | 0.250 | 0.76 | 72.5 |
| ALIC | 0.115 | –0.045 | 0.268 | 0.16 | 47.9 |
| Average MD | 0.136 | –0.061 | 0.323 | 0.18 | 65.8 |
| BCC | 0.193 | 0.024 | 0.352 | 2.5 × 10^–2^ | 54.5 |
| CC | 0.174 | –0.015 | 0.352 | 7.1 × 10^–2^ | 63.3 |
| CGC | 0.102 | –0.039 | 0.239 | 0.16 | 35.2 |
| CGH | 0.067 | –0.048 | 0.181 | 0.25 | 11.1 |
| CR | 0.103 | –0.062 | 0.264 | 0.22 | 51.6 |
| CST | 0.036 | –0.095 | 0.166 | 0.59 | 26.5 |
| EC | 0.138 | –0.092 | 0.354 | 0.24 | 75.0 |
| FX | 0.226 | 0.119 | 0.327 | **4.1 × 10^–5^** | 4.8 |
| FXST | 0.053 | –0.191 | 0.290 | 0.68 | 78.1 |
| GCC | 0.113 | –0.073 | 0.291 | 0.23 | 61.3 |
| IC | 0.120 | –0.035 | 0.270 | 0.13 | 45.2 |
| IFO | 0.037 | –0.088 | 0.162 | 0.56 | 21.6 |
| PCR | 0.049 | –0.133 | 0.228 | 0.60 | 59.8 |
| PLIC | 0.136 | 0.032 | 0.237 | 1.1 × 10^–2^ | 0 |
| PTR | –0.004 | –0.230 | 0.223 | 0.97 | 74.9 |
| RLIC | 0.020 | –0.215 | 0.254 | 0.87 | 76.6 |
| SCC | 0.113 | –0.079 | 0.297 | 0.25 | 63.8 |
| SCR | 0.168 | 0.064 | 0.268 | **1.6 × 10^–3^** | 0 |
| SFO | 0.088 | –0.069 | 0.241 | 0.27 | 46.2 |
| SLF | 0.089 | –0.072 | 0.246 | 0.28 | 48.9 |
| SS | 0.047 | –0.192 | 0.281 | 0.70 | 77.1 |
| UNC | 0.063 | –0.111 | 0.234 | 0.48 | 56.3 |

**Supplementary Table 72** Mega-analysis results of correlation between axial diffusivity (AD) and duration of illness in patients with major depressive disorder

| Region of interest | *β* | 95% CI-lower bound | 95% CI-upper bound | *p* | *I^2^* (%) |
| --- | --- | --- | --- | --- | --- |
| ACR | –0.039 | –0.224 | 0.149 | 0.69 | 62.4 |
| ALIC | 0.090 | –0.032 | 0.208 | 0.15 | 17.4 |
| Average AD | 0.042 | –0.169 | 0.249 | 0.70 | 70.2 |
| BCC | 0.084 | –0.028 | 0.194 | 0.14 | 7.9 |
| CC | 0.058 | –0.084 | 0.197 | 0.43 | 35.4 |
| CGC | 0.013 | –0.095 | 0.120 | 0.82 | 3.1 |
| CGH | 0.003 | –0.116 | 0.122 | 0.96 | 15.6 |
| CR | 0.075 | –0.056 | 0.203 | 0.26 | 26.3 |
| CST | –0.020 | –0.133 | 0.094 | 0.73 | 9.1 |
| EC | 0.023 | –0.220 | 0.263 | 0.86 | 78.0 |
| FX | 0.208 | 0.102 | 0.310 | **1.4 × 10^–4^** | 4.0 |
| FXST | –0.086 | –0.327 | 0.165 | 0.50 | 79.2 |
| GCC | <0.001 | –0.160 | 0.160 | 1.00 | 48.9 |
| IC | 0.083 | –0.072 | 0.234 | 0.29 | 45.0 |
| IFO | –0.044 | –0.193 | 0.106 | 0.56 | 42.3 |
| PCR | 0.029 | –0.125 | 0.182 | 0.71 | 44.7 |
| PLIC | 0.136 | 0.031 | 0.237 | 1.1 × 10^–2^ | 0 |
| PTR | –0.043 | –0.233 | 0.151 | 0.67 | 64.3 |
| RLIC | –0.045 | –0.279 | 0.194 | 0.71 | 77.1 |
| SCC | 0.074 | –0.055 | 0.201 | 0.26 | 25.1 |
| SCR | 0.161 | 0.057 | 0.261 | 2.6 × 10^–3^ | 0 |
| SFO | 0.060 | –0.069 | 0.187 | 0.36 | 24.8 |
| SLF | 0.066 | –0.068 | 0.198 | 0.34 | 29.3 |
| SS | –0.074 | –0.308 | 0.169 | 0.55 | 77.7 |
| UNC | 0.075 | –0.062 | 0.208 | 0.28 | 31.1 |

**Supplementary Table 73** Mega-analysis results of correlation between radial diffusivity (RD) and duration of illness in patients with major depressive disorder

| Region of interest | *β* | 95% CI-lower bound | 95% CI-upper bound | *p* | *I^2^* (%) |
| --- | --- | --- | --- | --- | --- |
| ACR | 0.086 | –0.135 | 0.299 | 0.45 | 72.8 |
| ALIC | 0.137 | –0.032 | 0.298 | 0.11 | 53.5 |
| Average RD | 0.174 | –0.010 | 0.346 | 6.4 × 10^–2^ | 60.8 |
| BCC | 0.223 | 0.045 | 0.387 | 1.4 × 10^–2^ | 59.2 |
| CC | 0.222 | 0.018 | 0.409 | 3.3 × 10^–2^ | 69.1 |
| CGC | 0.156 | –0.003 | 0.308 | 5.5 × 10^–2^ | 48.3 |
| CGH | 0.122 | 0.017 | 0.223 | 2.3 × 10^–2^ | 0 |
| CR | 0.114 | –0.061 | 0.282 | 0.20 | 56.3 |
| CST | 0.067 | –0.069 | 0.201 | 0.34 | 31.4 |
| EC | 0.178 | –0.032 | 0.372 | 9.6 × 10^–2^ | 70.0 |
| FX | 0.229 | 0.119 | 0.334 | **5.9 × 10^–5^** | 9.4 |
| FXST | 0.166 | –0.006 | 0.329 | 5.9 × 10^–2^ | 55.6 |
| GCC | 0.188 | –0.025 | 0.383 | 8.3 × 10^–2^ | 70.8 |
| IC | 0.138 | <0.001 | 0.271 | 4.9 × 10^–2^ | 33.2 |
| IFO | 0.060 | –0.068 | 0.185 | 0.36 | 23.5 |
| PCR | 0.059 | –0.118 | 0.232 | 0.51 | 57.3 |
| PLIC | 0.095 | –0.010 | 0.198 | 7.5 × 10^–2^ | 0 |
| PTR | 0.074 | –0.099 | 0.244 | 0.40 | 55.8 |
| RLIC | 0.090 | –0.091 | 0.265 | 0.33 | 59.1 |
| SCC | 0.143 | –0.026 | 0.305 | 9.8 × 10^–2^ | 54.0 |
| SCR | 0.098 | –0.059 | 0.250 | 0.22 | 46.2 |
| SFO | 0.124 | –0.018 | 0.262 | 8.8 × 10^–2^ | 36.6 |
| SLF | 0.125 | –0.002 | 0.248 | 5.4 × 10^–2^ | 23.2 |
| SS | 0.158 | –0.003 | 0.311 | 5.4 × 10^–2^ | 49.4 |
| UNC | 0.050 | –0.131 | 0.228 | 0.59 | 59.5 |

**Legends of Supplementary Table 58–73**: Bold means statistical significant [*p* < 0.002 (0.05/25)].

Abbreviations: ACR, anterior corona radiata; AD, axial diffusivity; ALIC, anterior limb of internal capsule; BCC, body of corpus callosum; CC, corpus callosum; CGC, cingulum (cingulate gyrus); CGH, cingulum (hippocampus); CR, corona radiata; CST, corticospinal tract; EC, external capsule; FA, fractional anisotropy; FX, fornix; FX/ST, fornix (crus)/stria terminalis; GCC, genu of corpus callosum; IC, internal capsule; IFO, inferior fronto-occipital fasciculus; MD, mean diffusivity; PCR, posterior corona radiata; PLIC, posterior limb of internal capsule; PTR, posterior thalamic radiation; RD, radial diffusivity; RLIC, retrolenticular part of internal capsule; SCC, splenium of corpus callosum; SCR, superior corona radiata; SFO, superior fronto-occipital fasciculus; SLF, superior longitudinal fasciculus; SS, sagittal stratum; UNC, uncinate fasciculus.

**Supplementary Table 74** Mega-analysis results of correlation between fractional anisotropy (FA) and chlorpromazine equivalent dose in patients with schizophrenia

| Region of interest | *β* | 95% CI-lower bound | 95% CI-upper bound | *p* | *I^2^* (%) |
| --- | --- | --- | --- | --- | --- |
| ACR | –0.059 | –0.137 | 0.020 | 0.14 | 0 |
| ALIC | –0.078 | –0.155 | 0.001 | 5.2 × 10^–2^ | 0 |
| Average FA | –0.042 | –0.119 | 0.037 | 0.30 | 0 |
| BCC | –0.061 | –0.141 | 0.020 | 0.14 | 4.9 |
| CC | –0.045 | –0.127 | 0.039 | 0.29 | 8.7 |
| CGC | –0.054 | –0.132 | 0.024 | 0.18 | 0 |
| CGH | 0.034 | –0.049 | 0.116 | 0.43 | 8.5 |
| CR | –0.046 | –0.124 | 0.032 | 0.25 | 0 |
| CST | –0.016 | –0.106 | 0.074 | 0.72 | 19.8 |
| EC | –0.063 | –0.141 | 0.015 | 0.11 | 0 |
| FX | –0.003 | –0.081 | 0.075 | 0.94 | 0 |
| FXST | –0.142 | –0.317 | 0.041 | 0.13 | 80.2 |
| GCC | –0.049 | –0.129 | 0.033 | 0.24 | 5.6 |
| IC | –0.016 | –0.094 | 0.062 | 0.69 | 0 |
| IFO | –0.022 | –0.117 | 0.074 | 0.66 | 27.4 |
| PCR | –0.031 | –0.111 | 0.050 | 0.45 | 4.0 |
| PLIC | 0.015 | –0.063 | 0.093 | 0.71 | 0 |
| PTR | –0.051 | –0.141 | 0.039 | 0.26 | 19.6 |
| RLIC | 0.020 | –0.058 | 0.098 | 0.62 | 0 |
| SCC | 0.001 | –0.078 | 0.079 | 0.99 | 0 |
| SCR | –0.024 | –0.102 | 0.054 | 0.54 | 0 |
| SFO | –0.017 | –0.104 | 0.070 | 0.70 | 14.7 |
| SLF | –0.007 | –0.092 | 0.079 | 0.88 | 13.0 |
| SS | –0.014 | –0.092 | 0.064 | 0.73 | 0 |
| UNC | –0.021 | –0.100 | 0.058 | 0.61 | 1.7 |

**Supplementary Table 75** Mega-analysis results of correlation between mean diffusivity (MD) and chlorpromazine equivalent dose in patients with schizophrenia

| Region of interest | *β* | 95% CI-lower bound | 95% CI-upper bound | *p* | *I^2^* (%) |
| --- | --- | --- | --- | --- | --- |
| ACR | –0.002 | –0.080 | 0.077 | 0.97 | 0 |
| ALIC | 0.017 | –0.062 | 0.095 | 0.68 | 0 |
| Average MD | 0.041 | –0.038 | 0.119 | 0.31 | 0 |
| BCC | 0.064 | –0.033 | 0.160 | 0.20 | 29.7 |
| CC | 0.043 | –0.066 | 0.151 | 0.44 | 43.0 |
| CGC | –0.024 | –0.102 | 0.055 | 0.55 | 0 |
| CGH | –0.015 | –0.115 | 0.086 | 0.78 | 33.7 |
| CR | 0.001 | –0.077 | 0.079 | 0.98 | 0 |
| CST | 0.004 | –0.097 | 0.104 | 0.95 | 34.0 |
| EC | 0.017 | –0.062 | 0.095 | 0.68 | 0 |
| FX | 0.056 | –0.022 | 0.134 | 0.16 | 0 |
| FXST | 0.030 | –0.094 | 0.153 | 0.64 | 55.5 |
| GCC | 0.036 | –0.082 | 0.153 | 0.55 | 51.1 |
| IC | 0.018 | –0.061 | 0.096 | 0.66 | 0 |
| IFO | 0.007 | –0.122 | 0.137 | 0.91 | 59.3 |
| PCR | 0.009 | –0.084 | 0.101 | 0.85 | 23.5 |
| PLIC | 0.020 | –0.059 | 0.098 | 0.62 | 0 |
| PTR | –0.016 | –0.102 | 0.070 | 0.71 | 13.8 |
| RLIC | 0.007 | –0.071 | 0.085 | 0.86 | 0 |
| SCC | 0.006 | –0.088 | 0.100 | 0.89 | 25.4 |
| SCR | 0.009 | –0.070 | 0.087 | 0.83 | 0 |
| SFO | –0.031 | –0.117 | 0.055 | 0.48 | 13.7 |
| SLF | –0.016 | –0.094 | 0.063 | 0.70 | 0 |
| SS | 0.020 | –0.058 | 0.098 | 0.62 | 0 |
| UNC | 0.046 | –0.032 | 0.124 | 0.25 | 0 |

**Supplementary Table 76** Mega-analysis results of correlation between axial diffusivity (AD) and chlorpromazine equivalent dose in patients with schizophrenia

| Region of interest | *β* | 95% CI-lower bound | 95% CI-upper bound | *p* | *I^2^* (%) |
| --- | --- | --- | --- | --- | --- |
| ACR | –0.043 | –0.121 | 0.036 | 0.28 | 0 |
| ALIC | –0.026 | –0.104 | 0.053 | 0.52 | 0 |
| Average AD | 0.002 | –0.076 | 0.080 | 0.96 | 0 |
| BCC | 0.003 | –0.075 | 0.081 | 0.94 | 0 |
| CC | 0.008 | –0.080 | 0.096 | 0.85 | 17.1 |
| CGC | –0.073 | –0.151 | 0.005 | 6.6 × 10^–2^ | 0 |
| CGH | 0.029 | –0.050 | 0.108 | 0.47 | 0.8 |
| CR | –0.032 | –0.110 | 0.047 | 0.43 | 0 |
| CST | –0.018 | –0.096 | 0.061 | 0.66 | 0 |
| EC | –0.047 | –0.125 | 0.031 | 0.24 | 0 |
| FX | 0.063 | –0.015 | 0.141 | 0.12 | 0 |
| FXST | –0.037 | –0.157 | 0.084 | 0.55 | 53.3 |
| GCC | 0.001 | –0.113 | 0.116 | 0.98 | 48.3 |
| IC | 0.012 | –0.066 | 0.091 | 0.76 | 0 |
| IFO | –0.012 | –0.116 | 0.092 | 0.82 | 38.1 |
| PCR | –0.004 | –0.091 | 0.083 | 0.93 | 15.8 |
| PLIC | 0.038 | –0.041 | 0.115 | 0.35 | 0 |
| PTR | –0.069 | –0.149 | 0.012 | 0.10 | 4.8 |
| RLIC | 0.013 | –0.065 | 0.091 | 0.74 | 0 |
| SCC | 0.017 | –0.076 | 0.109 | 0.73 | 24.0 |
| SCR | –0.013 | –0.092 | 0.065 | 0.74 | 0 |
| SFO | –0.058 | –0.136 | 0.020 | 0.15 | 0 |
| SLF | –0.008 | –0.100 | 0.084 | 0.87 | 22.9 |
| SS | –0.004 | –0.082 | 0.075 | 0.93 | 0 |
| UNC | 0.066 | –0.051 | 0.181 | 0.27 | 49.9 |

**Supplementary Table 77** Mega-analysis results of correlation between radial diffusivity (RD) and chlorpromazine equivalent dose in patients with schizophrenia

| Region of interest | *β* | 95% CI-lower bound | 95% CI-upper bound | *p* | *I^2^* (%) |
| --- | --- | --- | --- | --- | --- |
| ACR | 0.021 | –0.057 | 0.099 | 0.60 | 0 |
| ALIC | 0.050 | –0.041 | 0.141 | 0.28 | 21.9 |
| Average RD | 0.049 | –0.029 | 0.127 | 0.22 | 0 |
| BCC | 0.063 | –0.034 | 0.159 | 0.20 | 29.5 |
| CC | 0.042 | –0.061 | 0.144 | 0.42 | 36.8 |
| CGC | 0.023 | –0.055 | 0.101 | 0.56 | 0 |
| CGH | –0.031 | –0.141 | 0.080 | 0.58 | 44.3 |
| CR | 0.014 | –0.064 | 0.093 | 0.72 | 0 |
| CST | 0.036 | –0.059 | 0.130 | 0.46 | 26.4 |
| EC | 0.047 | –0.032 | 0.125 | 0.24 | 0 |
| FX | 0.048 | –0.031 | 0.126 | 0.23 | 0 |
| FXST | 0.094 | –0.063 | 0.247 | 0.24 | 72.5 |
| GCC | 0.056 | –0.044 | 0.155 | 0.27 | 32.8 |
| IC | 0.016 | –0.063 | 0.094 | 0.69 | 0 |
| IFO | 0.005 | –0.102 | 0.113 | 0.92 | 41.9 |
| PCR | –0.008 | –0.086 | 0.070 | 0.84 | 0 |
| PLIC | –0.001 | –0.079 | 0.077 | 0.98 | 0 |
| PTR | 0.023 | –0.071 | 0.116 | 0.64 | 24.9 |
| RLIC | –0.002 | –0.081 | 0.076 | 0.95 | 0 |
| SCC | –0.001 | –0.084 | 0.082 | 0.99 | 8.5 |
| SCR | 0.014 | –0.064 | 0.092 | 0.73 | 0 |
| SFO | –0.008 | –0.098 | 0.082 | 0.86 | 19.7 |
| SLF | –0.017 | –0.095 | 0.061 | 0.67 | 0 |
| SS | 0.030 | –0.048 | 0.108 | 0.45 | 0 |
| UNC | 0.027 | –0.051 | 0.105 | 0.49 | 0 |

**Supplementary Table 78** Mega-analysis results of correlation between fractional anisotropy (FA) and lithium dose in patients with bipolar disorder

| Region of interest | *β* | 95% CI-lower bound | 95% CI-upper bound | *p* | *I^2^* (%) |
| --- | --- | --- | --- | --- | --- |
| ACR | 0.012 | –0.215 | 0.239 | 0.92 | 53.4 |
| ALIC | –0.049 | –0.244 | 0.150 | 0.63 | 38.8 |
| Average FA | –0.015 | –0.208 | 0.179 | 0.88 | 36.6 |
| BCC | –0.106 | –0.296 | 0.093 | 0.30 | 38.2 |
| CC | –0.044 | –0.190 | 0.103 | 0.56 | 0 |
| CGC | –0.037 | –0.182 | 0.111 | 0.63 | 0 |
| CGH | 0.004 | –0.143 | 0.150 | 0.96 | 0 |
| CR | –0.034 | –0.257 | 0.192 | 0.77 | 52.4 |
| CST | –0.171 | –0.391 | 0.068 | 0.16 | 57.0 |
| EC | –0.021 | –0.215 | 0.176 | 0.84 | 37.7 |
| FX | –0.061 | –0.260 | 0.144 | 0.56 | 41.8 |
| FXST | 0.052 | –0.095 | 0.197 | 0.49 | 0 |
| GCC | 0.018 | –0.129 | 0.164 | 0.81 | 0 |
| IC | –0.128 | –0.345 | 0.102 | 0.28 | 53.5 |
| IFO | –0.055 | –0.200 | 0.093 | 0.47 | 0 |
| PCR | 0.016 | –0.185 | 0.215 | 0.88 | 40.5 |
| PLIC | –0.160 | –0.407 | 0.109 | 0.24 | 66.1 |
| PTR | –0.062 | –0.207 | 0.086 | 0.41 | 0 |
| RLIC | –0.045 | –0.264 | 0.179 | 0.70 | 51.3 |
| SCC | –0.038 | –0.184 | 0.109 | 0.61 | 0 |
| SCR | –0.127 | –0.380 | 0.145 | 0.36 | 66.7 |
| SFO | –0.087 | –0.267 | 0.098 | 0.36 | 30.2 |
| SLF | –0.059 | –0.242 | 0.128 | 0.54 | 31.3 |
| SS | –0.005 | –0.201 | 0.191 | 0.96 | 38.2 |
| UNC | –0.078 | –0.222 | 0.069 | 0.30 | 0 |

**Supplementary Table 79** Mega-analysis results of correlation between mean diffusivity (MD) and lithium dose in patients with bipolar disorder

| Region of interest | *β* | 95% CI-lower bound | 95% CI-upper bound | *p* | *I^2^* (%) |
| --- | --- | --- | --- | --- | --- |
| ACR | 0.150 | –0.095 | 0.378 | 0.23 | 58.9 |
| ALIC | 0.009 | –0.155 | 0.173 | 0.91 | 15.5 |
| Average MD | 0.112 | –0.069 | 0.287 | 0.23 | 27.8 |
| BCC | 0.130 | –0.023 | 0.276 | 9.5 × 10^–2^ | 4.9 |
| CC | 0.101 | –0.046 | 0.244 | 0.18 | 0 |
| CGC | 0.069 | –0.079 | 0.214 | 0.36 | 0 |
| CGH | 0.111 | –0.166 | 0.371 | 0.43 | 67.8 |
| CR | 0.168 | –0.073 | 0.390 | 0.17 | 57.6 |
| CST | 0.061 | –0.111 | 0.230 | 0.49 | 21.3 |
| EC | –0.007 | –0.154 | 0.140 | 0.93 | 0 |
| FX | 0.042 | –0.152 | 0.234 | 0.67 | 36.3 |
| FXST | 0.067 | –0.081 | 0.211 | 0.38 | 0 |
| GCC | 0.009 | –0.165 | 0.182 | 0.92 | 22.9 |
| IC | 0.123 | –0.100 | 0.335 | 0.28 | 50.7 |
| IFO | 0.056 | –0.120 | 0.229 | 0.53 | 24.1 |
| PCR | 0.125 | –0.082 | 0.321 | 0.24 | 42.9 |
| PLIC | 0.104 | –0.146 | 0.341 | 0.42 | 60.6 |
| PTR | 0.127 | –0.103 | 0.344 | 0.28 | 53.4 |
| RLIC | 0.176 | –0.067 | 0.400 | 0.15 | 58.6 |
| SCC | 0.050 | –0.098 | 0.195 | 0.51 | 0 |
| SCR | 0.157 | –0.066 | 0.365 | 0.17 | 50.6 |
| SFO | 0.033 | –0.115 | 0.179 | 0.66 | 0 |
| SLF | 0.177 | –0.081 | 0.413 | 0.18 | 63.1 |
| SS | 0.142 | –0.054 | 0.327 | 0.16 | 37.2 |
| UNC | 0.079 | –0.138 | 0.289 | 0.48 | 48.1 |

**Supplementary Table 80** Mega-analysis results of correlation between axial diffusivity (AD) and lithium dose in patients with bipolar disorder

| Region of interest | *β* | 95% CI-lower bound | 95% CI-upper bound | *p* | *I^2^* (%) |
| --- | --- | --- | --- | --- | --- |
| ACR | 0.154 | –0.103 | 0.392 | 0.24 | 62.9 |
| ALIC | –0.005 | –0.152 | 0.142 | 0.94 | 0 |
| Average AD | 0.129 | –0.084 | 0.330 | 0.24 | 46.0 |
| BCC | 0.068 | –0.079 | 0.213 | 0.36 | 0 |
| CC | 0.052 | –0.096 | 0.197 | 0.49 | 0 |
| CGC | 0.094 | –0.054 | 0.237 | 0.21 | 0 |
| CGH | 0.141 | –0.036 | 0.310 | 0.12 | 24.8 |
| CR | 0.107 | –0.074 | 0.281 | 0.25 | 27.3 |
| CST | –0.047 | –0.193 | 0.100 | 0.53 | 0 |
| EC | –0.027 | –0.173 | 0.120 | 0.72 | 0 |
| FX | 0.041 | –0.169 | 0.247 | 0.70 | 44.9 |
| FXST | 0.080 | –0.067 | 0.224 | 0.29 | 0 |
| GCC | 0.149 | –0.167 | 0.437 | 0.36 | 75.5 |
| IC | 0.045 | –0.102 | 0.191 | 0.55 | 0 |
| IFO | 0.020 | –0.127 | 0.167 | 0.79 | 0 |
| PCR | 0.120 | –0.064 | 0.296 | 0.20 | 29.5 |
| PLIC | 0.039 | –0.140 | 0.215 | 0.67 | 25.9 |
| PTR | 0.127 | –0.119 | 0.358 | 0.31 | 59.2 |
| RLIC | 0.074 | –0.073 | 0.219 | 0.32 | 0 |
| SCC | 0.007 | –0.140 | 0.153 | 0.93 | 0 |
| SCR | 0.058 | –0.089 | 0.203 | 0.44 | 0 |
| SFO | –0.006 | –0.153 | 0.141 | 0.94 | 0 |
| SLF | 0.176 | –0.129 | 0.450 | 0.26 | 73.8 |
| SS | 0.129 | –0.039 | 0.290 | 0.13 | 18.1 |
| UNC | 0.014 | –0.196 | 0.224 | 0.89 | 45.7 |

**Supplementary Table 81** Mega-analysis results of correlation between radial diffusivity (RD) and lithium dose in patients with bipolar disorder

| Region of interest | *β* | 95% CI-lower bound | 95% CI-upper bound | *p* | *I^2^* (%) |
| --- | --- | --- | --- | --- | --- |
| ACR | 0.108 | –0.104 | 0.311 | 0.32 | 45.5 |
| ALIC | 0.033 | –0.170 | 0.234 | 0.75 | 41.6 |
| Average RD | 0.079 | –0.074 | 0.228 | 0.31 | 5 |
| BCC | 0.117 | –0.030 | 0.260 | 0.12 | 0 |
| CC | 0.094 | –0.053 | 0.238 | 0.21 | 0 |
| CGC | 0.043 | –0.104 | 0.189 | 0.57 | 0 |
| CGH | 0.064 | –0.169 | 0.290 | 0.59 | 54.8 |
| CR | 0.169 | –0.075 | 0.394 | 0.17 | 58.8 |
| CST | 0.132 | –0.060 | 0.315 | 0.18 | 34.8 |
| EC | 0.010 | –0.140 | 0.160 | 0.90 | 3.2 |
| FX | 0.040 | –0.145 | 0.222 | 0.67 | 30.3 |
| FXST | 0.064 | –0.084 | 0.208 | 0.40 | 0 |
| GCC | –0.003 | –0.150 | 0.144 | 0.97 | 0 |
| IC | 0.128 | –0.094 | 0.338 | 0.26 | 50.0 |
| IFO | 0.036 | –0.124 | 0.195 | 0.66 | 11.7 |
| PCR | 0.074 | –0.111 | 0.254 | 0.44 | 30.2 |
| PLIC | 0.134 | –0.122 | 0.374 | 0.30 | 62.5 |
| PTR | 0.078 | –0.082 | 0.234 | 0.34 | 11.1 |
| RLIC | 0.161 | –0.077 | 0.381 | 0.18 | 56.6 |
| SCC | 0.055 | –0.093 | 0.200 | 0.47 | 0 |
| SCR | 0.198 | –0.076 | 0.444 | 0.15 | 67.4 |
| SFO | 0.053 | –0.099 | 0.201 | 0.50 | 3.5 |
| SLF | 0.143 | –0.066 | 0.340 | 0.18 | 44.2 |
| SS | 0.116 | –0.072 | 0.295 | 0.23 | 31.8 |
| UNC | 0.086 | –0.093 | 0.260 | 0.35 | 25.7 |

**Supplementary Table 82** Mega-analysis results of correlation between fractional anisotropy (FA) and imipramine equivalent dose in patients with major depressive disorder

| Region of interest | *β* | 95% CI-lower bound | 95% CI-upper bound | *p* | *I^2^* (%) |
| --- | --- | --- | --- | --- | --- |
| ACR | –0.078 | –0.177 | 0.024 | 0.13 | 0 |
| ALIC | –0.052 | –0.160 | 0.057 | 0.35 | 8.3 |
| Average FA | –0.114 | –0.213 | –0.013 | 2.7 × 10^–2^ | 0 |
| BCC | –0.113 | –0.218 | –0.004 | 4.2 × 10^–2^ | 7.7 |
| CC | –0.089 | –0.210 | 0.034 | 0.16 | 22.7 |
| CGC | –0.083 | –0.183 | 0.018 | 0.11 | 0 |
| CGH | –0.025 | –0.125 | 0.077 | 0.64 | 0 |
| CR | –0.060 | –0.160 | 0.042 | 0.25 | 0 |
| CST | –0.154 | –0.317 | 0.017 | 7.7 × 10^–2^ | 57.1 |
| EC | –0.112 | –0.211 | –0.011 | 3.0 × 10^–2^ | 0 |
| FX | –0.050 | –0.178 | 0.080 | 0.45 | 28.5 |
| FXST | –0.044 | –0.150 | 0.065 | 0.43 | 7.1 |
| GCC | –0.050 | –0.200 | 0.103 | 0.52 | 46.2 |
| IC | 0.013 | –0.089 | 0.114 | 0.81 | 0 |
| IFO | –0.084 | –0.186 | 0.020 | 0.11 | 3.0 |
| PCR | –0.072 | –0.223 | 0.082 | 0.36 | 47.0 |
| PLIC | 0.046 | –0.055 | 0.147 | 0.37 | 0 |
| PTR | –0.052 | –0.152 | 0.050 | 0.32 | 0 |
| RLIC | 0.033 | –0.069 | 0.133 | 0.53 | 0 |
| SCC | –0.019 | –0.119 | 0.083 | 0.72 | 0 |
| SCR | –0.019 | –0.120 | 0.082 | 0.72 | 0 |
| SFO | –0.095 | –0.194 | 0.006 | 6.6 × 10^–2^ | 0 |
| SLF | –0.053 | –0.153 | 0.049 | 0.31 | 0 |
| SS | –0.106 | –0.205 | –0.005 | 4.0 × 10^–2^ | 0 |
| UNC | 0.059 | –0.042 | 0.159 | 0.25 | 0 |

**Supplementary Table 83** Mega-analysis results of correlation between mean diffusivity (MD) and imipramine equivalent dose in patients with major depressive disorder

| Region of interest | *β* | 95% CI-lower bound | 95% CI-upper bound | *p* | *I^2^* (%) |
| --- | --- | --- | --- | --- | --- |
| ACR | 0.050 | –0.052 | 0.150 | 0.34 | 0 |
| ALIC | 0.001 | –0.100 | 0.102 | 0.99 | 0 |
| Average MD | 0.071 | –0.031 | 0.170 | 0.17 | 0 |
| BCC | 0.065 | –0.036 | 0.165 | 0.21 | 0 |
| CC | 0.049 | –0.052 | 0.149 | 0.34 | 0 |
| CGC | 0.098 | –0.023 | 0.216 | 0.11 | 20.6 |
| CGH | 0.089 | –0.013 | 0.188 | 8.7 × 10^–2^ | 0 |
| CR | 0.035 | –0.066 | 0.136 | 0.49 | 0 |
| CST | 0.106 | –0.089 | 0.294 | 0.29 | 66.7 |
| EC | 0.056 | –0.046 | 0.156 | 0.28 | 0 |
| FX | 0.039 | –0.066 | 0.143 | 0.47 | 3.7 |
| FXST | 0.053 | –0.049 | 0.153 | 0.31 | 0 |
| GCC | 0.052 | –0.049 | 0.153 | 0.31 | 0 |
| IC | 0.033 | –0.068 | 0.134 | 0.52 | 0 |
| IFO | 0.081 | –0.021 | 0.180 | 0.12 | 0 |
| PCR | 0.040 | –0.061 | 0.141 | 0.44 | 0 |
| PLIC | 0.022 | –0.098 | 0.141 | 0.72 | 19.5 |
| PTR | 0.076 | –0.063 | 0.212 | 0.28 | 36.1 |
| RLIC | 0.089 | –0.013 | 0.188 | 8.7 × 10^–2^ | 0 |
| SCC | 0.001 | –0.100 | 0.102 | 0.98 | 0 |
| SCR | 0.022 | –0.080 | 0.122 | 0.68 | 0 |
| SFO | 0.023 | –0.078 | 0.124 | 0.65 | 0 |
| SLF | 0.031 | –0.071 | 0.131 | 0.55 | 0 |
| SS | 0.131 | 0.029 | 0.229 | 1.2 × 10^–2^ | 0.7 |
| UNC | –0.024 | –0.124 | 0.078 | 0.65 | 0 |

**Supplementary Table 84** Mega-analysis results of correlation between axial diffusivity (AD) and imipramine equivalent dose in patients with major depressive disorder

| Region of interest | *β* | 95% CI-lower bound | 95% CI-upper bound | *p* | *I^2^* (%) |
| --- | --- | --- | --- | --- | --- |
| ACR | 0.021 | –0.080 | 0.122 | 0.68 | 0 |
| ALIC | –0.004 | –0.135 | 0.127 | 0.95 | 30.4 |
| Average AD | 0.043 | –0.059 | 0.143 | 0.41 | 0 |
| BCC | –0.025 | –0.173 | 0.124 | 0.74 | 43.9 |
| CC | <0.001 | –0.137 | 0.138 | 1.00 | 35.8 |
| CGC | 0.082 | –0.110 | 0.268 | 0.40 | 65.4 |
| CGH | 0.109 | 0.008 | 0.208 | 3.4 × 10^–2^ | 0 |
| CR | 0.026 | –0.076 | 0.127 | 0.62 | 0 |
| CST | 0.026 | –0.160 | 0.210 | 0.79 | 63.6 |
| EC | 0.015 | –0.086 | 0.116 | 0.77 | 0 |
| FX | 0.031 | –0.071 | 0.132 | 0.55 | 0 |
| FXST | 0.009 | –0.092 | 0.111 | 0.86 | 0 |
| GCC | 0.046 | –0.102 | 0.192 | 0.54 | 43.2 |
| IC | 0.070 | –0.032 | 0.170 | 0.18 | 0 |
| IFO | 0.033 | –0.069 | 0.133 | 0.53 | 0 |
| PCR | 0.035 | –0.066 | 0.136 | 0.50 | 0 |
| PLIC | 0.071 | –0.030 | 0.171 | 0.17 | 0 |
| PTR | 0.121 | –0.101 | 0.332 | 0.28 | 74.3 |
| RLIC | 0.109 | 0.008 | 0.208 | 3.5 × 10^–2^ | 0 |
| SCC | –0.012 | –0.113 | 0.089 | 0.81 | 0 |
| SCR | 0.030 | –0.071 | 0.131 | 0.56 | 0 |
| SFO | –0.001 | –0.102 | 0.101 | 0.99 | 0 |
| SLF | 0.003 | –0.099 | 0.104 | 0.96 | 0 |
| SS | 0.095 | –0.007 | 0.194 | 6.7 × 10^–2^ | 0 |
| UNC | 0.009 | –0.094 | 0.111 | 0.87 | 1.7 |

**Supplementary Table 85** Mega-analysis results of correlation between radial diffusivity (RD) and imipramine equivalent dose in patients with major depressive disorder

| Region of interest | *β* | 95% CI-lower bound | 95% CI-upper bound | *p* | *I^2^* (%) |
| --- | --- | --- | --- | --- | --- |
| ACR | 0.056 | –0.046 | 0.156 | 0.28 | 0 |
| ALIC | 0.001 | –0.100 | 0.103 | 0.98 | 0 |
| Average RD | 0.078 | –0.024 | 0.178 | 0.13 | 0 |
| BCC | 0.081 | –0.020 | 0.181 | 0.11 | 0 |
| CC | 0.059 | –0.043 | 0.159 | 0.26 | 0 |
| CGC | 0.069 | –0.033 | 0.169 | 0.18 | 0 |
| CGH | 0.080 | –0.022 | 0.180 | 0.12 | 0 |
| CR | 0.035 | –0.066 | 0.136 | 0.50 | 0 |
| CST | 0.135 | –0.037 | 0.298 | 0.12 | 57.0 |
| EC | 0.069 | –0.032 | 0.169 | 0.18 | 0 |
| FX | 0.042 | –0.063 | 0.146 | 0.43 | 4.0 |
| FXST | 0.050 | –0.052 | 0.150 | 0.34 | 0 |
| GCC | 0.049 | –0.082 | 0.177 | 0.47 | 29.0 |
| IC | 0.003 | –0.098 | 0.104 | 0.95 | 0 |
| IFO | 0.083 | –0.018 | 0.183 | 0.11 | 0 |
| PCR | 0.033 | –0.068 | 0.134 | 0.52 | 0 |
| PLIC | –0.020 | –0.134 | 0.094 | 0.73 | 14.0 |
| PTR | 0.051 | –0.051 | 0.151 | 0.33 | 0 |
| RLIC | 0.047 | –0.054 | 0.148 | 0.36 | 0 |
| SCC | <0.001 | –0.101 | 0.102 | 0.99 | 0 |
| SCR | 0.011 | –0.090 | 0.112 | 0.83 | 0 |
| SFO | 0.041 | –0.061 | 0.141 | 0.43 | 0 |
| SLF | 0.037 | –0.065 | 0.137 | 0.48 | 0 |
| SS | 0.129 | 0.019 | 0.237 | 2.2 × 10^–2^ | 10.1 |
| UNC | –0.032 | –0.133 | 0.069 | 0.53 | 0 |

**Abbreviations of Supplementary Table 74–85**: ACR, anterior corona radiata; AD, axial diffusivity; ALIC, anterior limb of internal capsule; BCC, body of corpus callosum; CC, corpus callosum; CGC, cingulum (cingulate gyrus); CGH, cingulum (hippocampus); CR, corona radiata; CST, corticospinal tract; EC, external capsule; FA, fractional anisotropy; FX, fornix; FX/ST, fornix (crus)/stria terminalis; GCC, genu of corpus callosum; IC, internal capsule; IFO, inferior fronto-occipital fasciculus; MD, mean diffusivity; PCR, posterior corona radiata; PLIC, posterior limb of internal capsule; PTR, posterior thalamic radiation; RD, radial diffusivity; RLIC, retrolenticular part of internal capsule; SCC, splenium of corpus callosum; SCR, superior corona radiata; SFO, superior fronto-occipital fasciculus; SLF, superior longitudinal fasciculus; SS, sagittal stratum; UNC, uncinate fasciculus.

**SUPPLEMENTARY FIGURES**

**Supplementary Figure 1** Differences in mean diffusivity (MD) between patients with schizophrenia (SZ) and healthy comparison subjects (HCS)

**Supplementary Figure 2** Differences in axial diffusivity (AD) between patients with schizophrenia (SZ) and healthy comparison subjects (HCS)

**Supplementary Figure 3** Differences in radial diffusivity (RD) between patients with schizophrenia (SZ) and healthy comparison subjects (HCS)

**Abbreviations of Supplementary Figure 1–3**: COCORO, Cognitive Genetics Collaborative Research Organization; Enhancing Neuroimaging Genetics through Meta-Analysis consortium-Schizophrenia Diffusion tensor imaging, ENIGMA-Schizophrenia DTI; ACR, anterior corona radiata; ALIC, anterior limb of internal capsule; BCC, body of corpus callosum; CC, corpus callosum; CGC, cingulum (cingulate gyrus); CGH, cingulum (hippocampus); CR, corona radiata; CST, corticospinal tract; EC, external capsule; FX, fornix; FX/ST, fornix (crus)/stria terminalis; GCC, genu of corpus callosum; IC, internal capsule; IFO, inferior fronto-occipital fasciculus; PCR, posterior corona radiata; PLIC, posterior limb of internal capsule; PTR, posterior thalamic radiation; RLIC, retrolenticular part of internal capsule; SCC, splenium of corpus callosum; SCR, superior corona radiata; SFO, superior fronto-occipital fasciculus; SLF, superior longitudinal fasciculus; SS, sagittal stratum; UNC, uncinate fasciculus.**Supplementary Figure 4** Effect size of the DTI indices between patients with psychiatric disorders and healthy comparison subjects in each white matter regions other than main findings

Legends: Significant regions after adjusting for multiple regions tested [*p* < 0.002 (0.05/25)] are highlighted in orange.

Abbreviations: DTI, Diffusion tensor imaging; HCS, healthy comparison subjects; SZ, schizophrenia; BPD, bipolar disorder; ASD, autism spectrum disorder; MDD, major depressive disorder; FA, fractional anisotropy; MD, mean diffusivity; AD, axial diffusivity; RD, radial diffusivity.

**Supplementary Figure 5** Forest plot of effect sizes for each cohort for differences of DTI indices in the patients with psychiatric disorders versus healthy comparison subjects

FA in the anterior corona radiata (ACR) in SZ vs HCS

FA in the anterior limb of internal capsule (ALIC) in SZ vs HCS

FA in the average in SZ vs HCS

FA in the body of corpus callosum (BCC) in SZ vs HCS

FA in the corpus callosum (CC) in SZ vs HCS

FA in the cingulum (cingulate gyrus; CGC) in SZ vs HCS

FA in the cingulum (hippocampus; CGH) in SZ vs HCS

FA in the corona radiata (CR) in SZ vs HCS

FA in the corticospinal tract (CST) in SZ vs HCS

FA in the external capsule (EC) in SZ vs HCS

FA in the fornix (FX) in SZ vs HCS

FA in the fornix (crus)/stria terminalis (FXST) in SZ vs HCS

FA in the genu of corpus callosum (GCC) in SZ vs HCS

FA in the internal capsule (IC) in SZ vs HCS

FA in the inferior fronto-occipital fasciculus (IFO) in SZ vs HCS

FA in the posterior corona radiata (PCR) in SZ vs HCS

FA in the posterior limb of internal capsule (PLIC) in SZ vs HCS

FA in the posterior thalamic radiation (PTR) in SZ vs HCS

FA in the retrolenticular part of internal capsule (RLIC) in SZ vs HCS

FA in the splenium of corpus callosum (SCC) in SZ vs HCS

FA in the superior corona radiata (SCR) in SZ vs HCS

FA in the superior fronto-occipital fasciculus (SFO) in SZ vs HCS

FA in the superior longitudinal fasciculus (SLF) in SZ vs HCS

FA in the sagittal stratum (SS) in SZ vs HCS

FA in the uncinate fasciculus (UNC) in SZ vs HCS

MD in the anterior corona radiata (ACR) in SZ vs HCS

MD in the anterior limb of internal capsule (ALIC) in SZ vs HCS

MD in the average in SZ vs HCS

MD in the body of corpus callosum (BCC) in SZ vs HCS

MD in the corpus callosum (CC) in SZ vs HCS

MD in the cingulum (cingulate gyrus; CGC) in SZ vs HCS

MD in the cingulum (hippocampus; CGH) in SZ vs HCS

MD in the corona radiata (CR) in SZ vs HCS

MD in the corticospinal tract (CST) in SZ vs HCS

MD in the external capsule (EC) in SZ vs HCS

MD in the fornix (FX) in SZ vs HCS

MD in the fornix (crus)/stria terminalis (FXST) in SZ vs HCS

MD in the genu of corpus callosum (GCC) in SZ vs HCS

MD in the internal capsule (IC) in SZ vs HCS

MD in the inferior fronto-occipital fasciculus (IFO) in SZ vs HCS

MD in the posterior corona radiata (PCR) in SZ vs HCS

MD in the posterior limb of internal capsule (PLIC) in SZ vs HCS

MD in the posterior thalamic radiation (PTR) in SZ vs HCS

MD in the retrolenticular part of internal capsule (RLIC) in SZ vs HCS

MD in the splenium of corpus callosum (SCC) in SZ vs HCS

MD in the superior corona radiata (SCR) in SZ vs HCS

MD in the superior fronto-occipital fasciculus (SFO) in SZ vs HCS

MD in the superior longitudinal fasciculus (SLF) in SZ vs HCS

MD in the sagittal stratum (SS) in SZ vs HCS

MD in the uncinate fasciculus (UNC) in SZ vs HCS

AD in the anterior corona radiata (ACR) in SZ vs HCS

AD in the anterior limb of internal capsule (ALIC) in SZ vs HCS

AD in the average in SZ vs HCS

AD in the body of corpus callosum (BCC) in SZ vs HCS

AD in the corpus callosum (CC) in SZ vs HCS

AD in the cingulum (cingulate gyrus; CGC) in SZ vs HCS

AD in the cingulum (hippocampus; CGH) in SZ vs HCS

AD in the corona radiata (CR) in SZ vs HCS

AD in the corticospinal tract (CST) in SZ vs HCS

AD in the external capsule (EC) in SZ vs HCS

AD in the fornix (FX) in SZ vs HCS

AD in the fornix (crus)/stria terminalis (FXST) in SZ vs HCS

AD in the genu of corpus callosum (GCC) in SZ vs HCS

AD in the internal capsule (IC) in SZ vs HCS

AD in the inferior fronto-occipital fasciculus (IFO) in SZ vs HCS

AD in the posterior corona radiata (PCR) in SZ vs HCS

AD in the posterior limb of internal capsule (PLIC) in SZ vs HCS

AD in the posterior thalamic radiation (PTR) in SZ vs HCS

AD in the retrolenticular part of internal capsule (RLIC) in SZ vs HCS

AD in the splenium of corpus callosum (SCC) in SZ vs HCS

AD in the superior corona radiata (SCR) in SZ vs HCS

AD in the superior fronto-occipital fasciculus (SFO) in SZ vs HCS

AD in the superior longitudinal fasciculus (SLF) in SZ vs HCS

AD in the sagittal stratum (SS) in SZ vs HCS

AD in the uncinate fasciculus (UNC) in SZ vs HCS

RD in the anterior corona radiata (ACR) in SZ vs HCS

RD in the anterior limb of internal capsule (ALIC) in SZ vs HCS

RD in the average in SZ vs HCS

RD in the body of corpus callosum (BCC) in SZ vs HCS

RD in the corpus callosum (CC) in SZ vs HCS

RD in the cingulum (cingulate gyrus; CGC) in SZ vs HCS

RD in the cingulum (hippocampus; CGH) in SZ vs HCS

RD in the corona radiata (CR) in SZ vs HCS

RD in the corticospinal tract (CST) in SZ vs HCS

RD in the external capsule (EC) in SZ vs HCS

RD in the fornix (FX) in SZ vs HCS

RD in the fornix (crus)/stria terminalis (FXST) in SZ vs HCS

RD in the genu of corpus callosum (GCC) in SZ vs HCS

RD in the internal capsule (IC) in SZ vs HCS

RD in the inferior fronto-occipital fasciculus (IFO) in SZ vs HCS

RD in the posterior corona radiata (PCR) in SZ vs HCS

RD in the posterior limb of internal capsule (PLIC) in SZ vs HCS

RD in the posterior thalamic radiation (PTR) in SZ vs HCS

RD in the retrolenticular part of internal capsule (RLIC) in SZ vs HCS

RD in the splenium of corpus callosum (SCC) in SZ vs HCS

RD in the superior corona radiata (SCR) in SZ vs HCS

RD in the superior fronto-occipital fasciculus (SFO) in SZ vs HCS

RD in the superior longitudinal fasciculus (SLF) in SZ vs HCS

RD in the sagittal stratum (SS) in SZ vs HCS

RD in the uncinate fasciculus (UNC) in SZ vs HCS

FA in the anterior corona radiata (ACR) in BPD vs HCS

FA in the anterior limb of internal capsule (ALIC) in BPD vs HCS

FA in the average in BPD vs HCS

FA in the body of corpus callosum (BCC) in BPD vs HCS

FA in the corpus callosum (CC) in BPD vs HCS

FA in the cingulum (cingulate gyrus; CGC) in BPD vs HCS

FA in the cingulum (hippocampus; CGH) in BPD vs HCS

FA in the corona radiata (CR) in BPD vs HCS

FA in the corticospinal tract (CST) in BPD vs HCS

FA in the external capsule (EC) in BPD vs HCS

FA in the fornix (FX) in BPD vs HCS

FA in the fornix (crus)/stria terminalis (FXST) in BPD vs HCS

FA in the genu of corpus callosum (GCC) in BPD vs HCS

FA in the internal capsule (IC) in BPD vs HCS

FA in the inferior fronto-occipital fasciculus (IFO) in BPD vs HCS

FA in the posterior corona radiata (PCR) in BPD vs HCS

FA in the posterior limb of internal capsule (PLIC) in BPD vs HCS

FA in the posterior thalamic radiation (PTR) in BPD vs HCS

FA in the retrolenticular part of internal capsule (RLIC) in BPD vs HCS

FA in the splenium of corpus callosum (SCC) in BPD vs HCS

FA in the superior corona radiata (SCR) in BPD vs HCS

FA in the superior fronto-occipital fasciculus (SFO) in BPD vs HCS

FA in the superior longitudinal fasciculus (SLF) in BPD vs HCS

FA in the sagittal stratum (SS) in BPD vs HCS

FA in the uncinate fasciculus (UNC) in BPD vs HCS

MD in the anterior corona radiata (ACR) in BPD vs HCS

MD in the anterior limb of internal capsule (ALIC) in BPD vs HCS

MD in the average in BPD vs HCS

MD in the body of corpus callosum (BCC) in BPD vs HCS

MD in the corpus callosum (CC) in BPD vs HCS

MD in the cingulum (cingulate gyrus; CGC) in BPD vs HCS

MD in the cingulum (hippocampus; CGH) in BPD vs HCS

MD in the corona radiata (CR) in BPD vs HCS

MD in the corticospinal tract (CST) in BPD vs HCS

MD in the external capsule (EC) in BPD vs HCS

MD in the fornix (FX) in BPD vs HCS

MD in the fornix (crus)/stria terminalis (FXST) in BPD vs HCS

MD in the genu of corpus callosum (GCC) in BPD vs HCS

MD in the internal capsule (IC) in BPD vs HCS

MD in the inferior fronto-occipital fasciculus (IFO) in BPD vs HCS

MD in the posterior corona radiata (PCR) in BPD vs HCS

MD in the posterior limb of internal capsule (PLIC) in BPD vs HCS

MD in the posterior thalamic radiation (PTR) in BPD vs HCS

MD in the retrolenticular part of internal capsule (RLIC) in BPD vs HCS

MD in the splenium of corpus callosum (SCC) in BPD vs HCS

MD in the superior corona radiata (SCR) in BPD vs HCS

MD in the superior fronto-occipital fasciculus (SFO) in BPD vs HCS

MD in the superior longitudinal fasciculus (SLF) in BPD vs HCS

MD in the sagittal stratum (SS) in BPD vs HCS

MD in the uncinate fasciculus (UNC) in BPD vs HCS

AD in the anterior corona radiata (ACR) in BPD vs HCS

AD in the anterior limb of internal capsule (ALIC) in BPD vs HCS

AD in the average in BPD vs HCS

AD in the body of corpus callosum (BCC) in BPD vs HCS

AD in the corpus callosum (CC) in BPD vs HCS

AD in the cingulum (cingulate gyrus; CGC) in BPD vs HCS

AD in the cingulum (hippocampus; CGH) in BPD vs HCS

AD in the corona radiata (CR) in BPD vs HCS

AD in the corticospinal tract (CST) in BPD vs HCS

AD in the external capsule (EC) in BPD vs HCS

AD in the fornix (FX) in BPD vs HCS

AD in the fornix (crus)/stria terminalis (FXST) in BPD vs HCS

AD in the genu of corpus callosum (GCC) in BPD vs HCS

AD in the internal capsule (IC) in BPD vs HCS

AD in the inferior fronto-occipital fasciculus (IFO) in BPD vs HCS

AD in the posterior corona radiata (PCR) in BPD vs HCS

AD in the posterior limb of internal capsule (PLIC) in BPD vs HCS

AD in the posterior thalamic radiation (PTR) in BPD vs HCS

AD in the retrolenticular part of internal capsule (RLIC) in BPD vs HCS

AD in the splenium of corpus callosum (SCC) in BPD vs HCS

AD in the superior corona radiata (SCR) in BPD vs HCS

AD in the superior fronto-occipital fasciculus (SFO) in BPD vs HCS

AD in the superior longitudinal fasciculus (SLF) in BPD vs HCS

AD in the sagittal stratum (SS) in BPD vs HCS

AD in the uncinate fasciculus (UNC) in BPD vs HCS

RD in the anterior corona radiata (ACR) in BPD vs HCS

RD in the anterior limb of internal capsule (ALIC) in BPD vs HCS

RD in the average in BPD vs HCS

RD in the body of corpus callosum (BCC) in BPD vs HCS

RD in the corpus callosum (CC) in BPD vs HCS

RD in the cingulum (cingulate gyrus; CGC) in BPD vs HCS

RD in the cingulum (hippocampus; CGH) in BPD vs HCS

RD in the corona radiata (CR) in BPD vs HCS

RD in the corticospinal tract (CST) in BPD vs HCS

RD in the external capsule (EC) in BPD vs HCS

RD in the fornix (FX) in BPD vs HCS

RD in the fornix (crus)/stria terminalis (FXST) in BPD vs HCS

RD in the genu of corpus callosum (GCC) in BPD vs HCS

RD in the internal capsule (IC) in BPD vs HCS

RD in the inferior fronto-occipital fasciculus (IFO) in BPD vs HCS

RD in the posterior corona radiata (PCR) in BPD vs HCS

RD in the posterior limb of internal capsule (PLIC) in BPD vs HCS

RD in the posterior thalamic radiation (PTR) in BPD vs HCS

RD in the retrolenticular part of internal capsule (RLIC) in BPD vs HCS

RD in the splenium of corpus callosum (SCC) in BPD vs HCS

RD in the superior corona radiata (SCR) in BPD vs HCS

RD in the superior fronto-occipital fasciculus (SFO) in BPD vs HCS

RD in the superior longitudinal fasciculus (SLF) in BPD vs HCS

RD in the sagittal stratum (SS) in BPD vs HCS

RD in the uncinate fasciculus (UNC) in BPD vs HCS

FA in the anterior corona radiata (ACR) in ASD vs HCS

FA in the anterior limb of internal capsule (ALIC) in ASD vs HCS

FA in the average in ASD vs HCS

FA in the body of corpus callosum (BCC) in ASD vs HCS

FA in the corpus callosum (CC) in ASD vs HCS

FA in the cingulum (cingulate gyrus; CGC) in ASD vs HCS

FA in the cingulum (hippocampus; CGH) in ASD vs HCS

FA in the corona radiata (CR) in ASD vs HCS

FA in the corticospinal tract (CST) in ASD vs HCS

FA in the external capsule (EC) in ASD vs HCS

FA in the fornix (FX) in ASD vs HCS

FA in the fornix (crus)/stria terminalis (FXST) in ASD vs HCS

FA in the genu of corpus callosum (GCC) in ASD vs HCS

FA in the internal capsule (IC) in ASD vs HCS

FA in the inferior fronto-occipital fasciculus (IFO) in ASD vs HCS

FA in the posterior corona radiata (PCR) in ASD vs HCS

FA in the posterior limb of internal capsule (PLIC) in ASD vs HCS

FA in the posterior thalamic radiation (PTR) in ASD vs HCS

FA in the retrolenticular part of internal capsule (RLIC) in ASD vs HCS

FA in the splenium of corpus callosum (SCC) in ASD vs HCS

FA in the superior corona radiata (SCR) in ASD vs HCS

FA in the superior fronto-occipital fasciculus (SFO) in ASD vs HCS

FA in the superior longitudinal fasciculus (SLF) in ASD vs HCS

FA in the sagittal stratum (SS) in ASD vs HCS

FA in the uncinate fasciculus (UNC) in ASD vs HCS

MD in the anterior corona radiata (ACR) in ASD vs HCS

MD in the anterior limb of internal capsule (ALIC) in ASD vs HCS

MD in the average in ASD vs HCS

MD in the body of corpus callosum (BCC) in ASD vs HCS

MD in the corpus callosum (CC) in ASD vs HCS

MD in the cingulum (cingulate gyrus; CGC) in ASD vs HCS

MD in the cingulum (hippocampus; CGH) in ASD vs HCS

MD in the corona radiata (CR) in ASD vs HCS

MD in the corticospinal tract (CST) in ASD vs HCS

MD in the external capsule (EC) in ASD vs HCS

MD in the fornix (FX) in ASD vs HCS

MD in the fornix (crus)/stria terminalis (FXST) in ASD vs HCS

MD in the genu of corpus callosum (GCC) in ASD vs HCS

MD in the internal capsule (IC) in ASD vs HCS

MD in the inferior fronto-occipital fasciculus (IFO) in ASD vs HCS

MD in the posterior corona radiata (PCR) in ASD vs HCS

MD in the posterior limb of internal capsule (PLIC) in ASD vs HCS

MD in the posterior thalamic radiation (PTR) in ASD vs HCS

MD in the retrolenticular part of internal capsule (RLIC) in ASD vs HCS

MD in the splenium of corpus callosum (SCC) in ASD vs HCS

MD in the superior corona radiata (SCR) in ASD vs HCS

MD in the superior fronto-occipital fasciculus (SFO) in ASD vs HCS

MD in the superior longitudinal fasciculus (SLF) in ASD vs HCS

MD in the sagittal stratum (SS) in ASD vs HCS

MD in the uncinate fasciculus (UNC) in ASD vs HCS

AD in the anterior corona radiata (ACR) in ASD vs HCS

AD in the anterior limb of internal capsule (ALIC) in ASD vs HCS

AD in the average in ASD vs HCS

AD in the body of corpus callosum (BCC) in ASD vs HCS

AD in the corpus callosum (CC) in ASD vs HCS

AD in the cingulum (cingulate gyrus; CGC) in ASD vs HCS

AD in the cingulum (hippocampus; CGH) in ASD vs HCS

AD in the corona radiata (CR) in ASD vs HCS

AD in the corticospinal tract (CST) in ASD vs HCS

AD in the external capsule (EC) in ASD vs HCS

AD in the fornix (FX) in ASD vs HCS

AD in the fornix (crus)/stria terminalis (FXST) in ASD vs HCS

AD in the genu of corpus callosum (GCC) in ASD vs HCS

AD in the internal capsule (IC) in ASD vs HCS

AD in the inferior fronto-occipital fasciculus (IFO) in ASD vs HCS

AD in the posterior corona radiata (PCR) in ASD vs HCS

AD in the posterior limb of internal capsule (PLIC) in ASD vs HCS

AD in the posterior thalamic radiation (PTR) in ASD vs HCS

AD in the retrolenticular part of internal capsule (RLIC) in ASD vs HCS

AD in the splenium of corpus callosum (SCC) in ASD vs HCS

AD in the superior corona radiata (SCR) in ASD vs HCS

AD in the superior fronto-occipital fasciculus (SFO) in ASD vs HCS

AD in the superior longitudinal fasciculus (SLF) in ASD vs HCS

AD in the sagittal stratum (SS) in ASD vs HCS

AD in the uncinate fasciculus (UNC) in ASD vs HCS

RD in the anterior corona radiata (ACR) in ASD vs HCS

RD in the anterior limb of internal capsule (ALIC) in ASD vs HCS

RD in the average in ASD vs HCS

RD in the body of corpus callosum (BCC) in ASD vs HCS

RD in the corpus callosum (CC) in ASD vs HCS

RD in the cingulum (cingulate gyrus; CGC) in ASD vs HCS

RD in the cingulum (hippocampus; CGH) in ASD vs HCS

RD in the corona radiata (CR) in ASD vs HCS

RD in the corticospinal tract (CST) in ASD vs HCS

RD in the external capsule (EC) in ASD vs HCS

RD in the fornix (FX) in ASD vs HCS

RD in the fornix (crus)/stria terminalis (FXST) in ASD vs HCS

RD in the genu of corpus callosum (GCC) in ASD vs HCS

RD in the internal capsule (IC) in ASD vs HCS

RD in the inferior fronto-occipital fasciculus (IFO) in ASD vs HCS

RD in the posterior corona radiata (PCR) in ASD vs HCS

RD in the posterior limb of internal capsule (PLIC) in ASD vs HCS

RD in the posterior thalamic radiation (PTR) in ASD vs HCS

RD in the retrolenticular part of internal capsule (RLIC) in ASD vs HCS

RD in the splenium of corpus callosum (SCC) in ASD vs HCS

RD in the superior corona radiata (SCR) in ASD vs HCS

RD in the superior fronto-occipital fasciculus (SFO) in ASD vs HCS

RD in the superior longitudinal fasciculus (SLF) in ASD vs HCS

RD in the sagittal stratum (SS) in ASD vs HCS

RD in the uncinate fasciculus (UNC) in ASD vs HCS

FA in the anterior corona radiata (ACR) in MDD vs HCS

FA in the anterior limb of internal capsule (ALIC) in MDD vs HCS

FA in the average in MDD vs HCS

FA in the body of corpus callosum (BCC) in MDD vs HCS

FA in the corpus callosum (CC) in MDD vs HCS

FA in the cingulum (cingulate gyrus; CGC) in MDD vs HCS

FA in the cingulum (hippocampus; CGH) in MDD vs HCS

FA in the corona radiata (CR) in MDD vs HCS

FA in the corticospinal tract (CST) in MDD vs HCS

FA in the external capsule (EC) in MDD vs HCS

FA in the fornix (FX) in MDD vs HCS

FA in the fornix (crus)/stria terminalis (FXST) in MDD vs HCS

FA in the genu of corpus callosum (GCC) in MDD vs HCS

FA in the internal capsule (IC) in MDD vs HCS

FA in the inferior fronto-occipital fasciculus (IFO) in MDD vs HCS

FA in the posterior corona radiata (PCR) in MDD vs HCS

FA in the posterior limb of internal capsule (PLIC) in MDD vs HCS

FA in the posterior thalamic radiation (PTR) in MDD vs HCS

FA in the retrolenticular part of internal capsule (RLIC) in MDD vs HCS

FA in the splenium of corpus callosum (SCC) in MDD vs HCS

FA in the superior corona radiata (SCR) in MDD vs HCS

FA in the superior fronto-occipital fasciculus (SFO) in MDD vs HCS

FA in the superior longitudinal fasciculus (SLF) in MDD vs HCS

FA in the sagittal stratum (SS) in MDD vs HCS

FA in the uncinate fasciculus (UNC) in MDD vs HCS

MD in the anterior corona radiata (ACR) in MDD vs HCS

MD in the anterior limb of internal capsule (ALIC) in MDD vs HCS

MD in the average in MDD vs HCS

MD in the body of corpus callosum (BCC) in MDD vs HCS

MD in the corpus callosum (CC) in MDD vs HCS

MD in the cingulum (cingulate gyrus; CGC) in MDD vs HCS

MD in the cingulum (hippocampus; CGH) in MDD vs HCS

MD in the corona radiata (CR) in MDD vs HCS

MD in the corticospinal tract (CST) in MDD vs HCS

MD in the external capsule (EC) in MDD vs HCS

MD in the fornix (FX) in MDD vs HCS

MD in the fornix (crus)/stria terminalis (FXST) in MDD vs HCS

MD in the genu of corpus callosum (GCC) in MDD vs HCS

MD in the internal capsule (IC) in MDD vs HCS

MD in the inferior fronto-occipital fasciculus (IFO) in MDD vs HCS

MD in the posterior corona radiata (PCR) in MDD vs HCS

MD in the posterior limb of internal capsule (PLIC) in MDD vs HCS

MD in the posterior thalamic radiation (PTR) in MDD vs HCS

MD in the retrolenticular part of internal capsule (RLIC) in MDD vs HCS

MD in the splenium of corpus callosum (SCC) in MDD vs HCS

MD in the superior corona radiata (SCR) in MDD vs HCS

MD in the superior fronto-occipital fasciculus (SFO) in MDD vs HCS

MD in the superior longitudinal fasciculus (SLF) in MDD vs HCS

MD in the sagittal stratum (SS) in MDD vs HCS

MD in the uncinate fasciculus (UNC) in MDD vs HCS

AD in the anterior corona radiata (ACR) in MDD vs HCS

AD in the anterior limb of internal capsule (ALIC) in MDD vs HCS

AD in the average in MDD vs HCS

AD in the body of corpus callosum (BCC) in MDD vs HCS

AD in the corpus callosum (CC) in MDD vs HCS

AD in the cingulum (cingulate gyrus; CGC) in MDD vs HCS

AD in the cingulum (hippocampus; CGH) in MDD vs HCS

AD in the corona radiata (CR) in MDD vs HCS

AD in the corticospinal tract (CST) in MDD vs HCS

AD in the external capsule (EC) in MDD vs HCS

AD in the fornix (FX) in MDD vs HCS

AD in the fornix (crus)/stria terminalis (FXST) in MDD vs HCS

AD in the genu of corpus callosum (GCC) in MDD vs HCS

AD in the internal capsule (IC) in MDD vs HCS

AD in the inferior fronto-occipital fasciculus (IFO) in MDD vs HCS

AD in the posterior corona radiata (PCR) in MDD vs HCS

AD in the posterior limb of internal capsule (PLIC) in MDD vs HCS

AD in the posterior thalamic radiation (PTR) in MDD vs HCS

AD in the retrolenticular part of internal capsule (RLIC) in MDD vs HCS

AD in the splenium of corpus callosum (SCC) in MDD vs HCS

AD in the superior corona radiata (SCR) in MDD vs HCS

AD in the superior fronto-occipital fasciculus (SFO) in MDD vs HCS

AD in the superior longitudinal fasciculus (SLF) in MDD vs HCS

AD in the sagittal stratum (SS) in MDD vs HCS

AD in the uncinate fasciculus (UNC) in MDD vs HCS

RD in the anterior corona radiata (ACR) in MDD vs HCS

RD in the anterior limb of internal capsule (ALIC) in MDD vs HCS

RD in the average in MDD vs HCS

RD in the body of corpus callosum (BCC) in MDD vs HCS

RD in the corpus callosum (CC) in MDD vs HCS

RD in the cingulum (cingulate gyrus; CGC) in MDD vs HCS

RD in the cingulum (hippocampus; CGH) in MDD vs HCS

RD in the corona radiata (CR) in MDD vs HCS

RD in the corticospinal tract (CST) in MDD vs HCS

RD in the external capsule (EC) in MDD vs HCS

RD in the fornix (FX) in MDD vs HCS

RD in the fornix (crus)/stria terminalis (FXST) in MDD vs HCS

RD in the genu of corpus callosum (GCC) in MDD vs HCS

RD in the internal capsule (IC) in MDD vs HCS

RD in the inferior fronto-occipital fasciculus (IFO) in MDD vs HCS

RD in the posterior corona radiata (PCR) in MDD vs HCS

RD in the posterior limb of internal capsule (PLIC) in MDD vs HCS

RD in the posterior thalamic radiation (PTR) in MDD vs HCS

RD in the retrolenticular part of internal capsule (RLIC) in MDD vs HCS

RD in the splenium of corpus callosum (SCC) in MDD vs HCS

RD in the superior corona radiata (SCR) in MDD vs HCS

RD in the superior fronto-occipital fasciculus (SFO) in MDD vs HCS

RD in the superior longitudinal fasciculus (SLF) in MDD vs HCS

RD in the sagittal stratum (SS) in MDD vs HCS

RD in the uncinate fasciculus (UNC) in MDD vs HCS

Abbreviations: DTI, Diffusion tensor imaging; HCS, healthy comparison subjects; SZ, schizophrenia; BPD, bipolar disorder; ASD, autism spectrum disorder; MDD, major depressive disorder; FA, fractional anisotropy; MD, mean diffusivity; AD, axial diffusivity; RD, radial diffusivity.

**Supplementary Figure 6** Differences in fractional anisotropy (FA) between patients with bipolar disorder (BPD) and healthy comparison subjects (HCS)

**Supplementary Figure 7** Differences in mean diffusivity (MD) between patients with bipolar disorder (BPD) and healthy comparison subjects (HCS)

**Supplementary Figure 8** Differences in axial diffusivity (AD) between patients with bipolar disorder (BPD) and healthy comparison subjects (HCS)

**Supplementary Figure 9** Differences in radial diffusivity (RD) between patients with bipolar disorder (BPD) and healthy comparison subjects (HCS)

**Supplementary Figure 10** Differences in fractional anisotropy (FA) between individuals with autism spectrum disorder (ASD) and healthy comparison subjects (HCS)

**Supplementary Figure 11** Differences in mean diffusivity (MD) between individuals with autism spectrum disorder (ASD) and healthy comparison subjects (HCS)

**Supplementary Figure 12** Differences in axial diffusivity (AD) between individuals with autism spectrum disorder (ASD) and healthy comparison subjects (HCS)

**Supplementary Figure 13** Differences in radial diffusivity (RD) between individuals with autism spectrum disorder (ASD) and healthy comparison subjects (HCS)

**Supplementary Figure 14** Differences in fractional anisotropy (FA) between patients with major depressive disorder (MDD) and healthy comparison subjects (HCS)

**Supplementary Figure 15** Differences in mean diffusivity (MD) between patients with major depressive disorder (MDD) and healthy comparison subjects (HCS)

**Supplementary Figure 16** Differences in axial diffusivity (AD) between patients with major depressive disorder (MDD) and healthy comparison subjects (HCS)

**Supplementary Figure 17** Differences in radial diffusivity (RD) between patients with major depressive disorder (MDD) and healthy comparison subjects (HCS)

**Abbreviations of Supplementary Figure 6–17**: COCORO, Cognitive Genetics Collaborative Research Organization; Enhancing Neuroimaging Genetics through Meta-Analysis consortium-Schizophrenia Diffusion tensor imaging, ENIGMA-Schizophrenia DTI; ACR, anterior corona radiata; ALIC, anterior limb of internal capsule; BCC, body of corpus callosum; CC, corpus callosum; CGC, cingulum (cingulate gyrus); CGH, cingulum (hippocampus); CR, corona radiata; CST, corticospinal tract; EC, external capsule; FX, fornix; FX/ST, fornix (crus)/stria terminalis; GCC, genu of corpus callosum; IC, internal capsule; IFO, inferior fronto-occipital fasciculus; PCR, posterior corona radiata; PLIC, posterior limb of internal capsule; PTR, posterior thalamic radiation; RLIC, retrolenticular part of internal capsule; SCC, splenium of corpus callosum; SCR, superior corona radiata; SFO, superior fronto-occipital fasciculus; SLF, superior longitudinal fasciculus; SS, sagittal stratum; UNC, uncinate fasciculus.
